# Cooperative Reactivity Induced by All-Gallium Coordination at Nickel

**DOI:** 10.1021/acs.inorgchem.5c02296

**Published:** 2025-07-18

**Authors:** Johannes Stephan, Raphael Bühler, Fabrizio E. Napoli, Christian Gemel, Roland A. Fischer

**Affiliations:** † Chair of Inorganic and Metal−Organic Chemistry, Department of Chemistry, TUM School of Natural Sciences, 9184 Technical University of Munich, Lichtenbergstraße 4, D-85748 Garching, Germany; ‡ Catalysis Research Center, Technical University of Munich, Ernst-Otto-Fischer-Straße 1, D-85748 Garching, Germany

## Abstract

Synergistic effects in mixed-metal complexes can lead to small-molecule activation and intriguing reactivity patterns. Herein, we report the dimerization of acetonitrile to a nacnac-type β-diketiminate ligand at the nickel/gallium complex fragment [Ni­(GaCp*)_4_]^2+^ (Cp* = η^5^-C_5_(CH_3_)_5_). The coordination of GaCp* to the Ni­(II) center leads to strong Lewis acidity at the initially Lewis basic Ga­(I) ligand, which resembles an “umpolung” of the Ga center. The electrophilic Ga serves as the site of bond activation. DFT calculations indicate that the proximity of Ni and Ga and a Ga-rich coordination sphere around the Ni atom are essential for facilitating the reaction. Interestingly, we discovered an abnormally large kinetic isotope effect of 28 assigned to proton tunneling.

## Graphical abstract

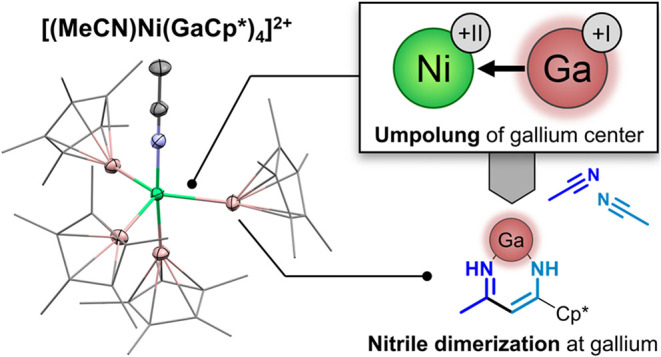

## Introduction

The study of the reactivity of monometallic transition metal (TM) complexes in bond activation reactions has been a cornerstone of chemical research for decades. In this context, bimetallic compounds offer the potential to tackle more challenging substrates and uncover unprecedented reactivity patterns.^1^ Among such systems, complexes with a direct TM–E bond (E = Ga, Al) have garnered significant interest due to their unique ability to tune the reactivity of the TM center. These interactions can basically manifest in two main ways: When a potentially reactive late TM is combined with a low-valent E, the main group element acts as a strong σ-donor. This increases the electron density at the TM, enhancing its reactivity toward bond activation processes. Examples of this behavior include the bimetallic complex [Cp*Rh­(CH_3_)_2_GaCp*], which undergoes intramolecular C–C bond activation of Cp* ligands,^2^ as well as the activation of C–H bonds in benzene or Si–H bonds in triethylsilane for systems such as [M­(AlCp*)_5_] (M = Fe, Ru)^3^ and unsaturated intermediates like [Ni­(AlCp*)_3_][Bibr ref4] or [RuH_2_(GaCp*)_3_].^5^ In contrast, the combination of a highly reactive TM with an E in a higher oxidation state can temper the reactivity of the transition metal. By controlling the TM–E bond distance (e.g., by suitable chelating ligands), the desired reactivity of the TM can be finely tuned for catalytic transformations, which is exemplified by a bimetallic Ni/Ga complex for CO_2_ hydrogenation as well as Rh/Al complex promoting the magnesiation of aryl fluorides.^6–9^

Despite extensive studies on systems in which TM is in a low oxidation state and E either serves as a carbenoid σ-donor or as a Lewis acid, the reverse scenario—combining a TM in a high oxidation state with a low-valent E—still remains largely unexplored (see Scheme 1) although few examples, including Fe^2+^–Al^+^,^10–13^ V^2+^–Al^+^,^14^ or Sm^2+^–Al^+^ ^15^ as element combinations, have been reported. This scarcity arises because the increased electrophilicity of TM triggers unwanted ligand transfer reactions or reduction of the transition metal center.^16^ For example, reactions of low-valent GaCp* with highly electrophilic dicationic first-row TM complexes (e.g., Fe, Co) result in Cp* transfer from gallium to TM in almost all cases, yielding unreactive Cp*-stabilized TM/E species. An exception is [Cu­(GaCp*)_4_]^+^, which retains intact GaCp* ligands rather than TM­(Cp*) moieties.^17^

**Scheme 1:**
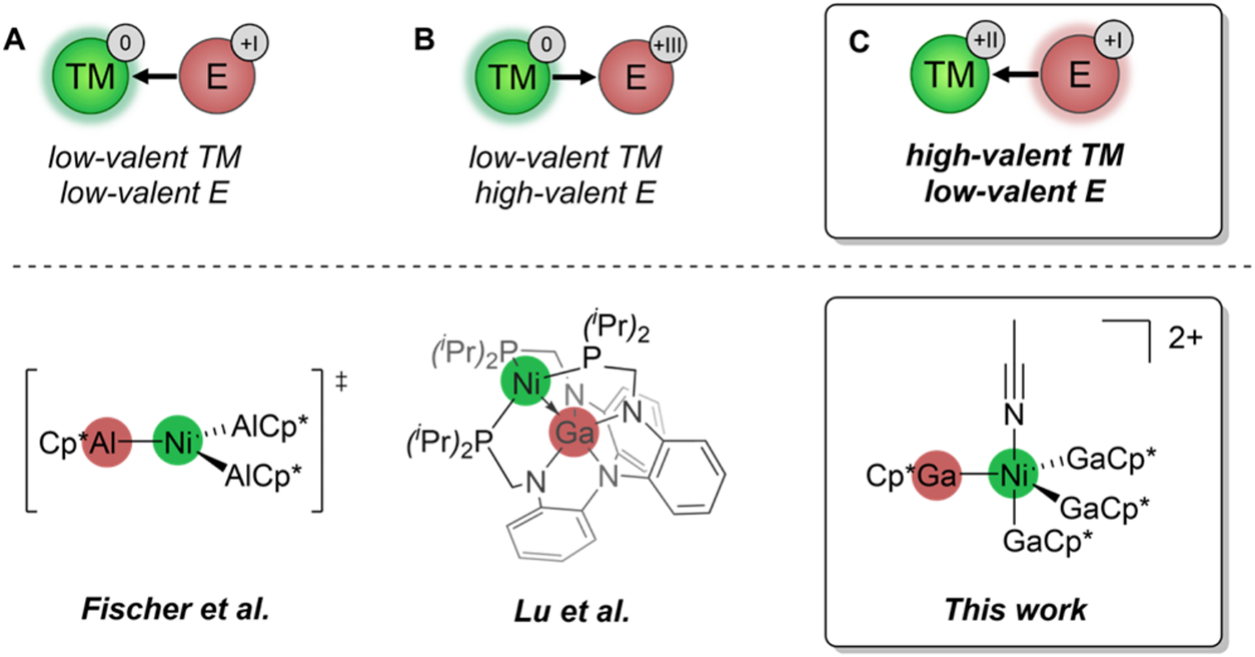
Possible Combinations of Transition Metal (TM) and Main Group Element (E) Ligands^a^

a Low-valent TM and E with E as a σ-donor ligand (**A**), low-valent TM with an oxidized E metal acting as a σ-acceptor ligand (**B**) and high-valent TM with low-valent E binding as a σ-donor ligand to TM (**C**).

In this contribution, we present the first example of a bimetallic Ni/Ga complex that features an “inverse” oxidation state configuration with Ni^2+^ as the oxidized TM and Ga^+^ as the low-valent main group element. The coordination of Ga^+^ to the Ni^2+^ center induces strong Lewis acidity at the gallium site, making Ga the reactive center in bond activation processes. This unique reactivity is exemplified by the dimerization of acetonitrile to form a nacnac-type β-diketiminate ligand at the gallium center.

## Results and Discussion

Reacting [Ni­(MeCN)_6_]­(BAr^F^)_2_ (BAr^F^: tetrakis­[(bis-3,5-trifluoromethyl)­phenyl]­borate) and 5 equiv of GaCp* in 1,2-difluorobenzene as a weakly coordinating solvent immediately affords an intensively purple colored solution. Over the course of several hours, the reaction solution changes its color to a bright, clear orange. Layering the orange reaction solution with *n*-hexane at room temperature yielded orange crystals. Crystals suitable for SC-XRD (single-crystal X-ray diffraction) were obtained by cooling the supernatant solution to −35 °C for several days. Their analysis revealed a bimetallic nickel–gallium complex with the constitutional formula [Ni­(GaCp*)_4_(GaN_2_C_14_H_21_)]­(BAr^F^)_2_ (**1**) (see Figure 1). This compound exhibits one gallium β-diketiminate moiety with a Cp* ligand in its backbone and four GaCp* units coordinated to a nickel atom. Overall, the central nickel atom in **1** adopts a distorted square-pyramidal coordination geometry. Herein, the gallium diketiminate is located in one of the equatorial positions, resulting in one apical (Ga5) and three equatorial GaCp* units (Ga2, Ga3, and Ga4). Altogether, all Ni–GaCp* distances in **1**, ranging from 2.2502(7) Å to 2.2932(8) Å, are in a comparable range to that of the monocationic complex [GaNi­(GaCp*)_4_]­(BAr^F^) (2.2412(7)–2.3199(7) Å).^18^ Compared to the neutral [Ni­(GaCp*)_4_] with Ni–GaCp* bond lengths of 2.2188(5) Å,^19^ the bonds are slightly elongated in **1**. Also, the Ni–Ga distances of 2.2502(7) Å between the central nickel atom and the gallium ketiminate moiety is in a comparable range to that in nickel olefin complexes stabilized by GaDDP as a ligand (2.2439(16)–2.3479(6) Å).^20^ Overall, [Ni­(GaCp*)_4_(GaN_2_C_14_H_21_)]­(BAr^F^)_2_ (**1**) is reproducibly obtained in good yields of 83%.

**Figure 1:**
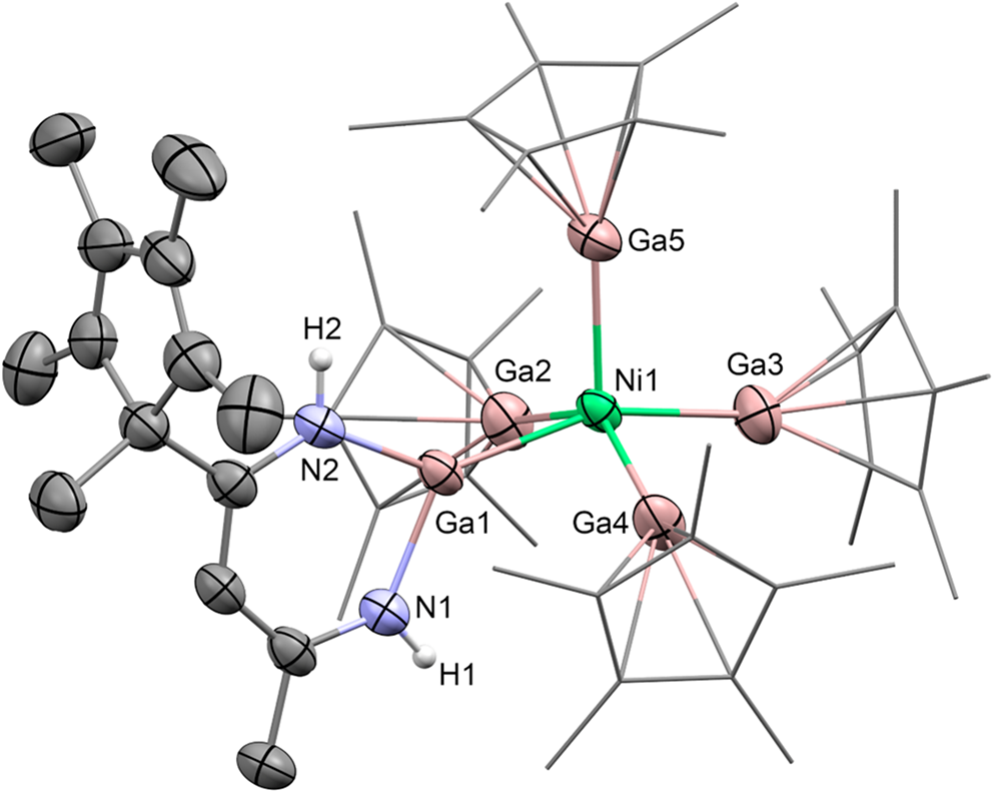
Molecular structure of the dicationic part of **1** as determined by SC-XRD. Ellipsoids are drawn at 50% probability; Cp* ligands are drawn in wireframe representation. Protons except for N–H moieties and BAr^F^ anions as well as cocrystallized solvent molecules have been omitted for clarity.

Structurally similar chelating nacnac-type ligands like in **1** are well-known as stabilizing ligands in low-valent main group chemistry. Examples include GaDDP^21^ (DDP: 2-(2,6-diisopropylphenyl)­amino-4-(2,6-diisopropylphenyl)­imino-2-pentene), Al­(DDP)^22^ or the dimeric complex [Mg­(DDP)]_2_ ^23^ as well as Ga­(I)^24^ and Zn­(I)^25^ centers stabilized by acenaphthene-derived ligands. It is worth mentioning that the β-diketiminate ligand at gallium in **1** features N–H moieties instead of the widely employed aryl-substituted congeners. Besides these examples, Ga­(III) and In­(III) complexes with structurally similar β-diketiminate ligands emerging from the activation of acetonitrile have been reported.^26–28^ However, their synthesis requires elevated temperatures and the aid of halide ions as catalysts.

In order to gain further insights into the formation of [Ni­(GaCp*)_4_(GaN_2_C_14_H_21_)]­(BAr^F^)_2_ (**1**), we isolated the transient, deeply purple colored species by reacting [Ni­(MeCN)_6_]­(BAr^F^)_2_ with 5 equiv GaCp* in dichloromethane as the solvent. Rapid cooling to −80 °C and addition of *n*-hexane result in the precipitation of a deeply purple, almost blackish material. SC-XRD revealed a complex with the constitutional formula [(MeCN)­Ni­(GaCp*)_4_]­(BAr^F^)_2_ (**2**) as the isolated product (see Figure 2). Herein, nickel represents the central atom that is coordinated by four GaCp* moieties as well as one remaining acetonitrile ligand. **2** adopts a slightly distorted pseudotrigonal bipyramidal coordination sphere around the nickel atom with Ga1, Ga2, and Ga3 in the equatorial and N1 as well as Ga4 in the axial positions of the bipyramid.

**Figure 2:**
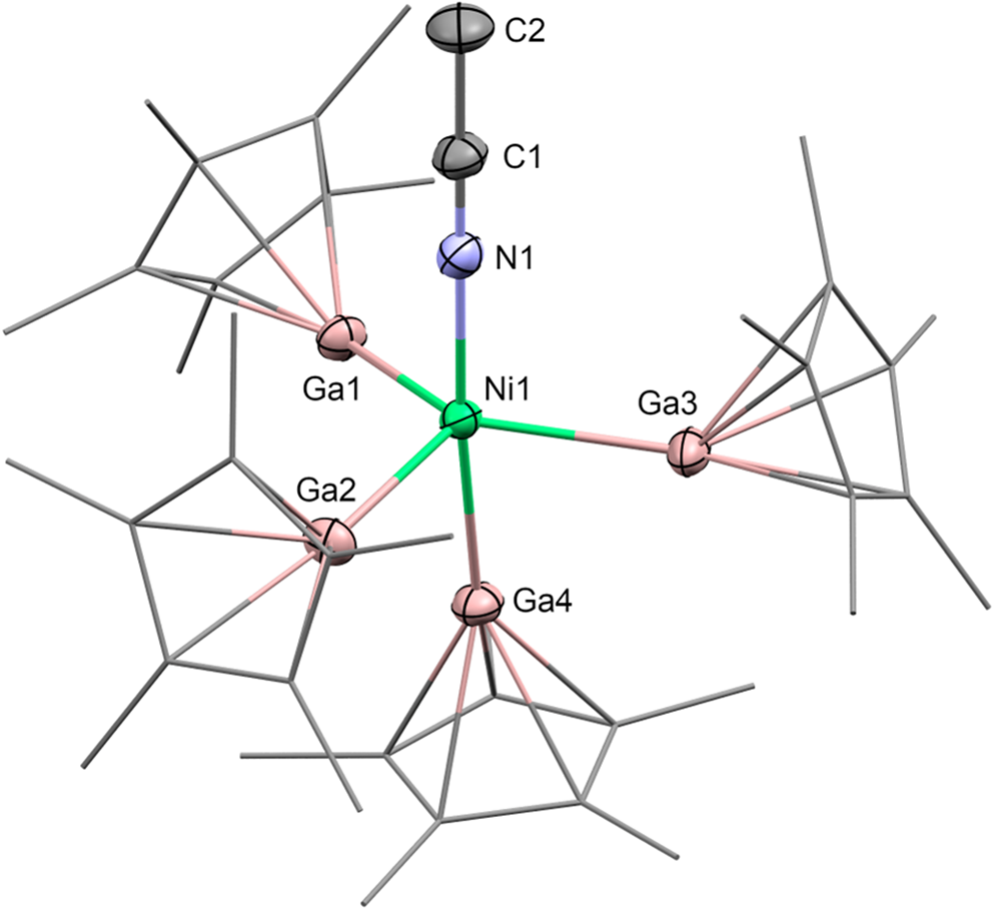
Molecular structure of the dicationic part of **2** as determined by SC-XRD. Ellipsoids are set at 50% probability; Cp* ligands are drawn in wireframe representation. Protons, BAr^F^ anions, and cocrystallized solvent molecules have been omitted for clarity.

That **2** represents indeed an intermediate in the formation of [Ni­(GaCp*)_4_(GaN_2_C_14_H_21_)]­(BAr^F^)_2_ (**1**) is evident from its reaction with GaCp* and acetonitrile, which leads to **1** (see Scheme 2). Isotopic labeling experiments using the deuterated complex [Ni­(MeCN-*d*_3_)_6_]­(BAr^F^)_2_ and GaCp* lead to the deuterated species [Ni­(GaCp*)_4_(GaN_2_C_14_H_15_D_6_)]­(BAr^F^)_2_ (**1** ^**d**^) as the only product. ^1^H NMR spectra of the isolated compound nicely show that the N–H as well as the characteristic methine proton and the methyl group of the nacnac-type ligand disappear due to deuteration (see Figure S19), which indicates tautomerization of the two acetonitrile units. Very surprisingly, there is an abnormally high kinetic isotope effect (KIE) of 28 compared to the nondeuterated complex. This cannot be explained exclusively with the kinetic isotope effect of deuterium vs protium, which should be in a range below or up to 8. Therefore, proton tunneling may play a crucial role in the formation of [Ni­(GaCp*)_4_(GaN_2_C_14_H_21_)]­(BAr^F^)_2_ (**1**).^29^

**Scheme 2:**
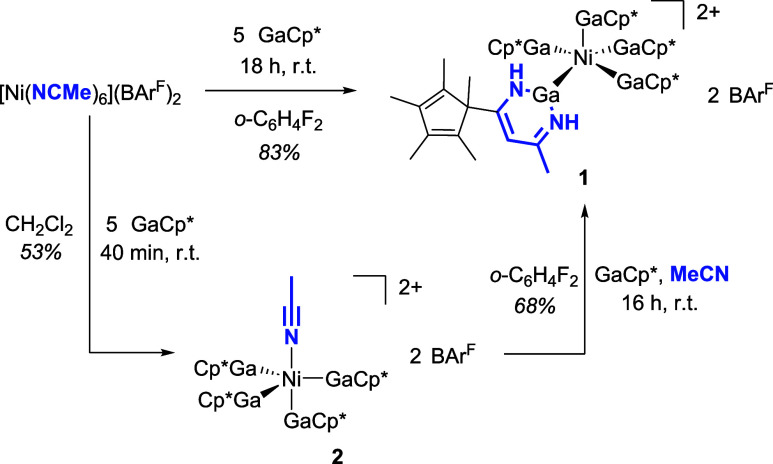
Synthesis of **1** Either as a One-Pot Reaction or via Isolation of Reactive Intermediate **2**

Based on these findings, we tested the feasibility of our mechanistic considerations by using density functional theory calculations. We calculated the Gibbs free energy for potential key intermediates (without BAr^F^ anions) during the dimerization of acetonitrile, starting with [(MeCN)­Ni­(GaCp*)_4_]­(BAr^F^)_2_ (**2**) as the initial species. Therefore, all calculated Gibbs free energies refer to **2**, one free GaCp* moiety, and one acetonitrile moiety. The whole mechanism is discussed in detail in the Supporting Information. Noteworthy, transition state calculations suggest two extraordinarily high barriers (TS2: Δ*G* = 34.6 kcal mol^–1^; TS4: Δ*G* = 44.6 kcal mol^–1^) involving proton transfer steps. We also tested for proton-relay steps, including acetonitrile and 1,2-difluorobenzene acting as proton shuttles to facilitate tautomerization. However, the activation energy barriers increased slightly for acetonitrile (TS2: Δ*G* = 35.5 kcal mol^–1^; TS4: Δ*G* = 48.0 kcal mol^–1^) and significantly for 1,2-difluorobenzene (TS2: Δ*G* = 43.4 kcal mol^–1^; TS4: Δ*G* = 82.2 kcal mol^–1^) participating in the proton transfer step. In addition, calculations on the two tautomerization steps for the deuterated congener **1** ^**d**^ reveal no significant difference in energy compared to the protium analog (see Table S3). These aspects suggest once again proton tunneling as a feasible factor for the two transition states and is in line with the determined abnormally large KIE for H vs D. The first step of our calculated mechanism is predicted to be exergonic (−5.0 kcal mol^–1^) with one acetonitrile molecule coordinated to a GaCp* moiety of **2**, forming [(MeCN)­Ni­(GaCp*)_4_MeCN]^2+^ (**I**) (see Figure 3). As the key step promoted by the electrophilic nature of the gallium center, migratory insertion of the acetonitrile moiety into a Ga–Cp* bond produces an imido-like species **II** (Δ*G*_R_ = 7.2 kcal mol^–1^) via TS1 (19.0 kcal mol^–1^). Next, intramolecular proton transfer yields an enamide-like species **III** (Δ*G*_R_ = −2.1 kcal mol^–1^) followed by the coordination of a second acetonitrile moiety to the reactive gallium center to form **IV** (Δ*G*_R_ = 15.3 kcal mol^–1^). **IV** may undergo a nucleophilic attack of the enamide-type ligand to the α-carbon atom of the nitrile, leading to C–C bond formation to produce the β-diketiminate species **V** (Δ*G*_R_ = 5.4 kcal mol^–1^) via TS3 (−7.6 kcal mol^–1^). **IV** and TS3 are remarkable in that the enamide moiety adopts a bridging mode between two gallium centers in both structures. Therefore, the gallium-rich coordination sphere around the nickel center actively participates in the bond activation process. Perturbating the system by *in silico* replacing two of the four GaCp* moieties for isoelectronic carbon monoxide or acetonitrile molecules as spectator ligands results in larger HOMO–LUMO gaps for the calculated intermediates for the first migratory insertion step (**I** → **II**; see Supporting Information, Section 6). Therefore, nitrile dimerization is virtually impossible without exclusively GaCp* moieties coordinated to the nickel center. Together, this demonstrates that the “all-gallium” environment is key to this unique reactivity. Lastly, compound **1** is obtained as the final product (Δ*G*_R_ = −54.1 kcal mol^–1^) after another tautomerization of the β-diketiminate species **V** to **VI** via TS4 and the coordination of a fifth GaCp* moiety. For comparison, mechanistically related cascades of migratory insertion and subsequent tautomerization have been reported also for the addition of strong nucleophiles like phosphorus ylides to nitriles coordinated at Pt­(II) centers.^30–32^

**Figure 3:**
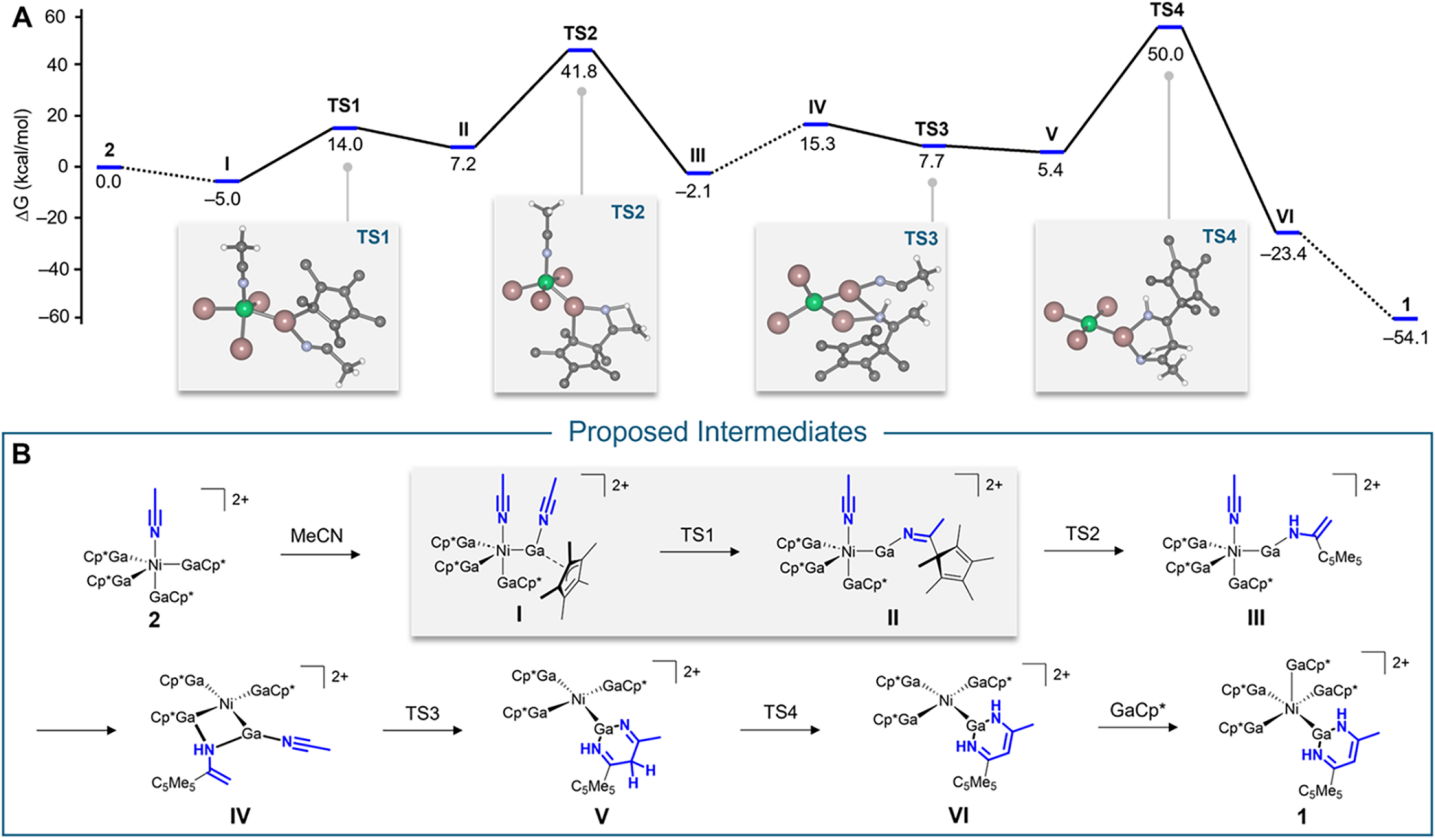
(A) Energy profile for the dimerization of acetonitrile at a gallium center starting from **2** (together with one MeCN and one GaCp* unit) includes all relevant transition states. (B) Proposed intermediates for the dimerization of acetonitrile.

Noteworthy, neither of the individual reagents GaCp* and [Ni­(MeCN)_6_]­(BAr^F^)_2_ nor the neutral complex Ni­(GaCp*)_4_ exhibit reactivity toward acetonitrile under the same conditions like in the synthesis of **1**, even after one week of reaction time (see Figures S1–S3). Consequently, both nickel and gallium centers and the substantial degree of umpolung at the Ga are crucial for the dimerization of acetonitrile, which nicely indicates the cooperation between both metals. The fact that a Cp* ligand is transferred from gallium to the α-carbon atom of acetonitrile points toward an electrophilic polarization of nitrile at the gallium center. This finding appears paradox in that GaCp* usually serves as a “pure” σ-donor (or L-type) ligand in transition metal complexes.^33^ The σ-accepting (or Z-type ligand) properties of GaCp* were calculated to be poor. Instead, σ-donation to TM centers dominates since the vacant p-orbitals of the Ga^+^ cation are partially occupied by the π-orbitals of the Cp* ligand.^34^ Therefore, GaCp* is considered a strong nucleophile instead of a potent electrophile. The coordination to the Lewis acidic Ni^2+^ center, however, seems to alter the electronic structure in such a manner that the gallium atom is more positively polarized and shows an enhanced electrophilic character. This also reflected by probing the Lewis acidity of [(MeCN)­Ni­(GaCp*)_4_]­(BAr^F^)_2_ (**2**) employing the Gutmann–Beckett method using triethylphosphine oxide and 1,2-difluorobenzene as solvents.^35,36^ As **2** generally decomposes readily in solution within several minutes, we obtained multiple peaks in the ^31^P NMR spectrum (see Figure S15), which makes a direct assignment to an adduct of **2** with Et_3_PO virtually impossible. However, all signals are detected in a range from 85.53 to 73.83 ppm and are therefore significantly downfield shifted compared to the free phosphine oxide. This results in a calculated acceptor number between 73 and 98, which indicates strong Lewis acidity for [(MeCN)­Ni­(GaCp*)_4_]­(BAr^F^)_2_ (**2**). Consequently, this leads to an umpolung of the GaCp* moiety. In that way, Cp* as a comparably weak nucleophile can attack the α-carbon atom of acetonitrile, leading to C–C bond formation. As a consequence, reports on electrophilic activation of acetonitrile and nitrile dimerization mainly exist for complexes of highly electrophilic metal centers, such as scandium,^37^ yttrium,^38,39^ titanium,^39–42^ zirconium,^37,42^ and tungsten^43–45^ or actinides such as uranium.^46^ So far, only the reverse scenario—the umpolung of an electrophilic main group metal to a potent nucleophile—has been reported for a beryllocene unit that is coordinated by a nucleophilic, anionic aluminyl ligand.^47^

## Conclusions

To conclude, we have shown that combining an oxidized transition metal with a low-valent main group metal leads to umpolung of the main group metal to a potent Lewis acid. This resembles an unprecedented way to harness the main group metal as the center for bond activation reactions, which is exemplified by the dimerization of acetonitrile. For the future, we anticipate exploring more challenging reactivity patterns akin to this showcase with our system.

## Experimental Section

### General

All experiments were carried out using standard Schlenk and glovebox techniques under an atmosphere of purified argon. Glassware was heated with hexamethyldisilazane to yield passivated surfaces. Solvents were dried using an MBraun Solvent Purification System (SPS) and stored over activated 3 Å molecular sieves. Fluorobenzene (Sigma-Aldrich, 99%), 1,2-difluorobenzene (ABCR Chemicals Germany, 98%) and 3-fluorotoluene (Sigma-Aldrich, 99%) were distilled fractionally, dried by passing through a column of activated neutral alumina, and stored over activated 3 Å molecular sieves in grease-free containers inside a glovebox. The final water content of all solvents was checked by Karl Fischer titration and did not exceed 2 ppm. All solvents were degassed prior to use. Chemicals were bought from commercial suppliers such as Sigma-Aldrich, ABCR Chemicals Germany, and TCI Chemicals (triethylphosphine oxide, >95%). Ni­(acac)_2_ was dried by refluxing the hydrated complex (ABCR Chemicals Germany) in dry toluene using a Dean–Stark apparatus and drying the green residue overnight *in vacuo* at 110 °C after draining the solvent.^48^ The synthesis procedures for Ag­(BAr^F^), Tl­(BAr^F^), [Ni­(MeCN)_6_]­(BAr^F^)_2_ as well as GaCp* and reagents necessary for their preparation (Na­(BAr^F^), H­(Et_2_O)_2_(BAr^F^), and KCp*) are described in the Supporting Information. Cp*H used for the synthesis of KCp* was prepared according to a literature-known procedure.^49^ Deuterated solvents were stored over activated 3 Å molecular sieves and degassed prior to use.

### Analytical Methods

NMR spectra were recorded on either a Bruker AV III 400US or an AVHD 500 spectrometer, as well as a Bruker DRX 400 spectrometer for low-temperature measurements. Spectra were referenced to the residual solvent peak as an internal standard. Spectra in nondeuterated 1,2-difluorobenzene were recorded either using a sealed capillary containing C_6_D_6_ or by adding 10 vol% C_6_D_6_ to the solution. Chemical shifts are reported in parts per million relative to tetrakis­(trimethylsilyl)­silane for ^1^H and ^13^C, as well as BF_3_•Et_2_O for ^11^B, MeNO_2_ for ^15^N, CFCl_3_ for ^19^F and H_3_PO_4_ (85% in water) for ^31^P NMR spectra, including the relative integral, multiplicity (s = singlet, d = doublet, t = triplet, q = quartet, and m = multiplet), and assignment.

UV–Vis spectra were measured either on an Agilent Technologies Cary 60 UV–Vis spectrometer in a quartz cuvette (*d* = 1 cm) under an argon atmosphere or in glass vials inside an argon-filled glovebox using an Ocean Insight FLAME-T-XR1-ES miniature spectrometer, equipped with a DH-2000-BAL UV–Vis–NIR light source and a submersible sensor with a path length of 1 mm. Samples were prepared by diluting the respective compound in 1,2-difluorobenzene and passing the solution through a syringe filter.

FT-IR spectra were measured on a Bruker Alpha FT-IR spectrometer with ATR geometry using a diamond ATR unit in an argon-filled glovebox. The spectra were processed by using the OPUS software package (version 7.5, Bruker Optik GmbH 2014).

High-resolution mass spectra were acquired using a ThermoFisher Scientific Exactive Plus Orbitrap mass spectrometer equipped with a Linden CMS LIFDI (liquid injection field desorption ionization) source. Samples were supplied via a fused silica capillary from a glovebox under an argon atmosphere to enable the measurement of highly air-sensitive compounds.^50^ Spectra were evaluated using FreeStyle 1.3 software (ThermoFisher Scientific). Reference isotope patterns were calculated using *enviPat Web*.^51^

Elemental analyses were conducted at the microanalytical laboratory at the Technical University of Munich.

### Computational Methods

Computational modeling of the molecular structures were performed using the ORCA5.0 software package^52^ with the exchange-correlation functional BP86.^53,54^ Grimme’s Dispersion correction including Becke–Johnson damping (D3BJ)^55,56^ was used. The conductor-like polarizable continuum model (CPCM) was used for 1,2-difluorobenzene. The structure optimization and analytical calculations of the Hessian were performed using Ahlrich’s def2-TZVPP basis set^57^ with the auxiliary basis def2/J.^58^ Optimized geometries of intermediates and transition states were plotted using VESTA 3.^59^

### Synthesis of [Ni­(GaCp*)_4_(GaN_2_C_14_H_21_)]­(BAr^F^)_2_ (**1**)

[Ni­(MeCN)_6_]­(BAr^F^)_2_ (0.5062 g, 247 μmol, 1.00 equiv) is dissolved in 1,2-difluorobenzene (6 mL) and GaCp* (0.2532 g, 1.24 mmol, 5.00 equiv) is added via syringe inside a glovebox, resulting in an intensively violet solution. After stirring overnight at room temperature for 18 h, the obtained orange solution is concentrated to half of its volume and layered with *n*-hexane (10 mL). After a few days, orange crystals have formed, which are separated via cannula filtration, washed with *n*-hexane (1 mL), and dried *in vacuo*. Compound **1** is obtained as a crystalline orange material (0.5940 g, 206 μmol, 83%). Well-shaped crystals suitable for SC-XRD were obtained by cooling the filtrate to −35 °C for several days. **Caution!** This compound is highly air-sensitive and reacts violently with water. Residues were quenched by careful addition of isopropanol.

^1^H NMR (CD_2_Cl_2_, 298 K, 400 MHz): δ = 7.77–7.71 (m, 16H, BAr^F^), 7.58 (s, 8H, BAr^F^), 7.27 (s, 1H, N_1_ *H*), 7.18 (s, 1H, N_2_ *H*), 5.79–5.75 (m, 1H, nacnac–C*H*), 2.38 (s, 3H, nacnac–C*H*_3_), 1.92 (s, 60H, Cp*–C*H*_3_), 1.89 (s, 6H, nacnac, C*H*_3_), 1.78 (s, 6H, nacnac, C*H*_3_), 1.32 (s, 3H, Me_4_C_4_C–C*H*_3_).

^11^B NMR (CD_2_Cl_2_, 298 K, 128 MHz): δ = −6.57 (s, *B*(Ar)_4_).

^13^C NMR (CD_2_Cl_2_, 298 K, 162 MHz): δ = 178.2 (C_5_Me_5_ *C*=NH), 174.8 (Me*C*=NH), 162.4 (q, *J*_B–C_ = 49.8 Hz, B–*C*), 141.1 (C_5_Me_5_, Me–C=*C*), 140.6 (C_5_Me_5_, Me–C–C=*C*), 135.5 (Ar^F^), 129.6 (qdd, *J*_C–F_ = 31.6, 5.7, 2.8 Hz, Ar^F^), 125.3 (q, *J*_C–F_ = 272.4 Hz, Ar^F^), 118.1 (Ar^F^), 117.0 (Ga–*C*_5_Me_5_), 99.6 (Me-CNH-*C*H), 65.6 (Me*C*–C_4_Me_4_), 28.7 (*Me*C=NH), 19.0 (C_5_Me_5_, Me_4_C_4_C–*C*H_3_), 12.0 (C_5_Me_5_, *C*H_3_), 11.0 (C_5_Me_5_, *C*H_3_), 10.4 (Ga–C_5_ *Me*_5_).

^19^F NMR (CD_2_Cl_2_, 298 K, 376 MHz): δ = −62.79 (s, CF_3_).

IR (ATR, 298 K): *ṽ* [cm^–1^] = 3232 (vw, NH), 2975 (w, CH), 2920 (w, CH), 2867 (w, CH), 1610 (w), 1540 (w), 1455 (w), 1353 (s, CF), 1274 (s, CF), 1120 (s, CF), 886 (m), 839 (m), 744 (w), 713 (m), 681 (m), 670 (m).

Elemental analysis calculated for C_118_H_103_B_2_F_48_Ga_5_N_2_Ni (M = 2899.99 g mol^–1^): C 49.04, H 3.59, N 0.97. Found: C 48.63, H 3.78, N 1.49.

LIFDI MS: *m*/*z* = 2027.2677 (calculated for NiGa_5_C_86_H_93_N_2_BF_24_^+^: *m*/*z* = 2027.2683).

UV–Vis (1,2-difluorobenzene): λ [nm] = 409 (ε = 5192 L mol^–1^ cm^–1^).

### Synthesis of [(MeCN)­Ni­(GaCp*)_4_]­(BAr^F^)_2_ (**2**)

[Ni­(MeCN)_6_]­(BAr^F^)_2_ (0.1021 g, 50 μmol, 1.00 equiv) is suspended in dichloromethane (2 mL) and GaCp* (0.0505 g, 246 μmol, 4.90 equiv) is added while stirring, resulting in an intensively violet colored reaction solution. The walls of the Schlenk tube are rinsed with dichloromethane (1 mL), and the resulting solution is stirred for 30 min at room temperature. The reaction is immediately cooled to −80 °C and *n-*hexane (10 mL) is added to precipitate the product, which is stored at −80 °C overnight to ensure complete precipitation. **2** is obtained as a fine dark violet powder (0.0683 g, 26 μmol, 53%) after cannula filtration, washing with *n-*hexane (2 × 1 mL), and drying *in vacuo* while slowly warming to room temperature. Block-shaped crystals suitable for SC-XRD were obtained by layering a solution of **2** in 1,2-difluorobenzene with *n-*hexane at −35 °C and cooling for several days. **Caution!** This compound is highly air-sensitive and reacts violently with water. Residues were quenched by the careful addition of isopropanol.

^1^H NMR (CD_2_Cl_2_, 268 K, 400 MHz): δ = 7.72 (s, 16H, BAr^F^), 7.56 (s, 8H, BAr^F^), 2.44 (s, 3H, NCC*H*_3_), 2.00 (s, 45H, Cp*–C*H*_3_).

IR (ATR): 2932 (w), 2326 (w), 2299 (w), 1610 (m), 1509 (m), 1554 (s), 1274 (s), 1160 (s), 1111 (s), 838 (m), 797 (m), 713 (m), 682 (m), 669 (m).

Elemental analysis calcd for C_106_H_84_B_2_F_48_Ga_4_NNi (M = 2642.97 g mol^–1^): C 48.17, H 3.20, N 0.53. Found: C 48.38, H 3.42, N 1.07.

UV–Vis (1,2-difluorobenzene): λ (nm) = 533 (ε = 5669 L mol^–1^ cm^–1^).

### Synthesis of (**1**) from (**2**), GaCp*, and MeCN

[(MeCN)­Ni­(GaCp*)_4_]­(BAr^F^)_2_ **2** (0.0644 g, 24 μmol, 1.00 equiv) is dissolved in 1,2-difluorobenzene (1 mL) and GaCp* (0.0055 g, 27 μmol, 1.10 equiv), and dry acetonitrile (0.0054 g, 132 μmol, 5.40 equiv) is added via syringe inside a glovebox. The walls of the Schlenk tube are rinsed with 1,2-difluorobenzene (1 mL). The resulting deeply purple solution is stirred at room temperature overnight while its color changes from bright violet to clear orange over the course of a few hours. After reducing the solution to half of its volume, it is layered with *n-*hexane (4 mL) and crystals of [Ni­(GaCp*)_4_(GaN_2_C_14_H_21_)]­(BAr^F^)_2_ (0.0481 g, 17 μmol, 68%) are obtained after filtration and drying *in vacuo*. The spectral data match those of compound **1**.

### Synthesis of [Ni­(MeCN-*d*_3_)_6_]­(BAr^F^)_2_

Anhydrous nickel­(II) bromide (0.0401 g, 184 μmol, 1.00 equiv) and the respective amount of either Tl­(BAr^F^) (0.4012 g, 376 μmol, 2.05 equiv) or AgBAr^F^ (0.3652 g, 376 μmol, 2.05 equiv) are weighed into a Schlenk tube, and dry deuterated acetonitrile (4 mL) is added. The resulting blue solution is stirred at room temperature overnight. The supernatant pale blue solution is decanted from AgBr as a fine beige precipitate. After washing the precipitate with deuterated acetonitrile (0.5 mL), the resulting solution is passed through a syringe filter (43 μm) inside a glovebox and reduced *in vacuo* to approximately 2 mL. Storing at −32 °C overnight yields violet needle-shaped crystals of [Ni­(MeCN-*d*_3_)_6_]­(BAr^F^)_2_ (0.2631 g, 127 μmol, 69%) after cannula filtration and drying *in vacuo*. **Caution!** Nickel salts and their dust are known (or at least suspected) to be carcinogenic. [Ni­(MeCN*-d*_3_)_6_]­(BAr^F^)_2_ was handled only inside a glovebox equipped with appropriate dust filters.

IR (ATR, 298 K): *ṽ* [cm^–1^] = 2318 (w), 1611 (w), 1353 (s), 1275 (s), 1168 (m), 1110 (s), 888 (m), 838 (m), 712 (m), 672 (m), 669 (m).

Elemental analysis calculated for NiC_76_H_24_D_18_B_2_F_48_N_6_ (M = 2049.59 g mol^–1^): C: 44.54, H (and D): 2.95, N: 4.10; found: C: 44.35, H: 1.88, N: 4.10.

### Synthesis of [Ni­(GaCp*)_4_(GaN_2_C_14_H_15_D_6_)]­(BAr^F^)_2_ (**1** ^**d**^)

[Ni­(MeCN-*d*_3_)_6_]­(BAr^F^)_2_ (0.0993 g, 48 μmol, 1 equiv) is dissolved in 1,2-difluorobenzene (2 mL), and GaCp* (0.0526 g, 257 μmol, 5.30 equiv) is added via syringe inside a glovebox, resulting in an intensively violet solution. Stirring at room temperature for 3 weeks yields a dark orange solution, which is layered with *n*-hexane (6 mL). After 3 days, orange crystals have formed, which are separated via cannula filtration, washed with *n*-hexane (1 mL), and dried *in vacuo*. Compound **1** ^**d**^ is obtained as a crystalline orange material (0.0924 g, 32 μmol, 66%). *Caution!* This compound is—analogously to the nondeuterated congener **1**—highly air-sensitive and reacts violently with water. Residues were quenched by careful addition of isopropanol.

^1^H NMR (CD_2_Cl_2_, 298 K, 400 MHz): δ = 7.77–7.71 (m, 16H, BAr^F^), 7.56 (s, 8H, BAr^F^), 1.92 (s, 60H, Cp*–C*H*_3_), 1.90 (s, 6H, nacnac, C*H*_3_), 1.77 (s, 6H, nacnac, C*H*_3_), 1.32 (s, 3H, Me_4_C_4_C–C*H*_3_).

LIFDI MS: *m*/*z* = 2033.3030 (calculated for NiGa_5_C_86_H_93_N_2_BF_24_^+^: *m*/*z* = 2033.3059).

## Supplementary Material





## Notes

Additional experimental procedures, crystallographic data, NMR, IR, UV–Vis, and LIFDI MS data for all compounds and computational details. (PDF)
Cartesian coordinates. (XYZ)
