# Supplementary material for: Cooperative Reactivity Induced by All-Gallium Coordination at Nickel
Source: Inorg Chem. 2025 Jul 18;64(30):15707–14. doi: 10.1021/acs.inorgchem.5c02296 (PMC12326349; doi:10.1021/acs.inorgchem.5c02296)
Supplement: Supplementary file 1 [file ic5c02296_si_001.pdf]

Supporting Information

Cooperative Reactivity Induced by All-Gallium Coordination at Nickel

*Johannes Stephan [a,b], Raphael Bühler [a,b], Fabrizio E. Napoli [a,b], Christian Gemel [a,b] & Roland A. Fischer [a,b]**

[a]Technical University of Munich, TUM School of Natural Sciences, Department of Chemistry, Chair of Inorganic and Metal-Organic Chemistry, Lichtenbergstraße 4, D-85748 Garching, Germany

[b]Catalysis Research Center, Technical University of Munich, Ernst-Otto-Fischer-Straße 1, D-85748 Garching, Germany

Correspondence to: roland.fischer@tum.de

TABLE OF CONTENTS

1. Experimental Procedures ... 2
NaBAr^F ... 2
H(Et$_2$O)$_2$(BAr^F) (Brookhart's acid) ... 3
[Ni(MeCN)$_6$](BAr^F)$_2$ ... 3
Ag(BAr^F) ... 4
Tl(BAr^F) ... 4
KCp* ... 5
GaCp* ... 6
Determination of the kinetic isotope effect (KIE) ... 6
Determination of acceptor numbers (AN) using the Gutmann-Beckett method ... 7
2. Crystallography ... 8
General ... 8
[Ni(GaCp*)$_5$(MeCN)$_2$](BAr^F)$_2$ (**1**) ... 9
[Ni(GaCp*)$_4$MeCN](BAr^F)$_2$ (**2**) ... 11
[Ni(MeCN)$_6$](BAr^F)$_2$ ... 13
3. NMR Spectra ... 15
Stability study: GaCp* in the presence of acetonitrile ... 15
Additional NMR Spectra of **1**, **1^d**, **2** and reactants ... 17
4. LIFDI MS & IR Spectroscopic Data ... 32
5. UV-Vis Spectra ... 36
6. DFT Calculations ... 41
Details on the calculated mechanism & optimized geometries ... 41
In silico replacement of GaCp* for carbon monoxide and acetonitrile ... 44
7. References ... 47

1. Experimental Procedures

NaBAr^F

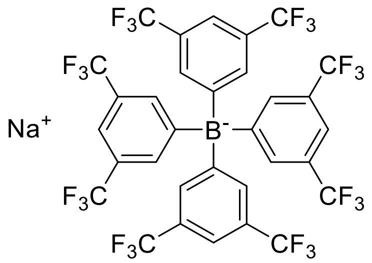

The synthesis procedure for $NaBAr^F$ is based on three literature known protocols.[1-3] Magnesium turnings (1.5810 g, 65.05 mmol, 6.50 eq.) and $NaBF_4$ (1.0994 g, 10.00 mmol, 1.00 eq.), which was dried overnight at 60 °C *in vacuo*, are suspended in dry diethylether (100 mL). To the stirred suspension a solution of 3,5-bis(trifluoromethyl)bromobenzene (9.70 mL, 16.48 g, 56.25 mmol, 5.62 eq.) in 150 mL dry diethylether is added dropwise under gentle reflux. The resulting brownish mixture is heated under reflux for two hours and stirred overnight at room temperature. The obtained light brown suspension is poured gently into a solution of 26.5 g Na_2CO_3 in 200 mL distilled water and stirred for one hour. After removing the $MgCO_3$ precipitate by filtration, the aqueous phase is extracted with Et_2O (3 × 50 mL) and the combined organic phases are dried over Na_2SO_4 and decolorized with activated charcoal. After filtration and removal of the solvent, the crude product is dried *in vacuo* at 100 °C for ten minutes, crushed in a mortar and washed with a cold (–32 °C) mixture of DCM/hexane (2:1) (3 × 25 mL) to remove colored impurities. The product is dried overnight *in vacuo* at 110 °C and subsequently washed with dry toluene (3 × 20 mL) and dry hexane (20 mL). $NaBAr^F$ (7.5633 g, 8.53 mmol, 85%) is obtained as an ivory-colored powder after drying *in vacuo*.

^{1}H NMR (Acetone-d_6, 298 K, 400 MHz): δ = 7.85 – 7.79 (m, 8H, *ortho*-H), 7.70 (s, 4H, *para*-H).

^{11}B NMR (Acetone-d_6, 298 K, 128 MHz): δ = – 6.66 (s, *B*(Ar)$_4$).

^{13}C NMR (Acetone-d_6, 298 K, 101 MHz): δ = 162.5 (q, $^1J_{B\text{-}C}$ = 49.9 Hz, B-*C*), 136.5 (s), 130.0 (qdd, $^1J_{C\text{-}F}$ = 31.7 Hz), 125.3 (q, $^1J_{C\text{-}F}$ = 271.8 Hz), 118.4 (p, $^1J_{C\text{-}F}$ = 3.9 Hz).

^{19}F NMR (Acetone-d_6, 298 K, 376 MHz): δ = –63.28 (s, CF_3).

Caution! The reaction was found to react violently if tetrahydrofuran is used as a solvent. Reports on the exothermic decomposition of fluorinated Grignard reagents exist, including the herein used 3,5-bis(trifluoromethyl)phenylmagnesium bromide.[3, 4] Therefore, it is imperative to use diethylether as a solvent.

H(Et$_2$O)$_2$(BAr^F) (Brookhart's acid)

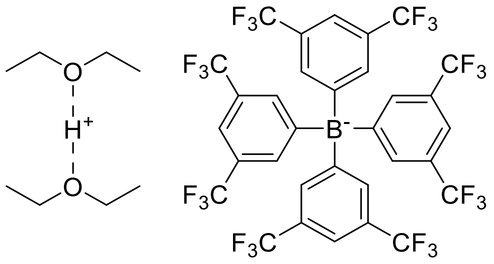

The synthesis of Brookhart's acid is based on two literature known protocols.[5, 6] NaBAr^F (5.0002 g, 5.64 mmol, 1.00 eq.) is dissolved in dry diethylether (20 mL) and cooled to 0 °C. Subsequently, a 2 M solution of HCl (3.40 mL, 6.80 mmol, 1.21 eq.) in Et$_2$O is added. The resulting turbid mixture is stirred at 0 °C for ten minutes after which it is filtered over a P4 inert gas frit. After washing the filter cake with Et$_2$O (2 × 2 mL), the filtrate is cooled to –80 °C for half an hour. Dry hexane (10 mL) is added to precipitate the product completely and the supernatant solution is filtered off *via* cannula filtration at –80 °C. The crude product is dried *in vacuo* while slowly warming up to room temperature. H(Et$_2$O)$_2$(BAr^F) (4.9811 g, 4.92 mmol, 87%) is obtained as a slightly beige, microcrystalline powder.

^{1}H NMR (CD$_3$CN, 298 K, 400 MHz): δ = 11.42 (bs, 1H, *H*(Et$_2$O)$_2$), 7.74 – 7.68 (m, 8H, *ortho*-H), 7.67 (s, 4H, *para*-H), 3.61 (q, ^1J = 7.1 Hz, 8H, CH$_2$), 1.18 (t, ^1J = 7.1 Hz, 12H, CH$_3$).

^{11}B NMR (CD$_3$CN, 298 K, 128 MHz): δ = –6.70 (s, *B*(Ar)$_4$).

^{13}C NMR (CD$_3$CN, 298 K, 101 MHz): δ = 162.6 (q, $^1J_{B\text{-}C}$ = 49.8 Hz, B-*C*), 136.4 (s), 129.9 (qdd, $J_{C\text{-}F}$ = 31.6, 5.7, 2.8 Hz), 125.5 (q, $^1J_{C\text{-}F}$ = 271.7 Hz), 118.7 (p, $J_{C\text{-}F}$ = 3.9 Hz), 67.6 (s, *C*H$_2$), 15.0 (s, *C*H$_3$).

^{19}F NMR (CD$_3$CN, 298 K, 376 MHz): δ = –63.30 (s, CF$_3$).

Caution! Corrosive gas HCl gas is used for this preparation. The entire apparatus needs to be set-up in a well-ventilated fume hood.

[Ni(MeCN)$_6$](BAr^F)$_2$

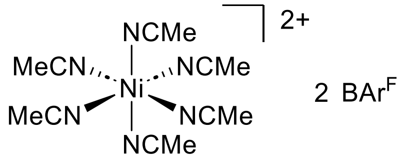

The synthesis procedure for [Ni(MeCN)$_6$](BAr^F)$_2$ is based on a literature known protocol for preparing [Ni(MeCN)$_6$](BF$_4$)$_2$ and [Ni(MeCN)$_6$](SO$_3$CF$_3$)$_2$.[7] Anhydrous bis(acetylacetonato)-nickel(**II**) (0.1001 g, 389 µmol, 1.00 eq.) and H(Et$_2$O)$_2$BAr^F (0.7892 g, 778 µmol, 2.00 eq.) are weighed into a Schlenk tube and acetonitrile (8 mL) is added. The resulting blue solution is stirred at room temperature overnight. After reducing the solution *in vacuo* to approximately 3 mL and storing at –32 °C overnight, violet crystals of [Ni(MeCN)$_6$](BAr^F)$_2$ (0.6985 g, 344 µmol, 88%) are obtained after cannula filtration and drying *in vacuo*.

^{1}H NMR (1,2-DFB/C_6D_6, 298 K, 400 MHz): δ = 8.60 (m, 16H, *ortho*-H), 7.99 (s, 8H, *para*-H), –32.14 (bs, NCCH_3).

IR (ATR, 298 K): $\tilde{\nu}$ [cm^{-1}] = 2326 (w), 2299 (w), 1610 (w), 1353 (s), 1275 (s), 1168 (m), 1110 (s), 888 (m), 838 (m), 712 (m), 672 (m), 669 (m).

Elemental analysis [%]: Calculated for $NiC_{76}H_{42}B_2F_{48}N_6$: C: 44.94, H: 2.08, N: 4.14, B: 1.06, F: 44.89, Ni: 2.89; found: C: 44.85, H: 1.87, N: 4.07, F: 45.40.

Caution! Nickel salts and their dusts are known (or at least suspected) to be carcinogenic. $[Ni(MeCN)_6](BAr^F)_2$ was only handled inside a glovebox equipped with appropriate dust filters.

Ag(BAr^F)

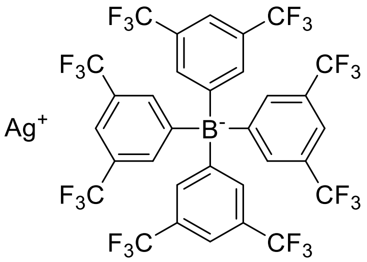

The synthesis procedure for Ag(BAr^F) is based on a literature known protocol.[2] NaBAr^F (1.0499 g, 1.18 mmol, 1.00 eq.) is dissolved in diethylether (20 mL) in a separatory funnel wrapped in aluminum foil under ambient conditions. A solution of $AgNO_3$ (0.3841 g, 2.26 mmol, 1.91 eq.) in 10 mL water is added and the biphasic system is vigorously shaken for at least ten minutes. The organic phase is separated and dried over sodium sulphate with minimum light exposure. The solvent is evaporated in a brown glass Schlenk tube yielding Ag(BAr^F) (0.6941 mg, 0.71 mmol, 60%) as a white powder that was used without further characterization.

Caution! Silver salts are known to be severely irritant and may cause skin burns upon contact.

Tl(BAr^F)

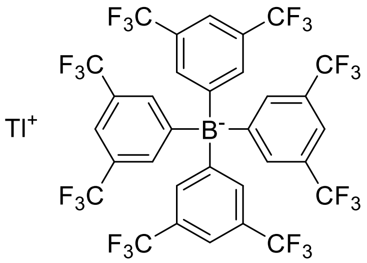

The synthesis procedure is based on a literature known protocol for $Tl[B(C_6F_5)_4]$.[8] $H(Et_2O)_2BAr^F$ (1.0032 g, 0.98 mmol, 1.00 eq.) is weighed into a Schlenk tube inside a glovebox and dissolved in dry diethylether (5 mL) at a Schenk line. TlOEt (0.2604 g, 1.04 mmol, 1.06 eq.) is added *via* a syringe and a slightly turbid mixture forms, most probably due to formation of TlCl from NaCl traces in $H(Et_2O)_2BAr^F$.

The resulting reaction mixture is stirred for ten minutes at room temperature, after which the solvent is removed *in vacuo* to obtain a sticky beige solid. The crude product is dissolved in fluorobenzene (5 mL) and layered with *n*-hexane (10 mL). After three days at room temperature, large crystals of solvent-free Tl(BAr^F) (0.9123 g, 0.85 mmol, 87%) are obtained.

^{1}H NMR (DMSO-*d*$_6$, 298 K, 400 MHz): *δ* = 7.69 (s, 4H, *para*-H), 7.62 (m, 8H, *ortho*-H).

Elemental analysis [%]: Calculated for $TlC_{32}H_{12}BF_{24}$: C: 36.00, H: 1.13, B: 1.01, F: 42.71, Tl: 19.14; found: C: 35.70, H: 1.09.

Caution! **Extreme toxicity hazard!** Thallium salts and their dusts are known to be highly toxic, including inhalation or skin contact as exposure pathways.[9] Numerous cases of fatal poisonings have been reported. Tl(BArF) was only handled in a well-working fume hood using respirator masks or inside a glovebox equipped with appropriate dust filters.

KCp*

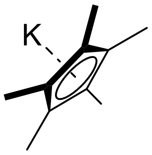

Mineral-oil free potassium hydride (4.0778 g, 101.7 mmol, 1.00 eq.) is suspended in dry tetrahydrofuran (200 mL) in a Schlenk flask. Cp*H (17.1216 g, 125.7 mmol, 1.24 eq.), which has been dried by passing over activated neutral alumina (activity stage Brockmann I), is added dropwise *via* syringe while vigorously stirring. Instantaneously, hydrogen evolution and a fine white precipitate are observed. The suspension is stirred for three days at room temperature with a pressure relief valve attached to the flask. The product is allowed to settle and the supernatant, yellowish solution is filtered off *via* cannula filtration. After washing with dry *n*-hexane (2 × 80 mL), drying for one hour at room temperature and drying overnight at 70 °C (3 × 10^{-3} mbar) to remove tetrahydrofuran traces, KCp* is obtained as a white, free-flowing powder (16.4024 g, 94.1 mmol, 93%).

^{1}H NMR (DMSO-*d*$_6$, 298 K, 400 MHz) δ = 1.85 (s, 15H, C$_5$*Me*$_5$).

^{13}C NMR (DMSO-*d*$_6$, 298 K, 101 MHz) δ = 103.80 (s, *C*$_5$Me$_5$), 12.46 (s, C$_5$*Me*$_5$).

Caution! Hydrogen is classified as a GHS Flammable Gas, Category 1. Working in a well-working fume hood is imperative. The product KCp* is highly pyrophoric; leftovers were quenched by slow addition of isopropanol.

GaCp*

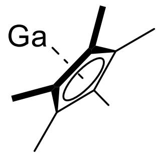

The synthesis of GaCp* is based on literature known protocol by *Jutzi* et al.[10] In the first step, “GaI” is prepared sonochemically.[11] Freshly molten gallium beads (3.80 g, 54.5 mmol, 1.00 eq.) are added to a solution of elemental iodine (6.92 g, 27.3 mmol, 0.50 eq.) in dry benzene (100 mL) in a Schlenk tube. The reaction mixture is sonicated using a *Branson* W-450 D Sonifier operating at an amplitude of 70% with a sequence of two seconds pulse and one second pause for a total sonication time of one hour. The mixture is cooled with an ice or a water bath to prevent overheating the reaction. During sonication, the color of the suspension changes from initially violet to white to grayish-green. Subsequently, KCp* (9.50 g, 54.5 mmol, 1.00 eq.) and a stirring bar are added to the obtained “GaI” inside a glovebox. The suspension is vigorously stirred at room temperature for four days, which leads to a metallic grey precipitate and a yellowish to orange supernatant solution. The precipitate is allowed to settle for at least one hour after which the liquid is filtered *via* cannula and the solid washed with dry *n*-pentane (2 × 50 mL). After reducing *in vacuo*, the oily residue is purified at 80 °C (3 × 10^{-3} mbar) *via* bulb-to-bulb distillation, which yields GaCp* as a yellow oil (5.85 g, 28.5 mmol, 52%) that solidifies upon cooling in a refrigerator.

^{1}H NMR (C_6D_6, 298 K, 400 MHz) δ = 1.94 (s, 15H, $C_5\mathit{Me}_5$).

^{13}C NMR (C_6D_6, 298 K, 101 MHz) δ = 113.53 (s, C_5Me_5), 9.93 (s, $C_5\mathit{Me}_5$).

Caution! This procedure requires the use of a strong ultrasonication device that may cause mechanical damage if touched during operation. Benzene as a carcinogenic agent constitutes a significant safety hazard and must be handled with extreme care and only in a well-working fume hood. Using toluene as a substitute leads to benzyl iodide as a side product.[10] KCp* and the product GaCp* are highly pyrophoric substances; leftovers were quenched by slow addition of isopropanol while vigorously stirring.

Determination of the kinetic isotope effect (KIE)

The reaction progress for the formation of **1** can be easily monitored by the disappearance of the intense violet color of the intermediate **2**. Once the solution has changed its color to a clear and bright orange (indicating the presence of exclusively **1**), the reaction was deemed finished for both **1** and **1^{d}**. For the hydrogen congener, the reaction proceeded overnight (18 h) and for the deuterium congener within three weeks. This leads a determined KIE of 28:

$$KIE = \frac{21\ days}{18\ h} = \frac{504\ h}{18\ h} = 28$$

Determination of acceptor numbers (AN) using the Gutmann-Beckett method

We determined the Lewis acidity and the acceptor number (AN), respectively, of $[(MeCN)Ni(GaCp^*)_4](BAr^F)_2$ (**2**) by employing the Gutmann-Beckett method using triethylphosphine oxide.[12, 13] To **2** (15 mg, 3 µmol, 1.00 eq.) in 1,2-difluorobenzene was added a stock solution of Et_3PO (5 mg/mL) in 1,2-difluorobenzene (120 µL, 0.6 mg, 0.79 eq.). As the ^{31}P NMR (see Figure S15) shows multiple peaks due to thermal decomposition of **2**, the acceptor number was calculated for a range with the peak at 85.53 ppm as the most shifted and 73.83 ppm as the least shifted peak according to following equation:

$$AN_{max} = 2{,}21 \times (\delta(ppm) - 41.00) = 2{,}21 \times (85.53 - 41.00) \approx 98$$

$$AN_{min} = 2{,}21 \times (\delta(ppm) - 41.00) = 2{,}21 \times (73.83 - 41.00) \approx 73$$

Accordingly, we also determined the acceptor number of 1,2-difluorobenzene, which leads to chemical shift of 47.41 ppm (see Figure S16). This corresponds to an acceptor number of 14:

$$AN_{o-DFB} = 2{,}21 \times (\delta(ppm) - 41.00) = 2{,}21 \times (47.41 - 41.00) \approx 14$$

2. Crystallography

General

Data were collected on a single crystal x-ray diffractometer equipped either with a CMOS detector (Bruker APEX IV, κ-CMOS) and a TXS rotating anode with MoK_α radiation (λ = 0.71073 Å) and a Helios optic or equipped with a CPAD detector (Bruker Photon II) for compounds **1** and $[Ni(MeCN)_6](BAr^F)_2$ and an IMS microsource with MoK_α radiation (λ = 0.71073 Å) and a Helios optic for compound **2**, using the APEX4 software package.[C1] Measurements were performed on single crystals coated with perfluorinated ether. The crystals were fixed on top of a Kapton micro sampler and frozen under a stream of cold nitrogen. A matrix scan was used to determine the initial lattice parameters. Reflections were corrected for Lorentz and polarisation effects, scan speed, and background using SAINT.[C2] Absorption correction, including odd and even ordered spherical harmonics was performed using SADABS.[C3] Space group assignments were based upon systematic absences, E statistics, and successful refinement of the structures. The structures were solved using SHELXT with the aid of successive difference Fourier maps, and were refined against all data using SHELXL-2019/1 in conjunction with SHELXLE.[C4,C5,C6] Hydrogen atoms were calculated in ideal positions as follows: Methyl hydrogen atoms were refined as part of rigid rotating groups, with a C–H distance of 0.98 Å and $U_{iso(H)} = 1.5 \cdot U_{eq(C)}$. Other H atoms were placed in calculated positions and refined using a riding model, with methylene and aromatic C–H distances of 0.99 Å and 0.95 Å, respectively, other C–H distances of 1.00 Å, all with $U_{iso(H)} = 1.2 \cdot U_{eq(C)}$. Non-hydrogen atoms were refined with anisotropic displacement parameters. Full-matrix least-squares refinements were carried out by minimizing $\Sigma\, w(F_o^2 - F_c^2)^2$ with the SHELXL weighting scheme.[C5] Neutral atom scattering factors for all atoms and anomalous dispersion corrections for the non-hydrogen atoms were taken from *International Tables for Crystallography*.[C7] A split layer refinement was used for disordered groups and additional restraints on distances, angles and anisotropic displacement parameters were employed to ensure convergence within chemically reasonable limits, if necessary. Whole molecule disorder and rotational disorder, e.g. of CF_3 moieties, was modelled using the *DSR* tool plugin within SHELXLE.[C8] For compound **1**, one heavily disordered molecule of *n*-hexane was treated as a diffuse contribution to the overall scattering without specific atom positions using the PLATON/SQUEEZE procedure.[C9] Images of the crystal structures were generated with PLATON (SI) and Mercury (main article).[C10, C11] CIF files and tables were generated using FinalCif.[C12]

[Ni(GaCp*)$_5$(MeCN)$_2$](BAr^F)$_2$ (1)

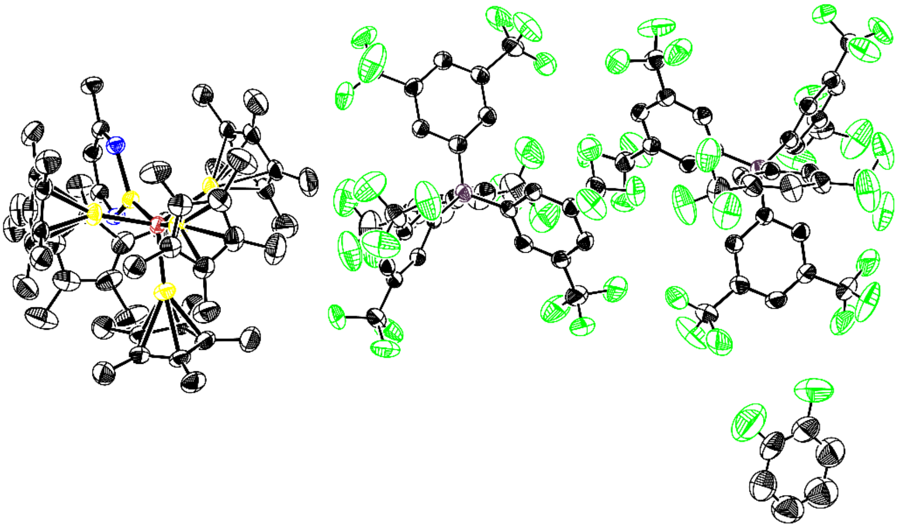

Deposition number (CCDC)	2429679	
Chemical formula	$C_{136}H_{127}B_2F_{52}Ga_5N_2Ni$	
Formula weight	3206.32	
Temperature	100(2) K	
Wavelength	0.71073 Å	
Crystal size	0.098 x 0.127 x 0.207 mm	
Crystal system	triclinic	
Space group	P -1	
Unit cell dimensions	a = 14.954(4) Å<br>b = 15.115(4) Å<br>c = 31.972(8) Å	α = 86.988(9)°<br>β = 77.697(8)°<br>γ = 86.723(8)°
Volume	7043.(3) Å^3	
Z	2	
Density (calculated)	1.512 g/cm^3	
Absorption coefficient	1.191 mm^{-1}	
F(000)	3236	
Diffractometer	Bruker D8 Venture	
Radiation source	TXS rotating anode, Mo	
Theta range for data collection	1.92 to 25.03°	
Index ranges	-17<=h<=17, -17<=k<=17, -38<=l<=38	
Reflections collected	405252	
Independent reflections	24834 [R(int) = 0.1102]	
Coverage of independent reflections	99.9%	
Absorption correction	Multi-Scan	
Max. and min. transmission	0.7450 and 0.6370	
Structure solution technique	iterative	
Structure solution program	XT, VERSION 2018/2	
Refinement method	Full-matrix least-squares on F^2	
Refinement program	SHELXL-2019/1 (Sheldrick, 2019)	

Function minimized	$\Sigma\ w(F_o^2 - F_c^2)^2$
Data / restraints / parameters	24834 / 3183 / 2408
Goodness-of-fit on F^2	1.041
Δ/σ_{max}	0.002
Final R indices	19186 data; I>2σ(I) R1 = 0.0580, wR2 = 0.1537<br>all data R1 = 0.0767, wR2 = 0.1679
Weighting scheme	$W=1/[\Sigma^2(F_O^2)+(0.0834P)^2+13.9406P]$<br>where $P=(F_O^2+2F_C^2)/3$
Largest diff. peak and hole	1.114 and -0.992 eÅ^{-3}
R.M.S. deviation from mean	0.086 eÅ^{-3}

$[Ni(GaCp^*)_4MeCN](BAr^F)_2$ (2)

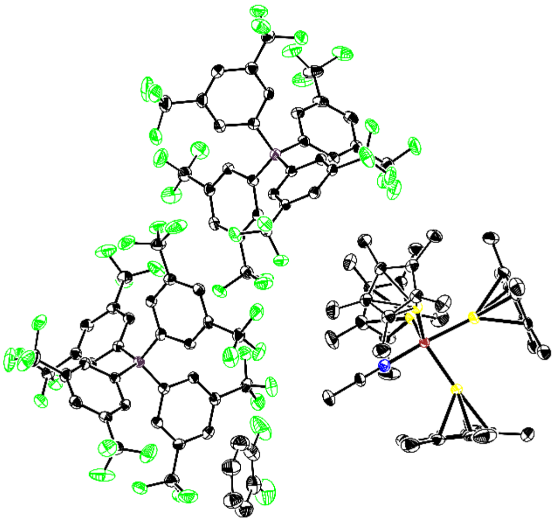

Deposition number (CCDC)	2429680	
Chemical formula	$C_{112}H_{91}B_2F_{50}Ga_4NNi$	
Formula weight	2760.06	
Temperature	100(2) K	
Wavelength	0.71073 Å	
Crystal size	0.091 x 0.122 x 0.153 mm	
Crystal system	monoclinic	
Space group	P 1 21/n 1	
Unit cell dimensions	a = 18.5457(14) Å	α = 90°
	b = 24.970(2) Å	β = 90.420(3)°
	c = 24.6272(19) Å	γ = 90°
Volume	11404.2(15) $Å^3$	
Z	4	
Density (calculated)	1.608 g/cm^3	
Absorption coefficient	1.225 mm^{-1}	
F(000)	5528	
Diffractometer	Bruker D8 Venture Duo IMS	
Radiation source	IMS microsource, Mo	
Theta range for data collection	1.83 to 26.40°	
Index ranges	-23<=h<=23, -31<=k<=31, -30<=l<=30	
Reflections collected	309258	
Independent reflections	23351 [R(int) = 0.0586]	
Coverage of independent reflections	99.8%	
Absorption correction	Multi-Scan	
Max. and min. transmission	0.6996 and 0.7454	
Structure solution technique	iterative	
Structure solution program	SHELXT 2018/2 (Sheldrick, 2018)	
Refinement method	Full-matrix least-squares on F^2	
Refinement program	SHELXL-2019/1 (Sheldrick, 2019)	

Function minimized	Σ w(F_o^2 - F_c^2)^2
Data / restraints / parameters	23351 / 1450 / 1865
Goodness-of-fit on F^2	1.237
Δ/σ_{max}	0.026
Final R indices	18002 data; I>2σ(I) R1 = 0.0463, wR2 = 0.1075<br>all data R1 = 0.0761, wR2 = 0.1337
Weighting scheme	W=1/[Σ^2(F_O^2)+(0.0364P)^2+32.6229P]<br>where P=(F_O^2+2F_C^2)/3
Extinction coefficient	0.0002(0)
Largest diff. peak and hole	0.690 and -0.727 eÅ^{-3}
R.M.S. deviation from mean	0.097 eÅ^{-3}

[Ni(MeCN)$_6$](BAr^F)$_2$

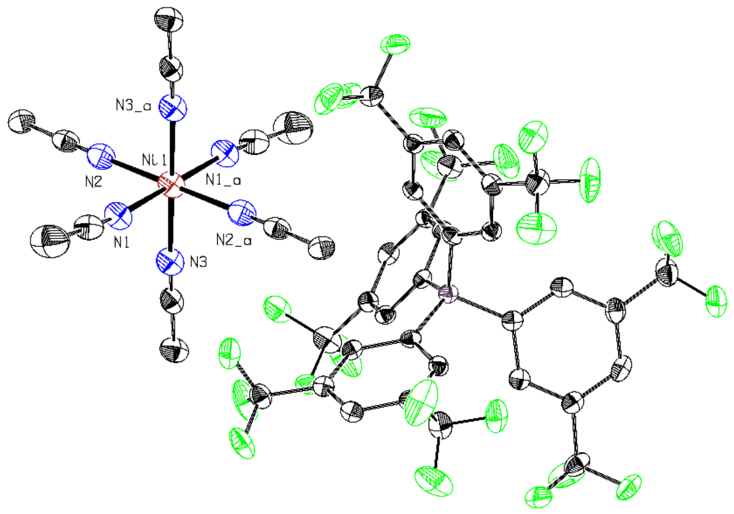

Deposition number (CCDC)	2429681	
Chemical formula	$C_{76}H_{42}B_2F_{48}N_6Ni$	
Formula weight	2031.48	
Temperature	100(2) K	
Wavelength	0.71073 Å	
Crystal size	0.080 x 0.118 x 0.150 mm	
Crystal system	monoclinic	
Space group	P 1 21/n 1	
Unit cell dimensions	a = 15.5145(8) Å	α = 90°
	b = 16.2457(7) Å	β = 103.637(2)°
	c = 16.9220(8) Å	γ = 90°
Volume	4144.8(3) Å^3	
Z	2	
Density (calculated)	1.628 g/cm^3	
Absorption coefficient	0.390 mm^{-1}	
F(000)	2020	
Diffractometer	Bruker D8 Venture	
Radiation source	TXS rotating anode, Mo	
Theta range for data collection	1.84 to 25.03°	
Index ranges	-18<=h<=18, -19<=k<=19, -20<=l<=20	
Reflections collected	60706	
Independent reflections	7313 [R(int) = 0.1248]	
Coverage of independent reflections	99.9%	
Absorption correction	Multi-Scan	
Max. and min. transmission	0.7450 and 0.6450	
Structure solution technique	iterative	
Structure solution program	SHELXT 2018/2 (Sheldrick, 2018)	
Refinement method	Full-matrix least-squares on F^2	
Refinement program	SHELXL-2019/1 (Sheldrick, 2019)	

Function minimized	Σ w(F_o^2 - F_c^2)^2
Data / restraints / parameters	7313 / 564 / 697
Goodness-of-fit on F^2	1.020
Δ/σ_{max}	0.001
Final R indices	5402 data; I>2σ(I) R1 = 0.0483, wR2 = 0.1039<br>all data R1 = 0.0729, wR2 = 0.1151
Weighting scheme	$W=1/[\Sigma^2(F_O^2)+(0.0298P)^2+4.4406P]$<br>where $P=(F_O^2+2F_C^2)/3$
Largest diff. peak and hole	0.531 and -0.600 eÅ^{-3}
R.M.S. deviation from mean	0.060 eÅ^{-3}

3. NMR Spectra

Stability study: GaCp* in the presence of acetonitrile

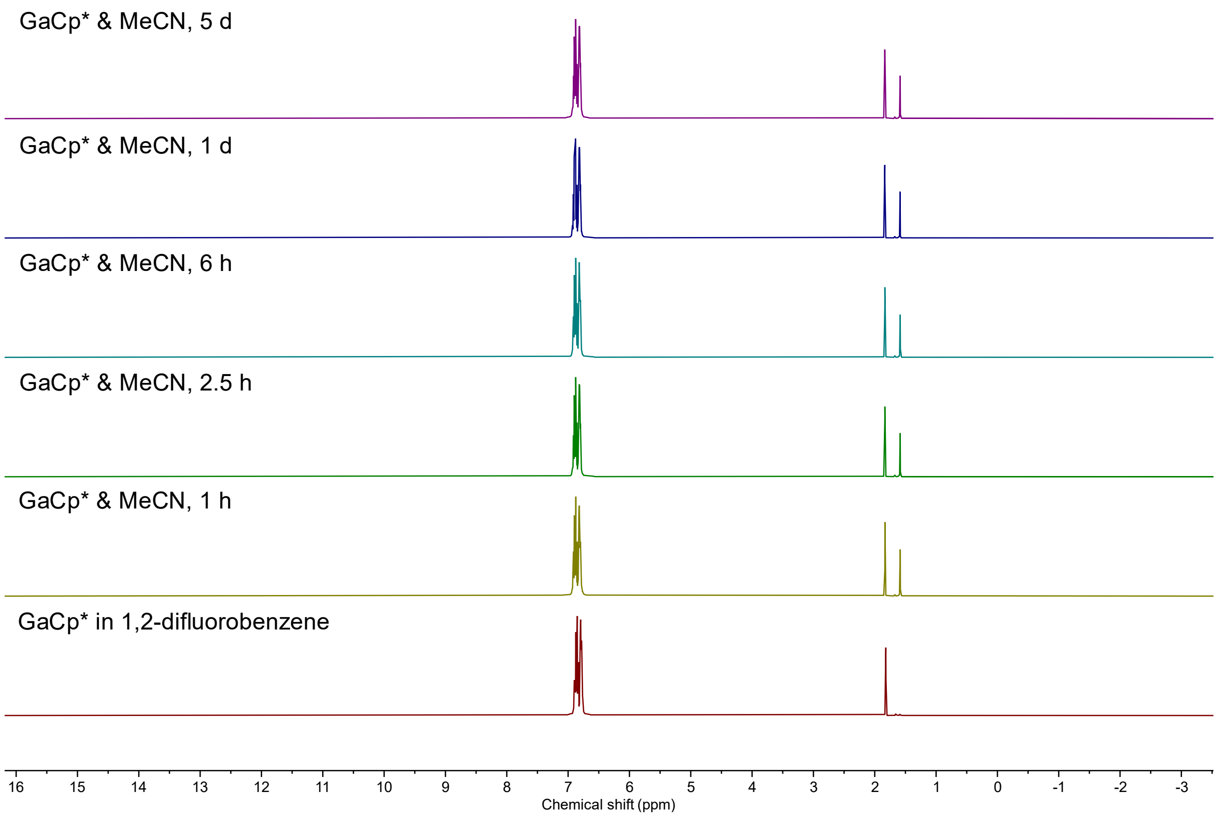

Figure S1: Stacked ^{1}H NMR spectra of GaCp* (10 mg, 4.9 µmol) in 0.5 mL 1,2-difluorobenzene in the presence of excess acetonitrile (10 mg, 24.4 µmol, 5.0 eq.). No conversion of acetonitrile to di- or trimerization products was observed after five days. Consequently, GaCp* showed no reactivity towards MeCN under these conditions.

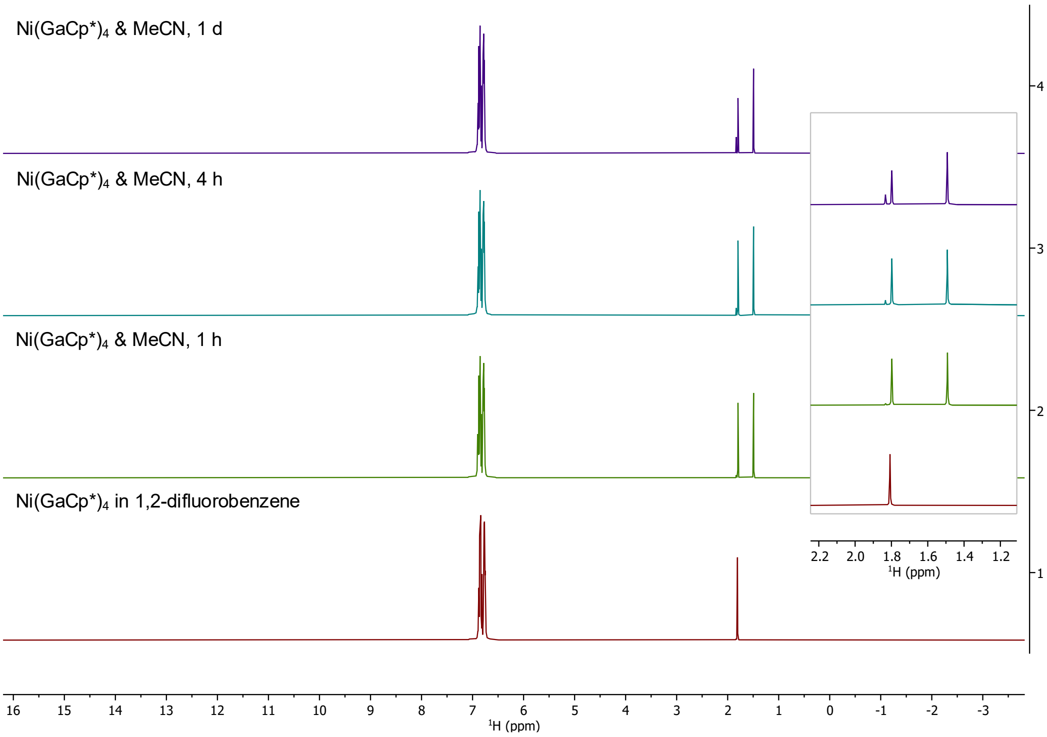

Figure S2: Stacked ^{1}H NMR spectra of $Ni(GaCp^*)_4$ (10 mg) in 0.5 mL 1,2-difluorobenzene in the presence of excess acetonitrile (10 mg, 5.0 eq.). No conversion of acetonitrile to di-, tri- or oligomerization products was observed after five days. However, slight decomposition of the starting material is observed as indicated by the presence of "free" GaCp* (1.83 ppm).

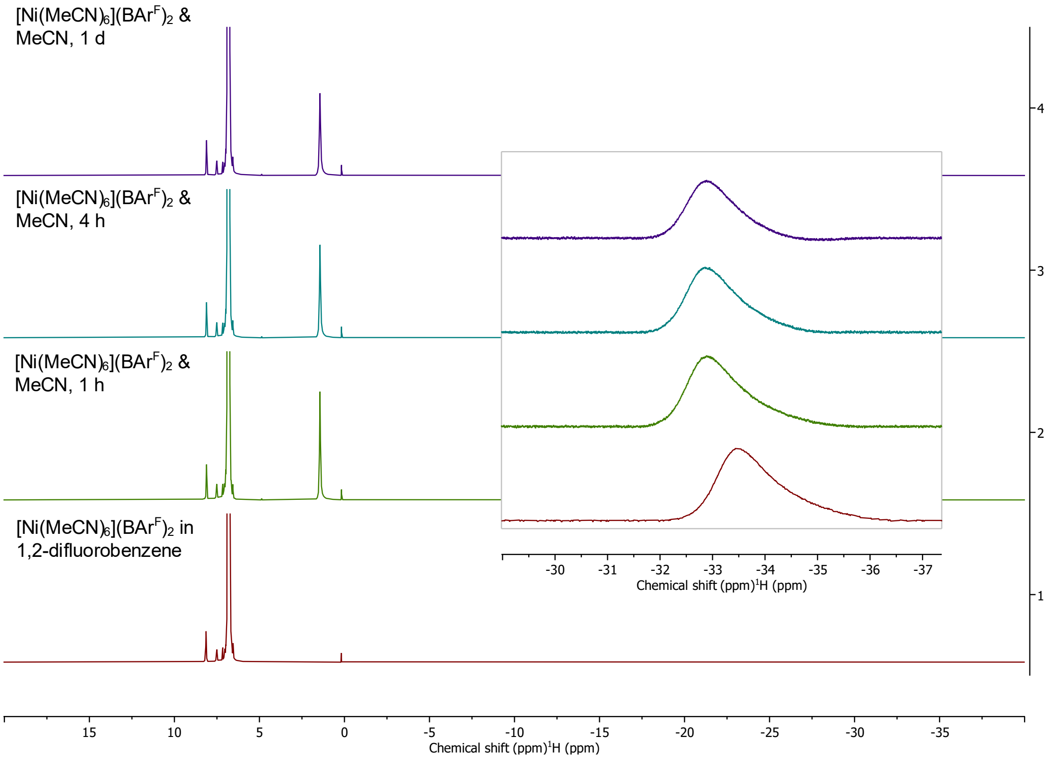

Figure S3: ^{1}H NMR spectra of $[Ni(MeCN)_6](BAr^F)_2$ (20 mg) in 0.5 mL 1,2-difluorobenzene in the presence of excess acetonitrile (10 mg, 5.0 eq.). The peak assigned to coordinated acetonitrile shifts slightly with excess MeCN (see zoom-in) and the peak for "free" MeCN shows slight broadening, both due to ligand exchange reactions. No conversion of acetonitrile to di-, tri- or oligomerization products was observed.

Additional NMR Spectra of 1, 1^{d}, 2 and reactants

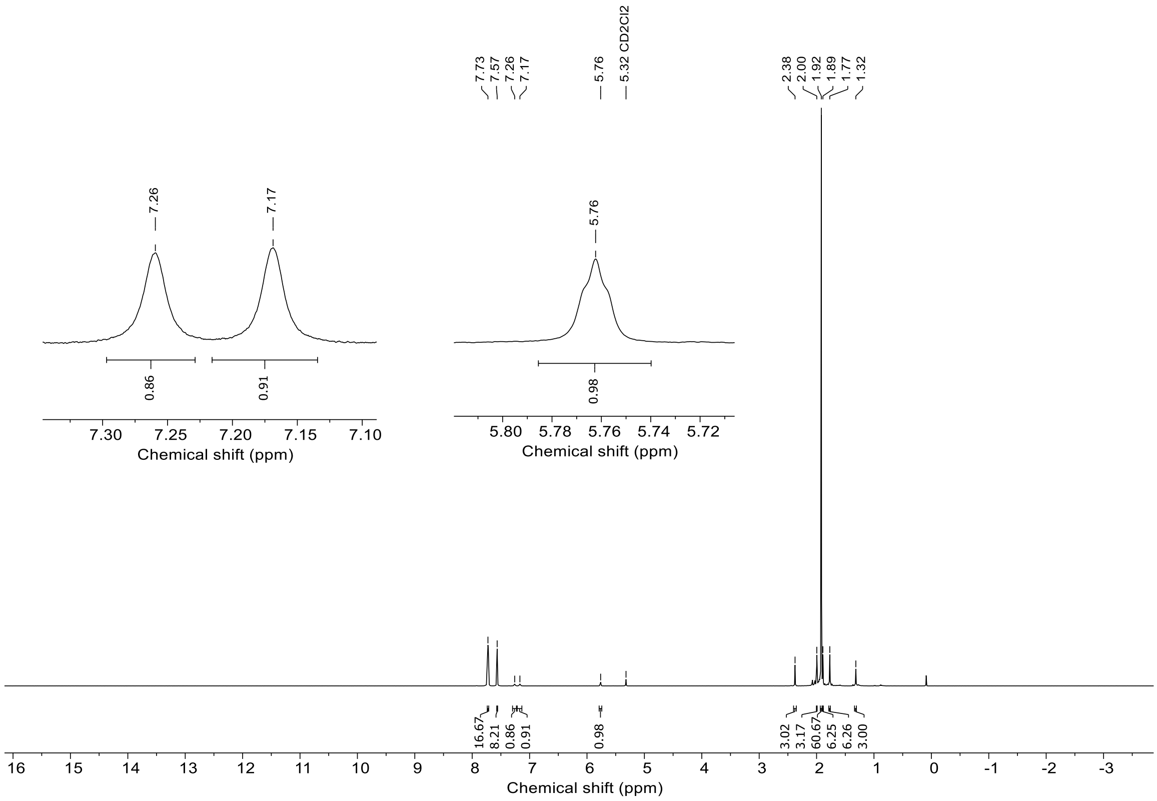

Figure S4: ^{1}H NMR spectrum of **1** in CD_2Cl_2 at 298 K. The NH signals of the Nacnac moiety are observed at 7.27 and 7.18 ppm (see left zoom-in) as well as the backbone CH proton at 5.77 ppm (see right zoom-in).

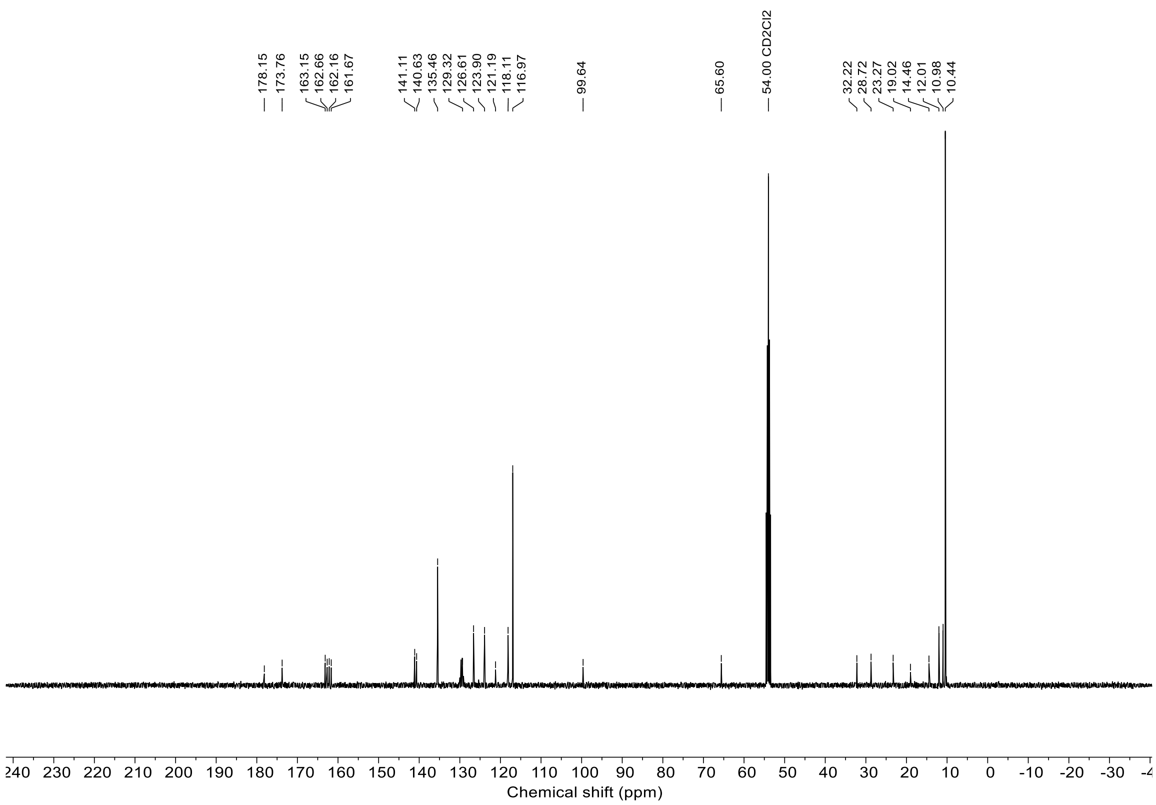

Figure S5: ^{13}C NMR spectrum of **1** in CD_2Cl_2 at 298 K.

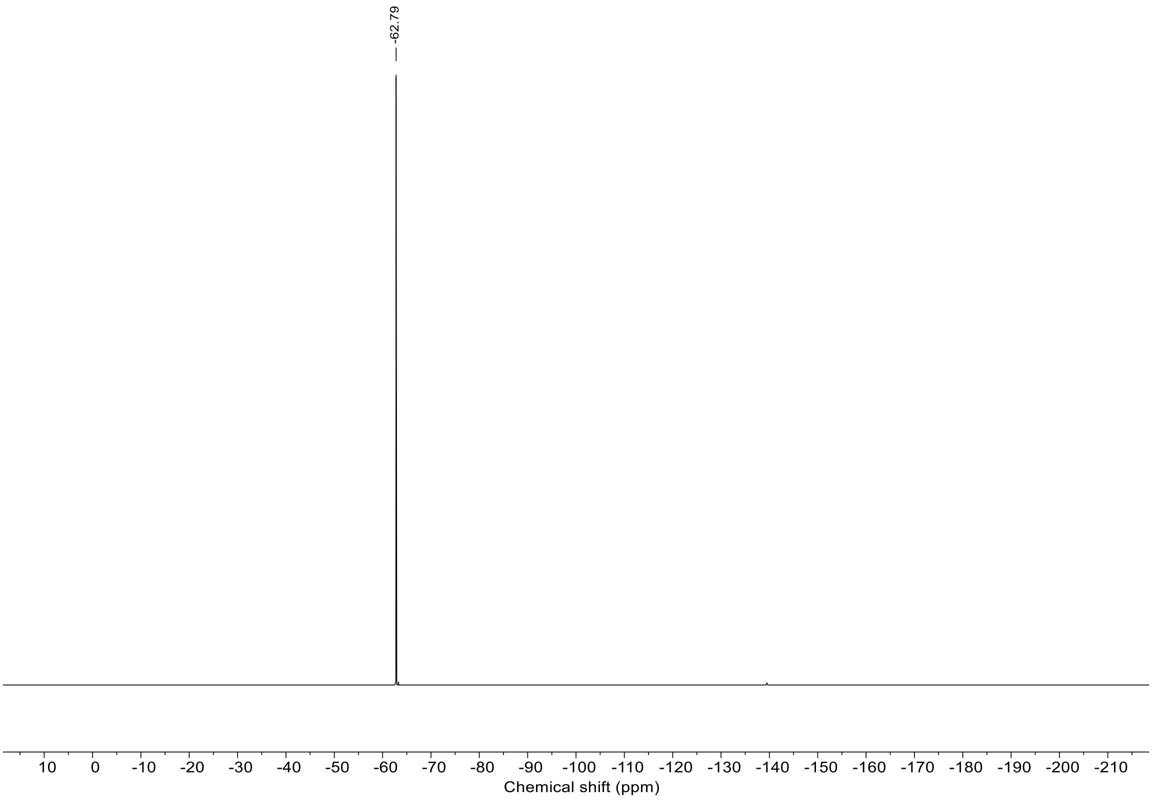

Figure S6: ^{19}F NMR spectrum of **1** in CD_2Cl_2 showing the CF_3 groups of the BAr^F anions.

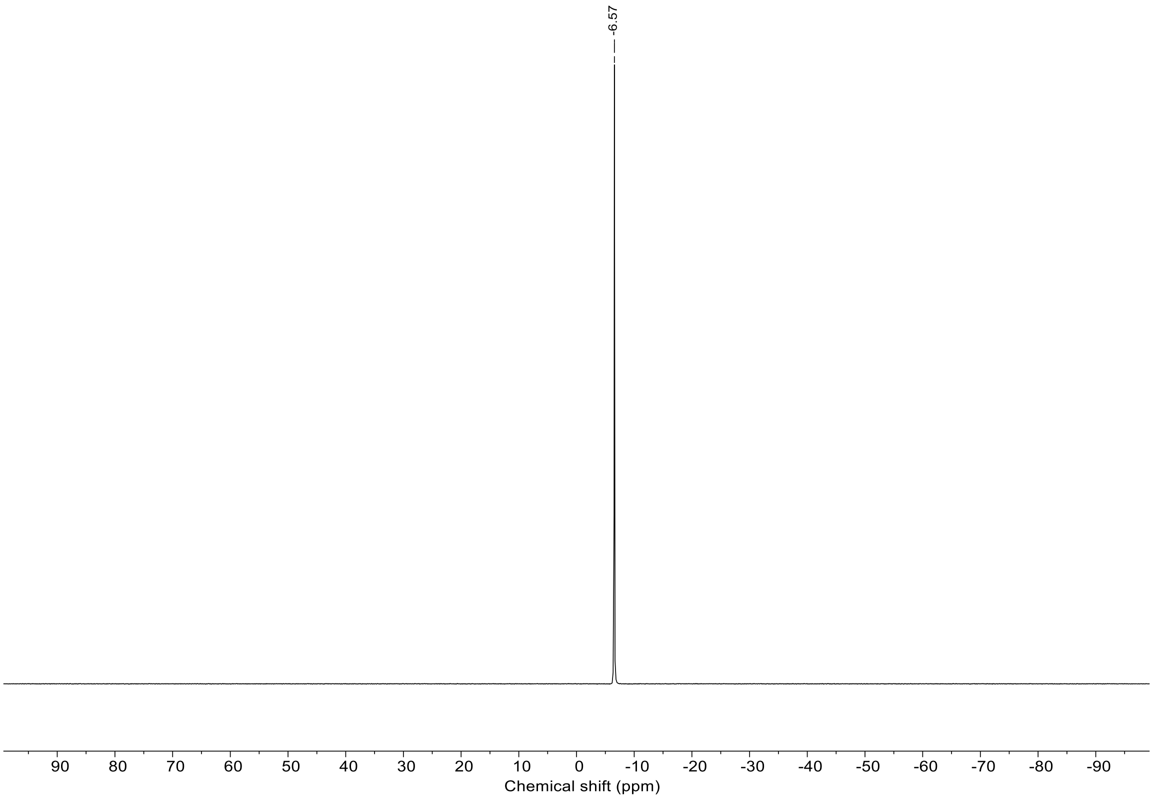

Figure S7: ^{11}B NMR spectrum of **1** in CD_2Cl_2.

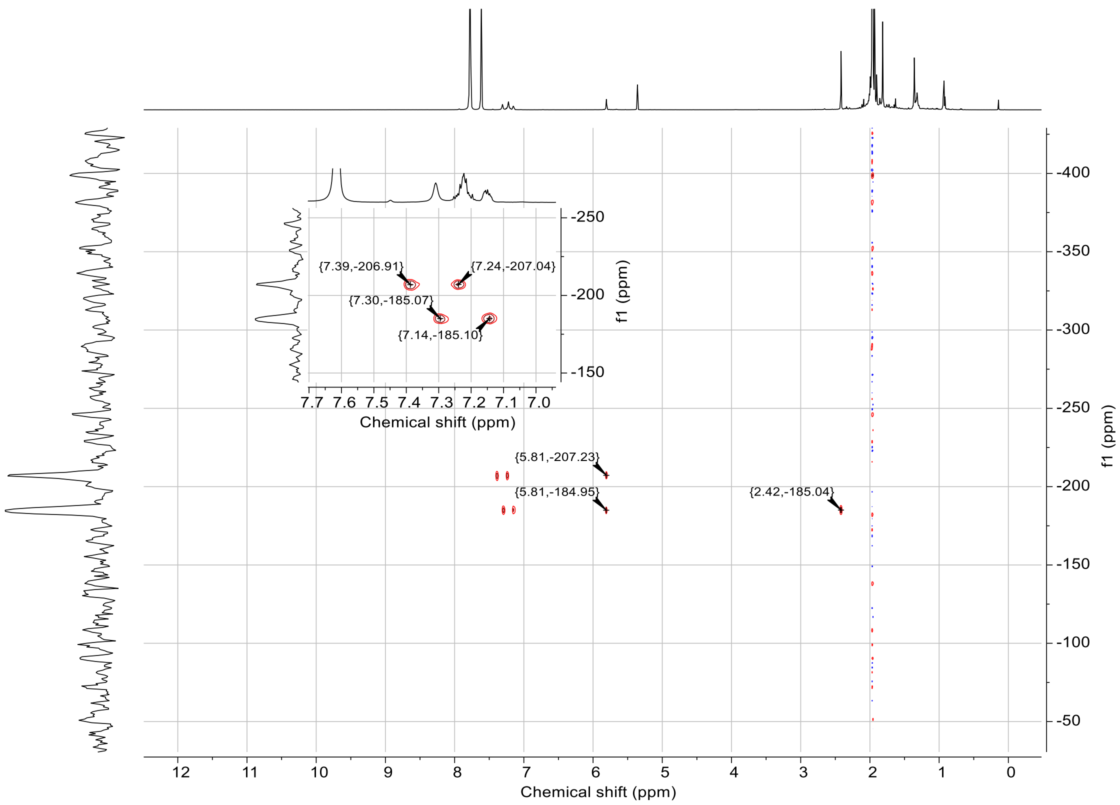

Figure S8: ^1H-^{15}N HMBC spectrum of **1** in CD_2Cl_2 at 298 K, showing ^{15}N signals at 296.98 and 185.08 ppm. The ^1J coupling between NH protons and ^{15}N nuclei can be observed (see zoom-in) as well as ^3J and ^4J couplings to the CH ($\delta(^1H)$ = 5.81 ppm) and CH_3 ($\delta(^1H)$ = 2.42 ppm) groups of the Nacnac moiety.

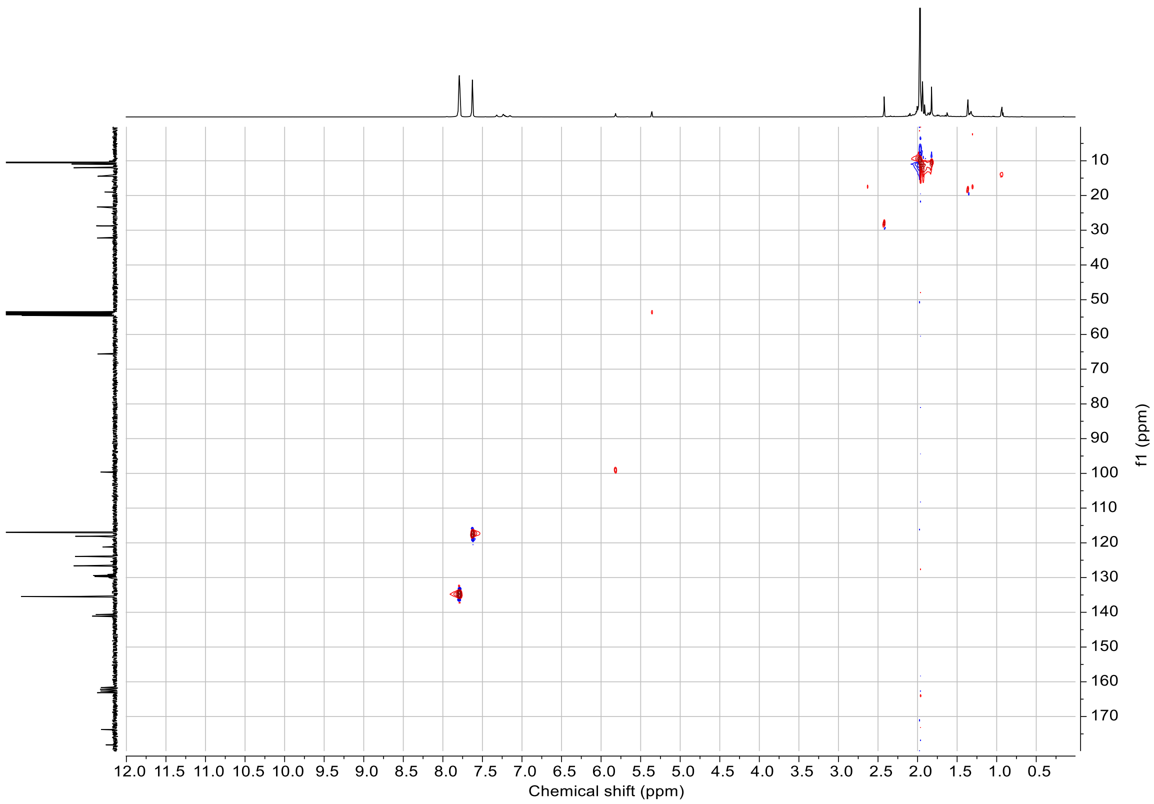

Figure S9: ^1H-^{13}C HSQC spectrum of **1** in CD_2Cl_2 at 298 K.

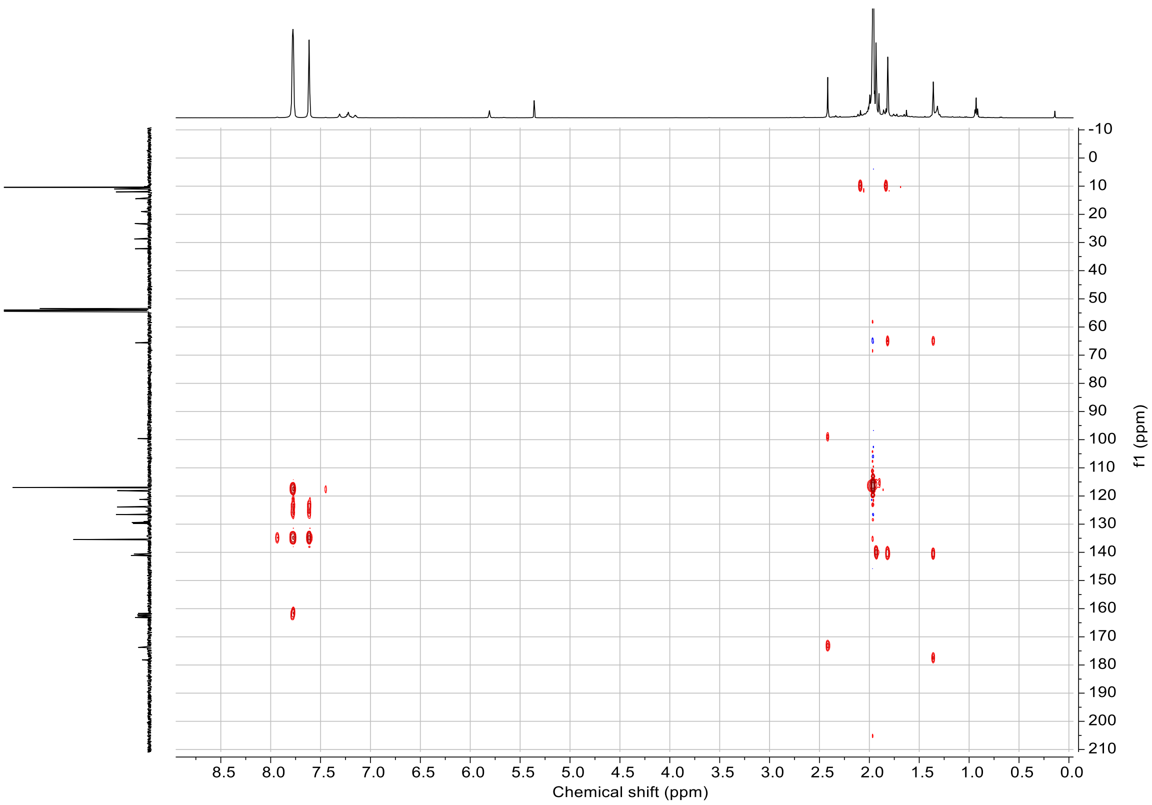

Figure S10: ^{1}H-^{13}C HMBC spectrum of **1** in CD_2Cl_2 at 298 K.

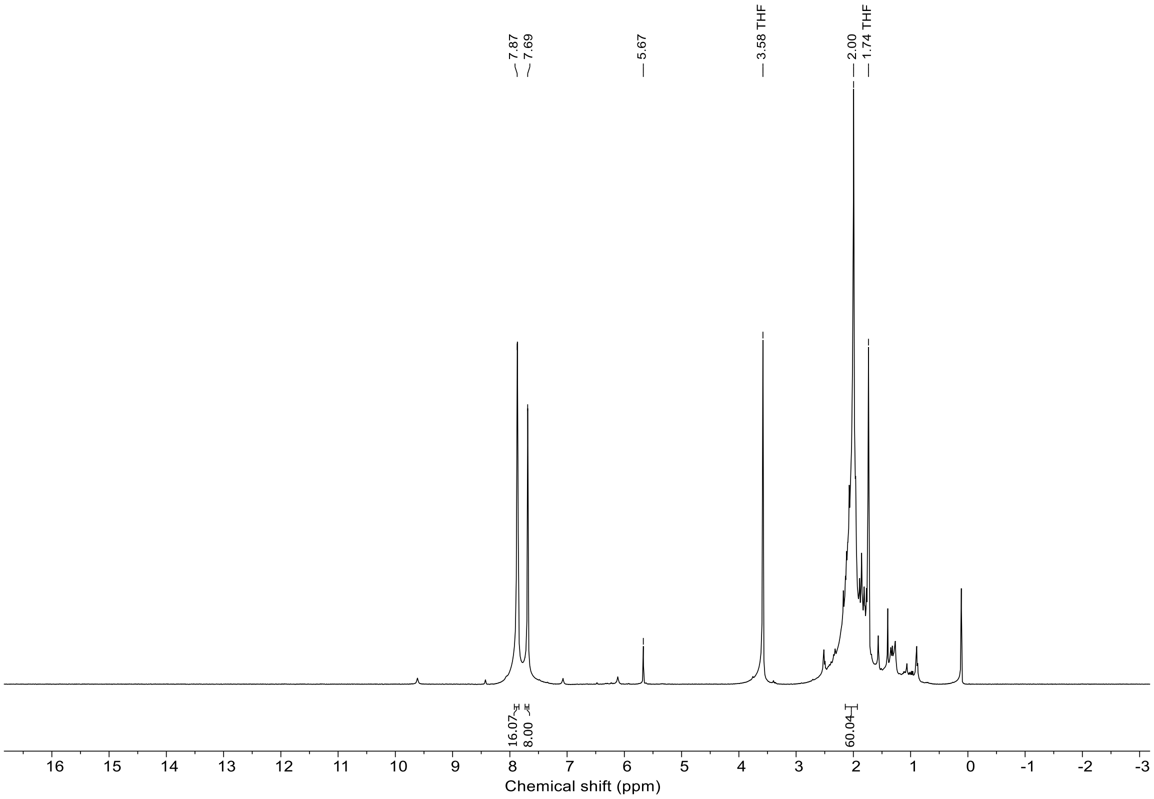

Figure S11: ^{1}H NMR spectrum of **1** in THF-d_8 at 193 K. The spectrum shows no de-coalescence of the peak at 2.00 ppm (GaCp* methyl protons) although three basal and one apical GaCp* moieties are present in the SC-XRD structure of **1**.

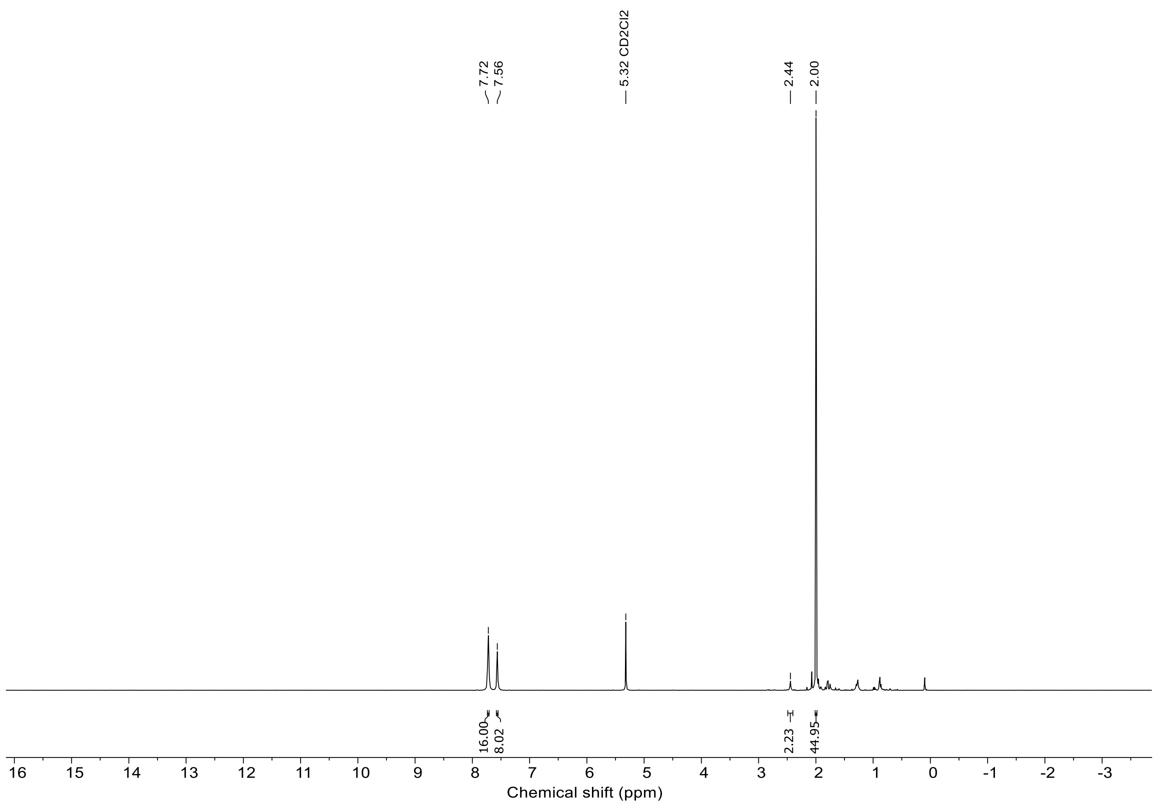

Figure S12: ^{1}H NMR spectrum of [(MeCN)Ni(GaCp*)$_4$](BAr^F)$_2$ (**2**) in CD_2Cl_2 at 273 K.

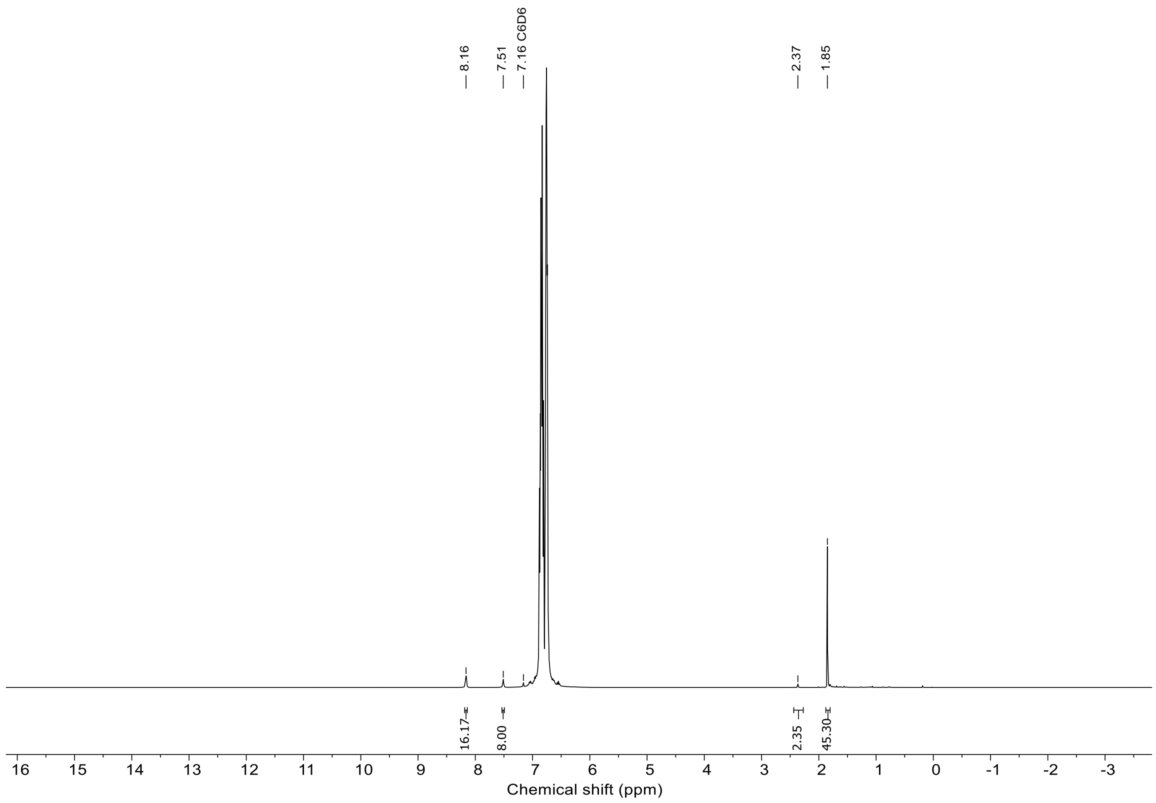

Figure S13: ^{1}H NMR spectrum of [(MeCN)Ni(GaCp*)$_4$](BAr^F)$_2$ (**2**) in 1,2-difluorobenzene (+10% C_6D_6) at 298 K. Compound **2** also slowly decomposes in 1,2-difluorobenzene solution.

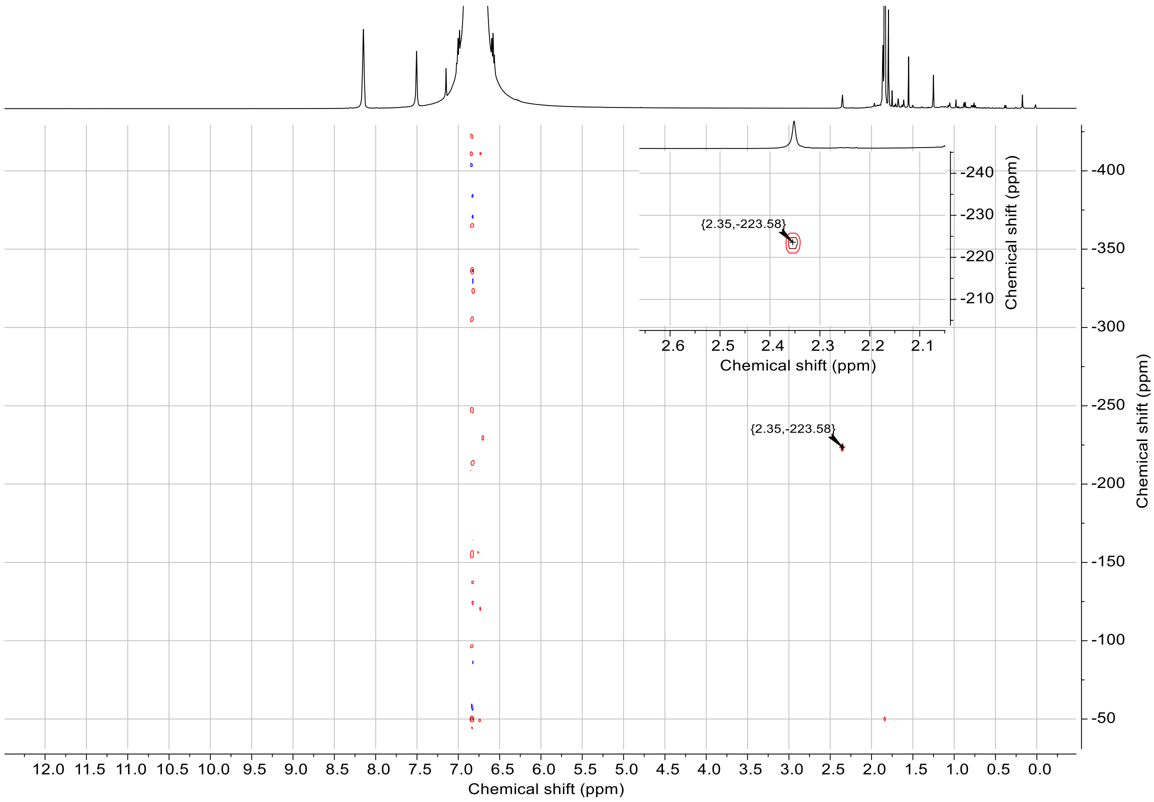

Figure S14: ^{1}H-^{15}N HMBC spectrum of **2** recorded in 1,2-difluorobenzene (+10% C_6D_6) at 298 K. The spectrum nicely shows a ^{15}N peak with a chemical shift of –223.58 ppm attributable to coordinated acetonitrile.

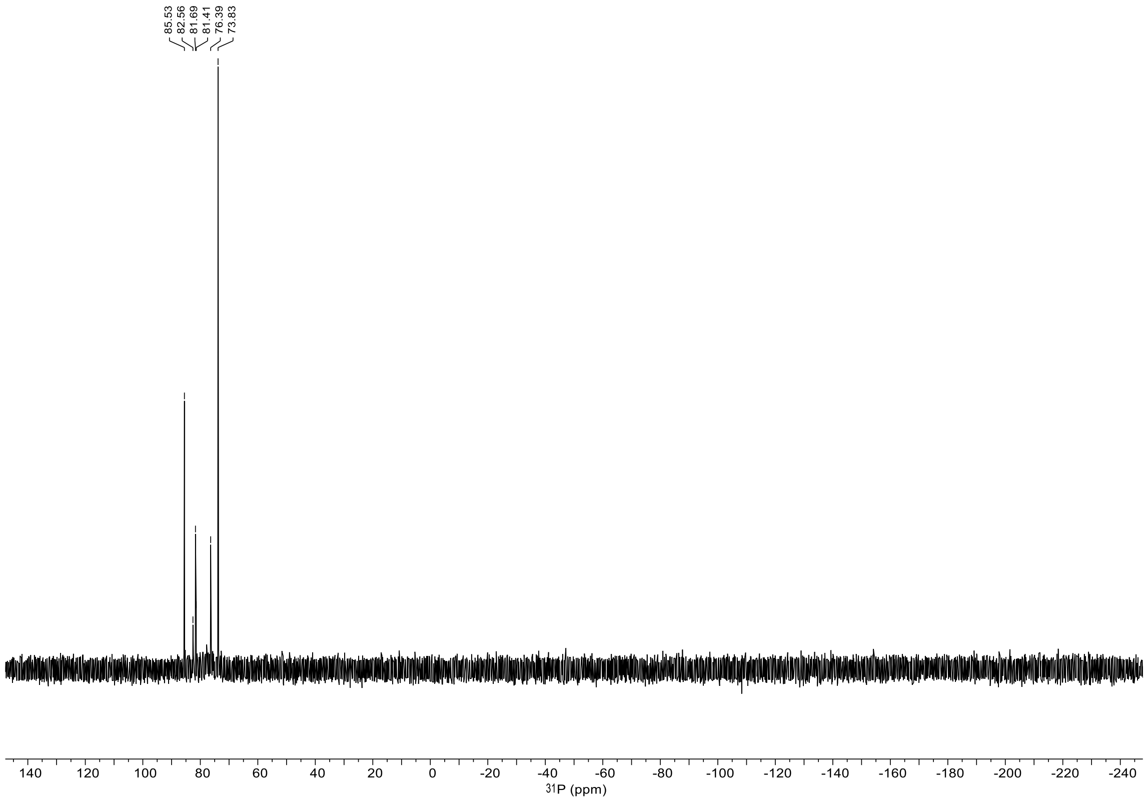

Figure S15: ^{31}P NMR spectrum of **2** in 1,2-dilfuorobenzene solution after addition of 0.8 eq. of triethylphosphine oxide. Multiple signals are observed in the range from 85.53 to 73.83 ppm, which indicates decomposition of **2**.

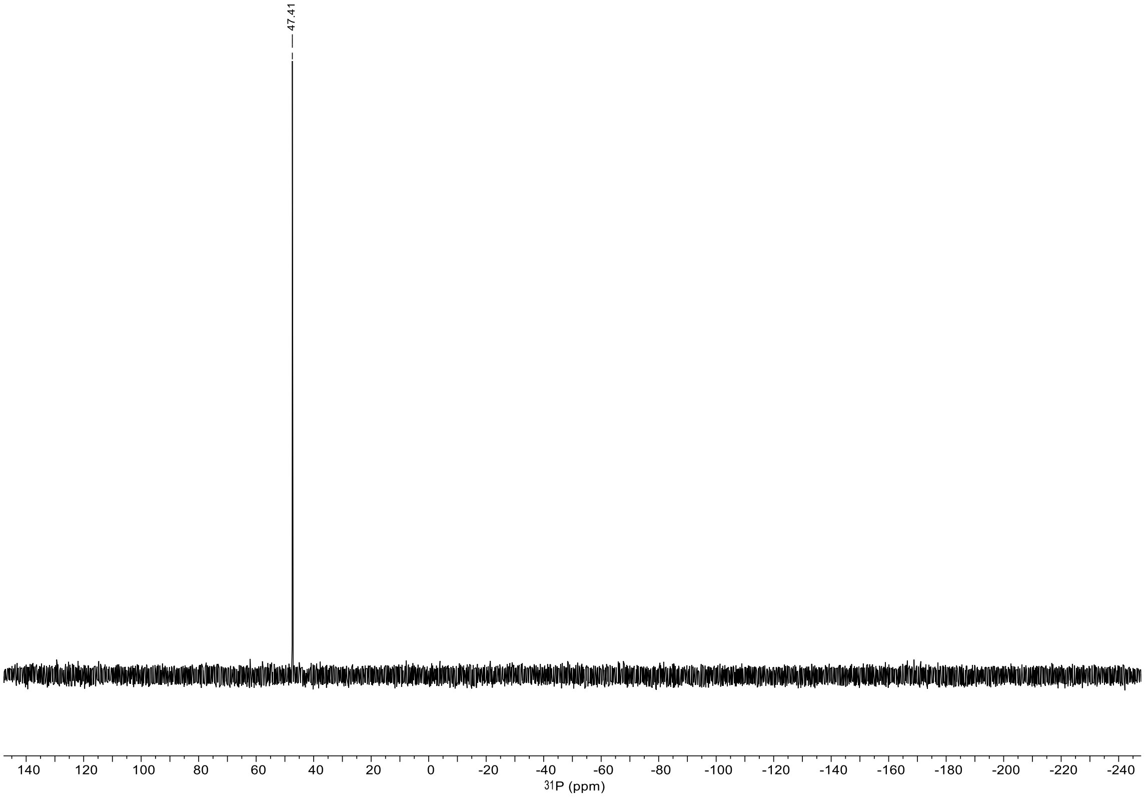

Figure S16: ^{31}P NMR spectrum of triethylphosphine oxide in 1,2-dilfuorobenzene solution.

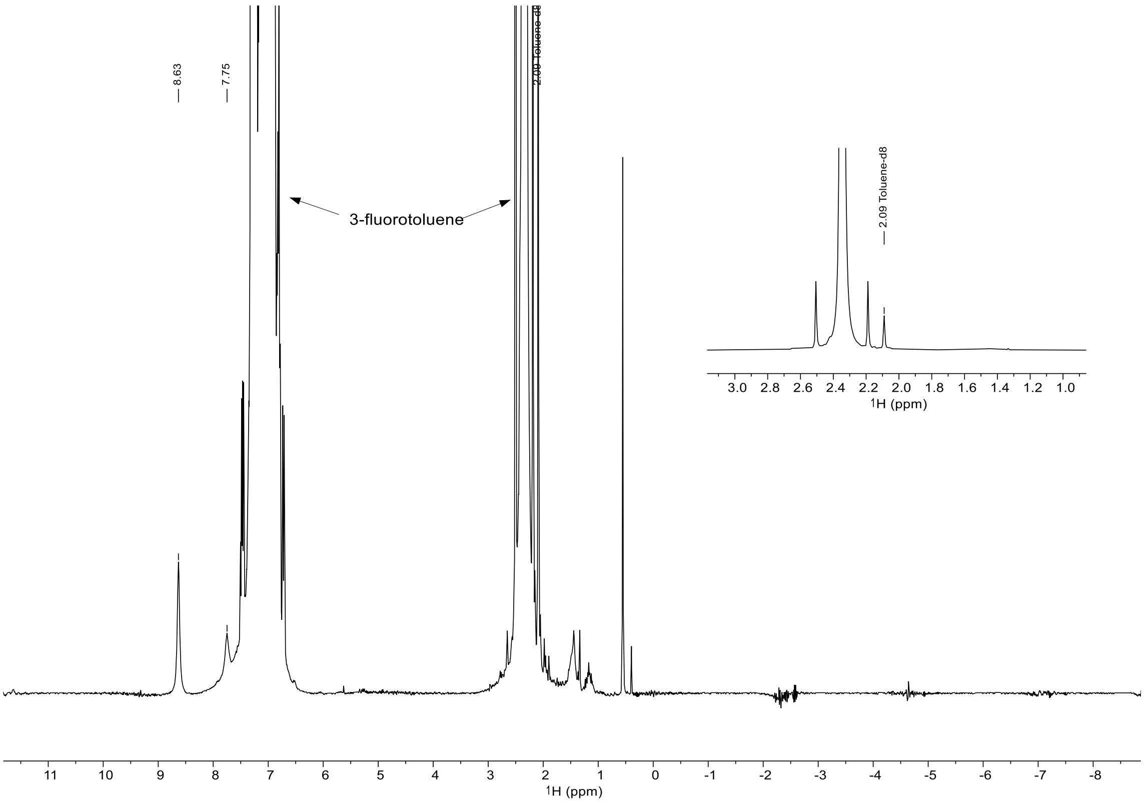

Figure S17: ^{1}H NMR spectrum of **2** in 3-fluorotoluene (locked to toluene-d_8) at 193 K in the presence of one additional equivalent of acetonitrile. The spectrum is largely obscured by the non-deuterated solvents and shows no peaks for adducts of **2** with MeCN.

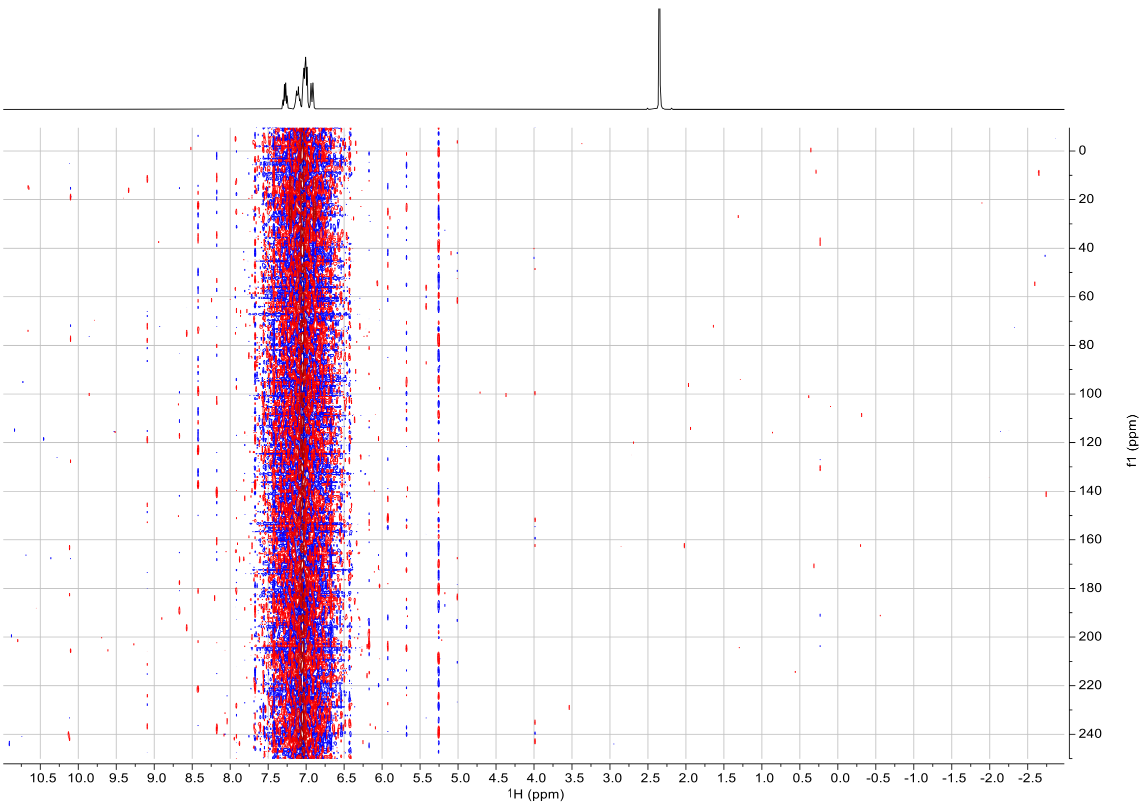

Figure S18: ^{1}H-^{13}C HMBC spectrum of **2** in 3-fluorotoluene (locked to toluene-d_8) at 193 K in the presence of one additional equivalent of acetonitrile. The spectrum shows no correlation peaks for adducts of **2** with MeCN.

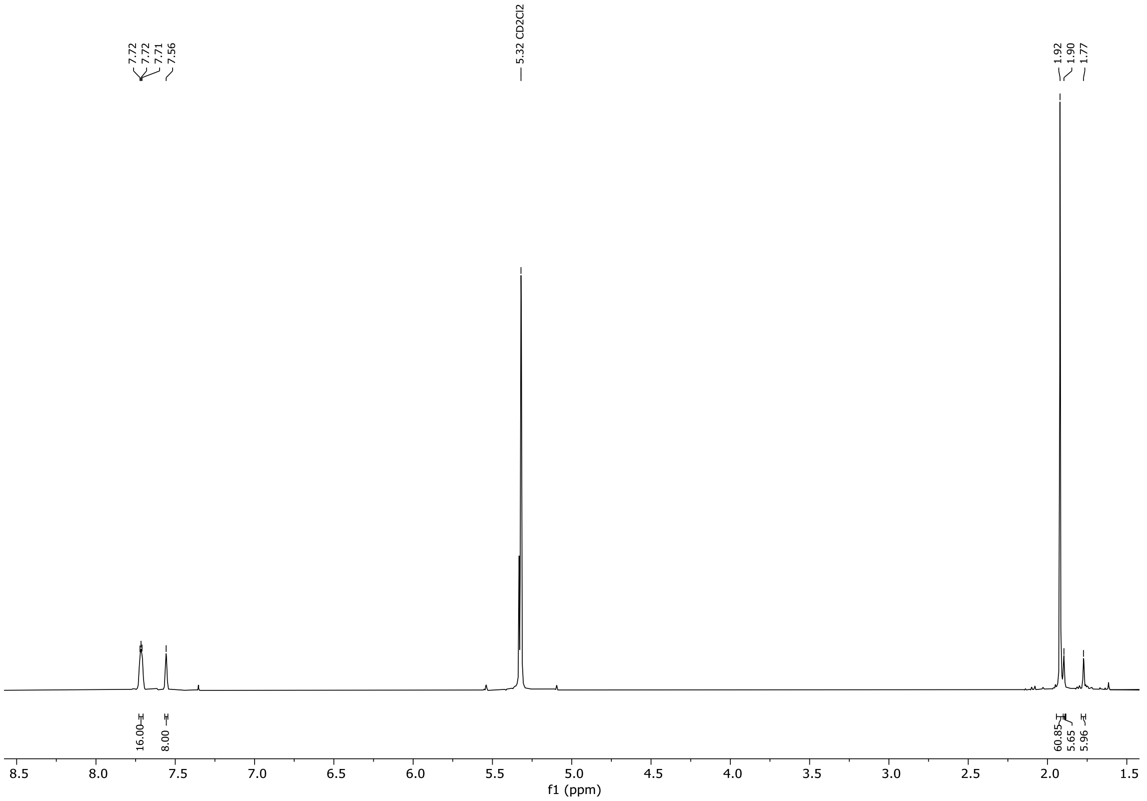

Figure S19: Cut-out of the ^{1}H NMR spectrum of **1**^{d} recorded in CD_2Cl_2 at 298 K. Only the peaks at 1.90 and 1.77 ppm, which correspond to the methyl groups of Cp* moiety incorporated into the nacnac-ligand, are observed. The N–H signals (7.26 and 7.17 ppm), the methine proton (5.76 ppm) and the nacnac methyl group (2.36 ppm) vanish due to deuteration.

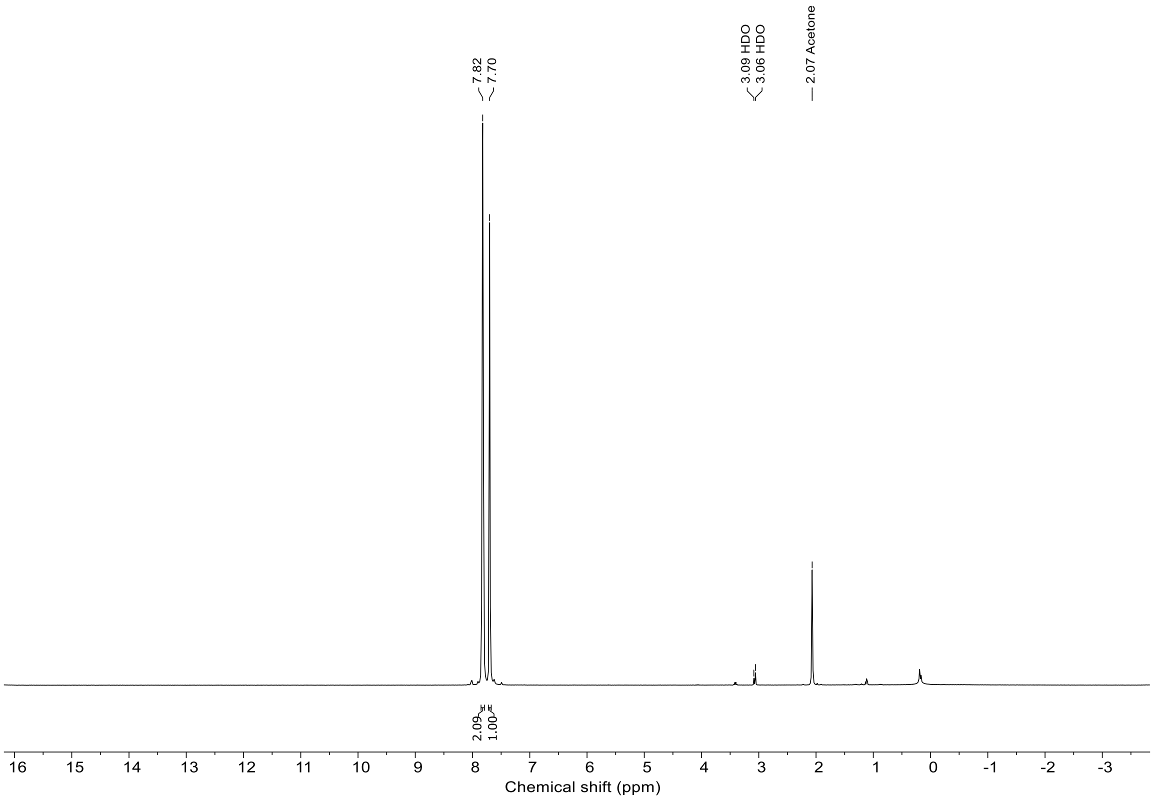

Figure S20: ^{1}H NMR spectrum of NaBAr^F in acetone-d_6 at 298 K. The spectrum shows HDO and H_2O peaks at 3.08 ppm since the NMR solvent was stored in air; the product was dried in vacuo at 110 °C overnight to ensure complete dryness.

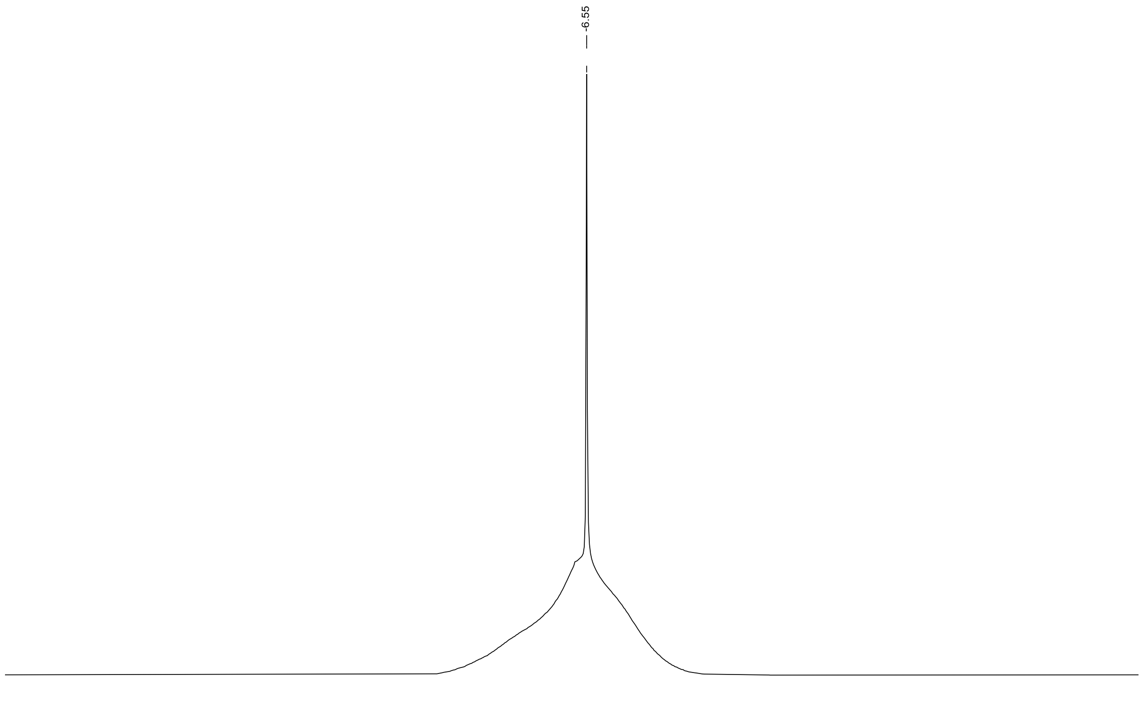

Figure S21: ^{11}B NMR spectrum of NaBAr^F in acetone-d_6 at 298 K.

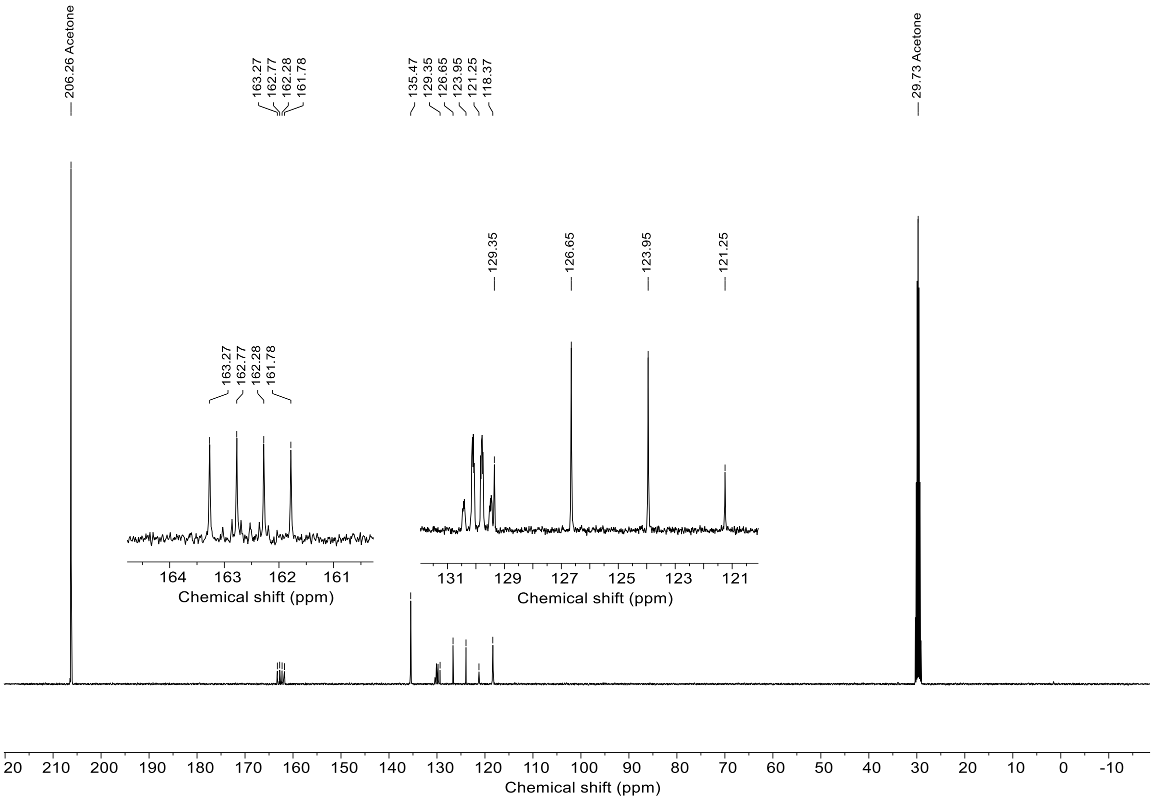

Figure S22: ^{13}C NMR spectrum of $NaBAr^F$ in acetone-d_6 at 298 K.

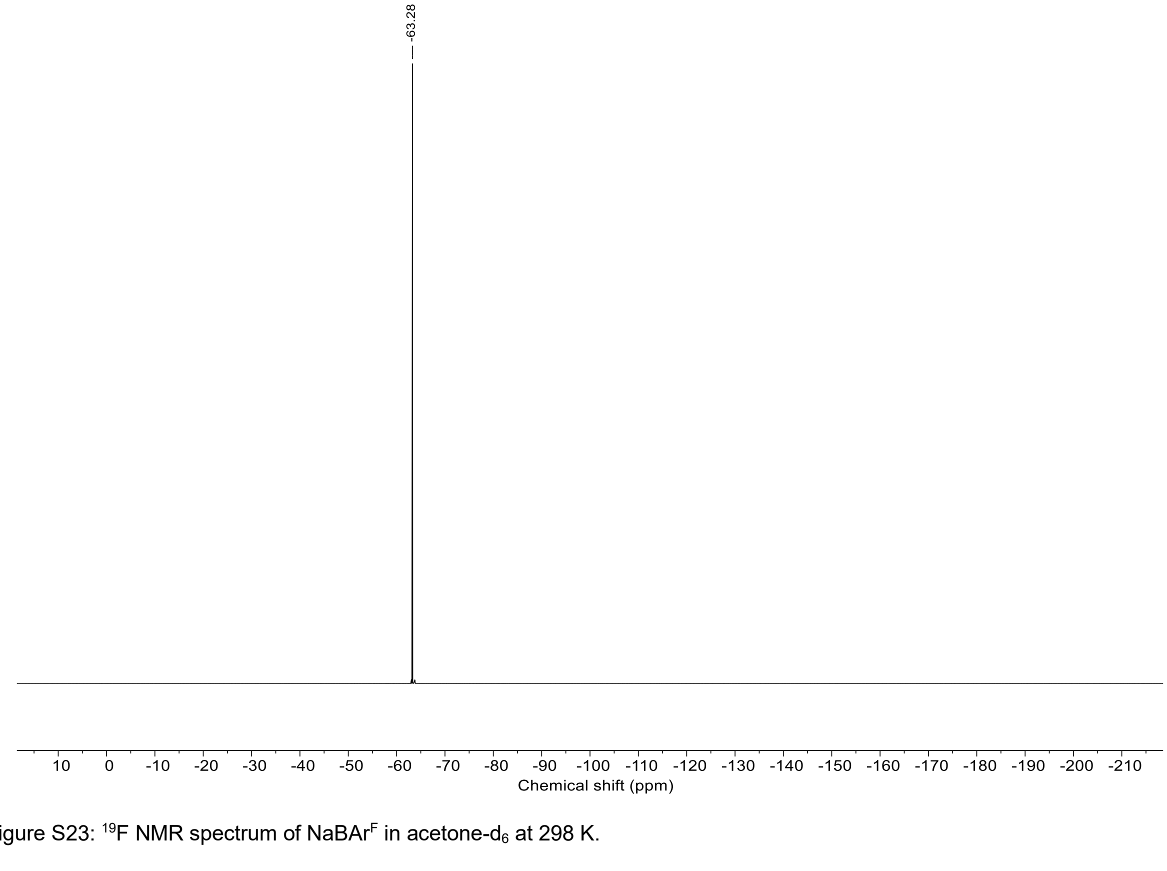

Figure S23: ^{19}F NMR spectrum of $NaBAr^F$ in acetone-d_6 at 298 K.

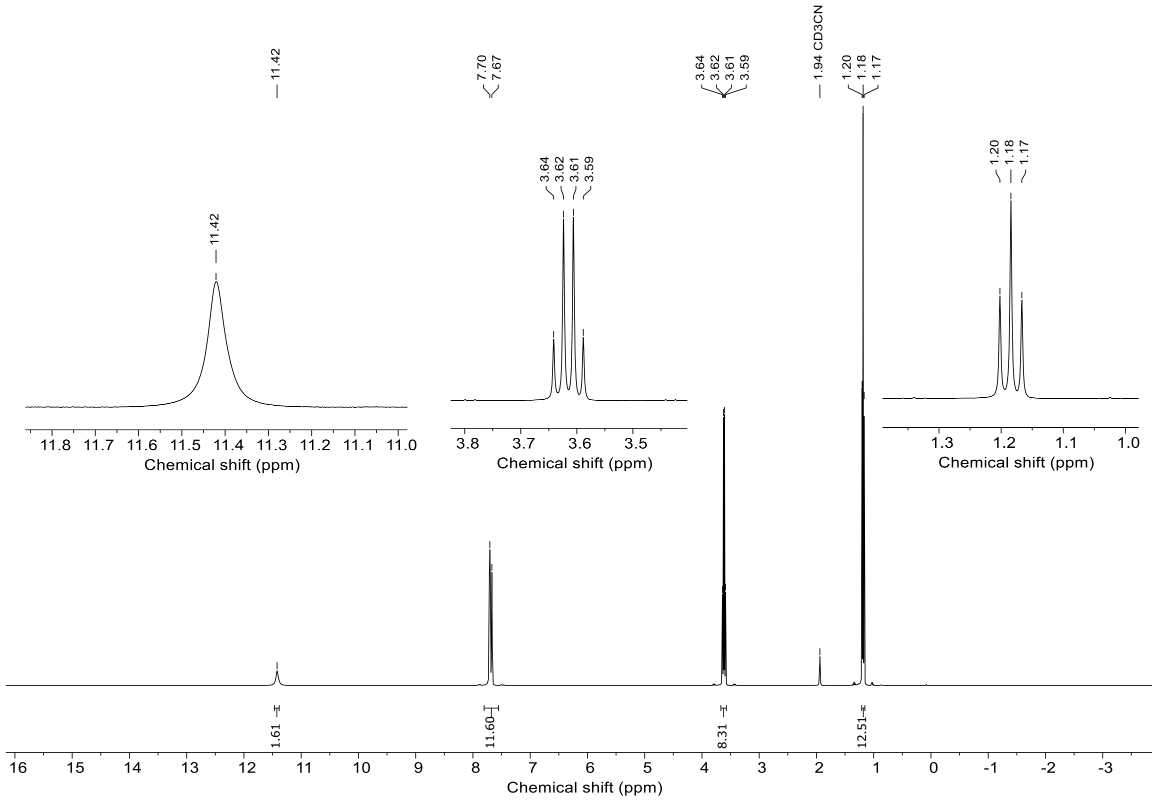

Figure S24: ^{1}H NMR spectrum of $H(Et_2O)_2BAr^F$ in CD_3CN at 298 K.

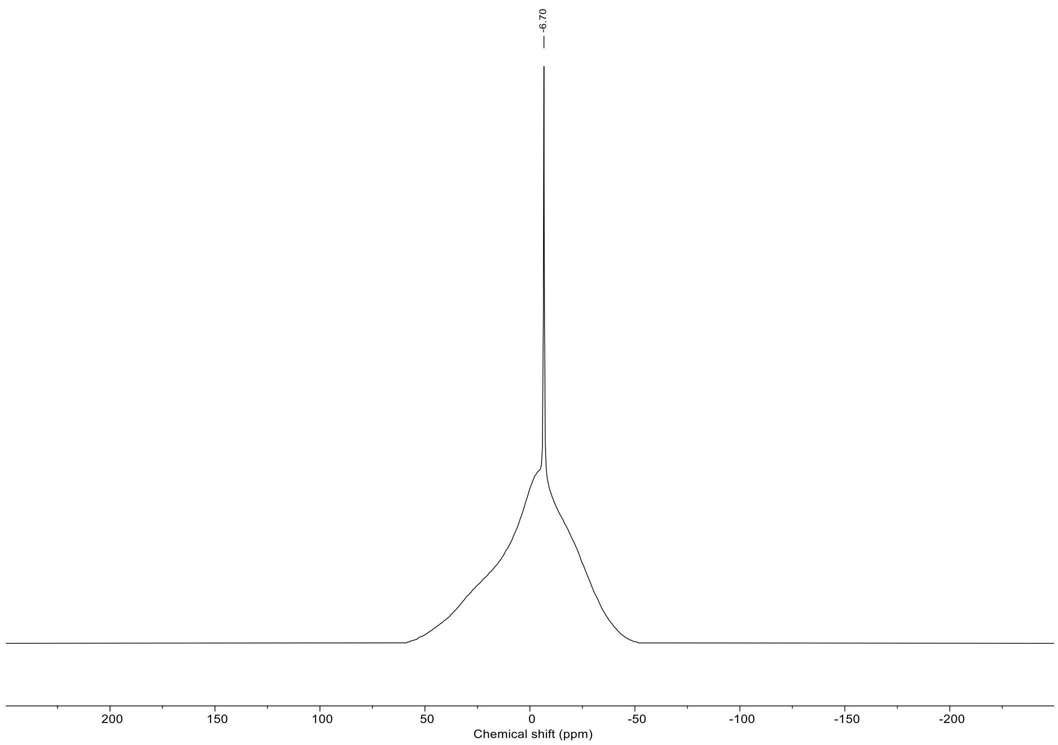

Figure S25: ^{11}B NMR spectrum of $H(Et_2O)_2BAr^F$ in CD_3CN at 298 K.

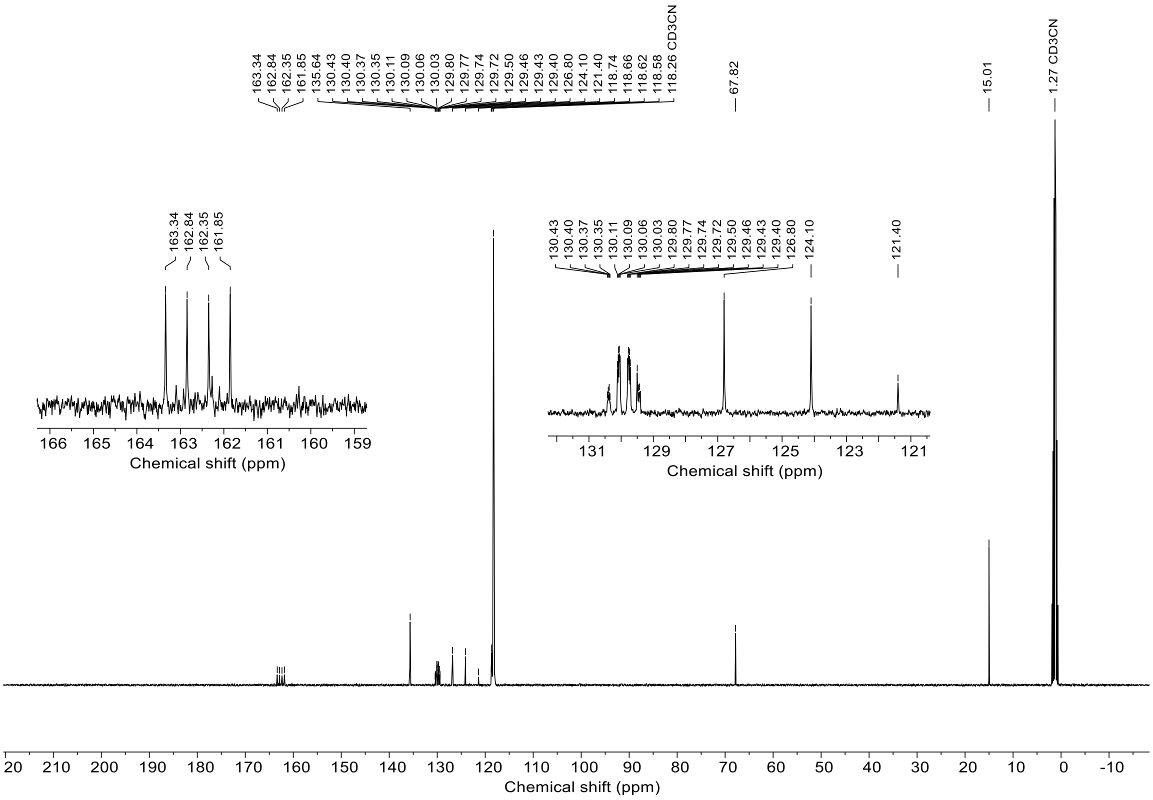

Figure S26: ^{13}C NMR spectrum of $H(Et_2O)_2BAr^F$ in CD_3CN at 298 K.

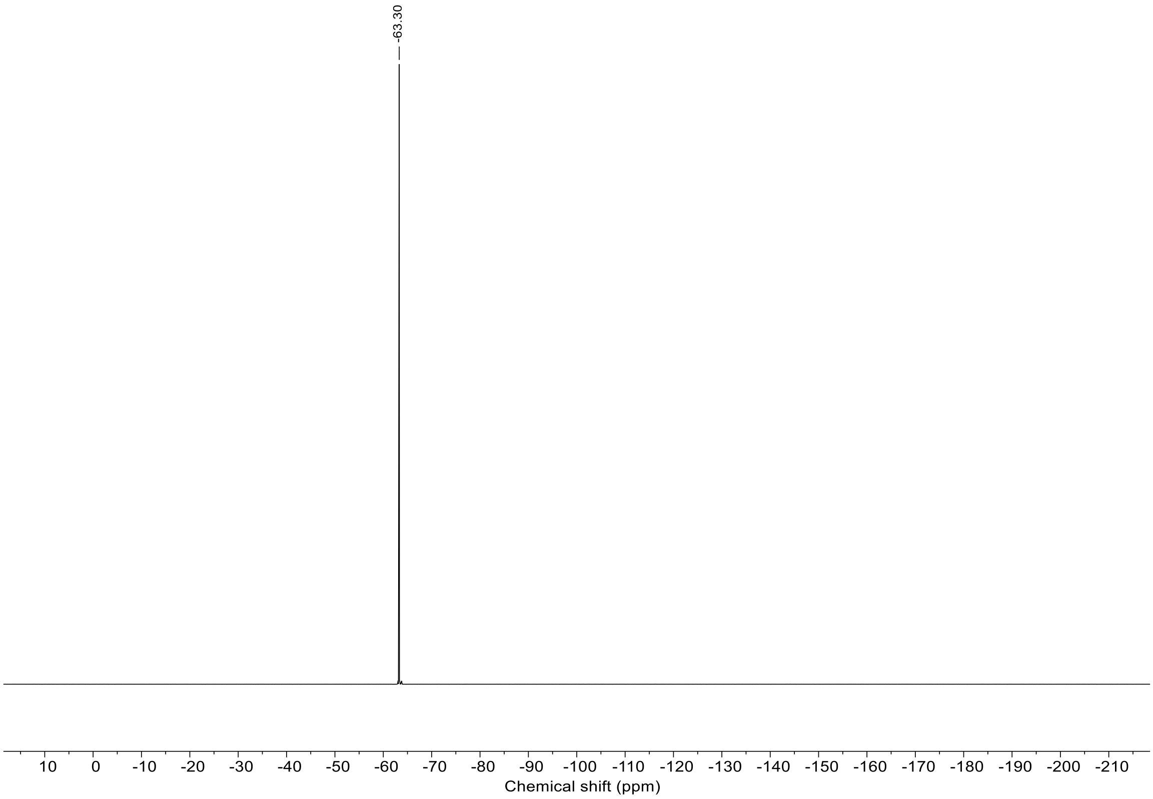

Figure S27: ^{19}F NMR spectrum of $H(Et_2O)_2BAr^F$ in CD_3CN at 298 K.

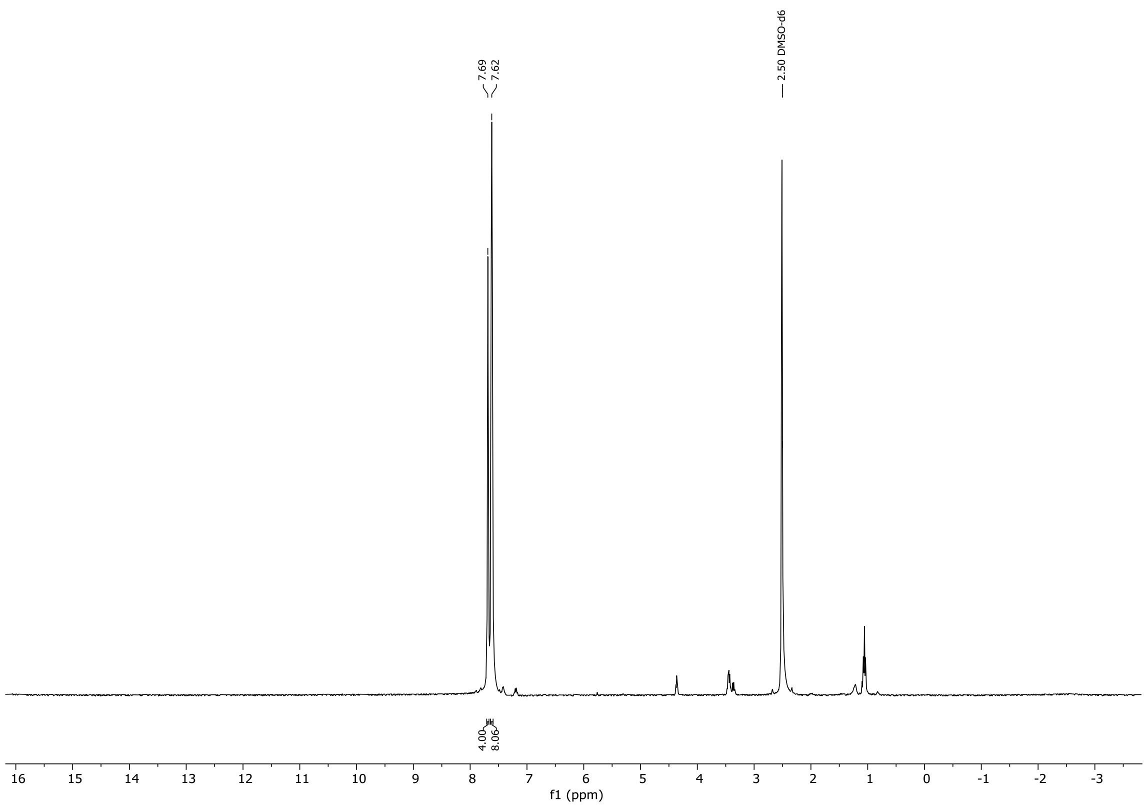

Figure S28: ^{1}H NMR spectrum of Tl(BAr^{F}) in DMSO-d_6 at 298 K. The spectrum shows traces (<5%) of the starting material TlOEt at ca. 3.5 ppm as well as 1 ppm.

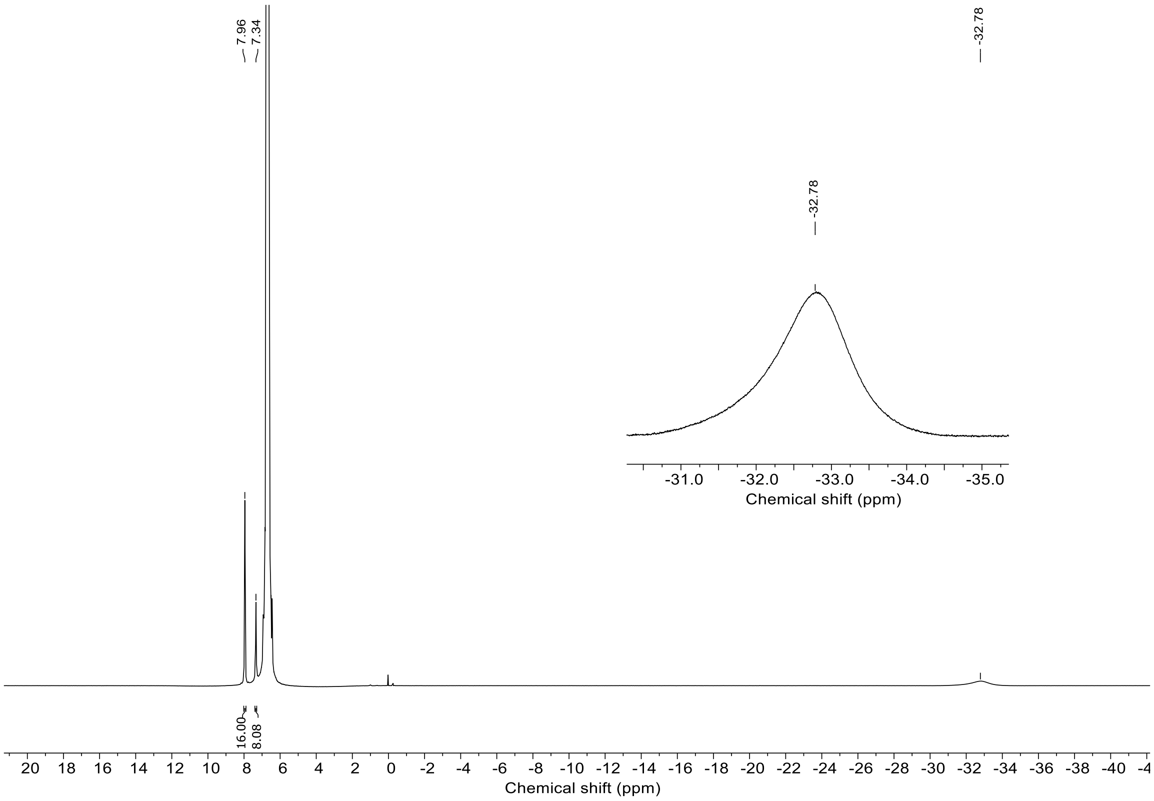

Figure S29: ^{1}H NMR spectrum of $[Ni(MeCN)_6](BAr^F)_2$ in 1,2-DFB showing signals at 8.60 and 7.99 ppm (BAr^{F} anions) as well as –32.14 ppm (acetonitrile ligands) with a characteristic chemical shift for paramagnetic compounds.

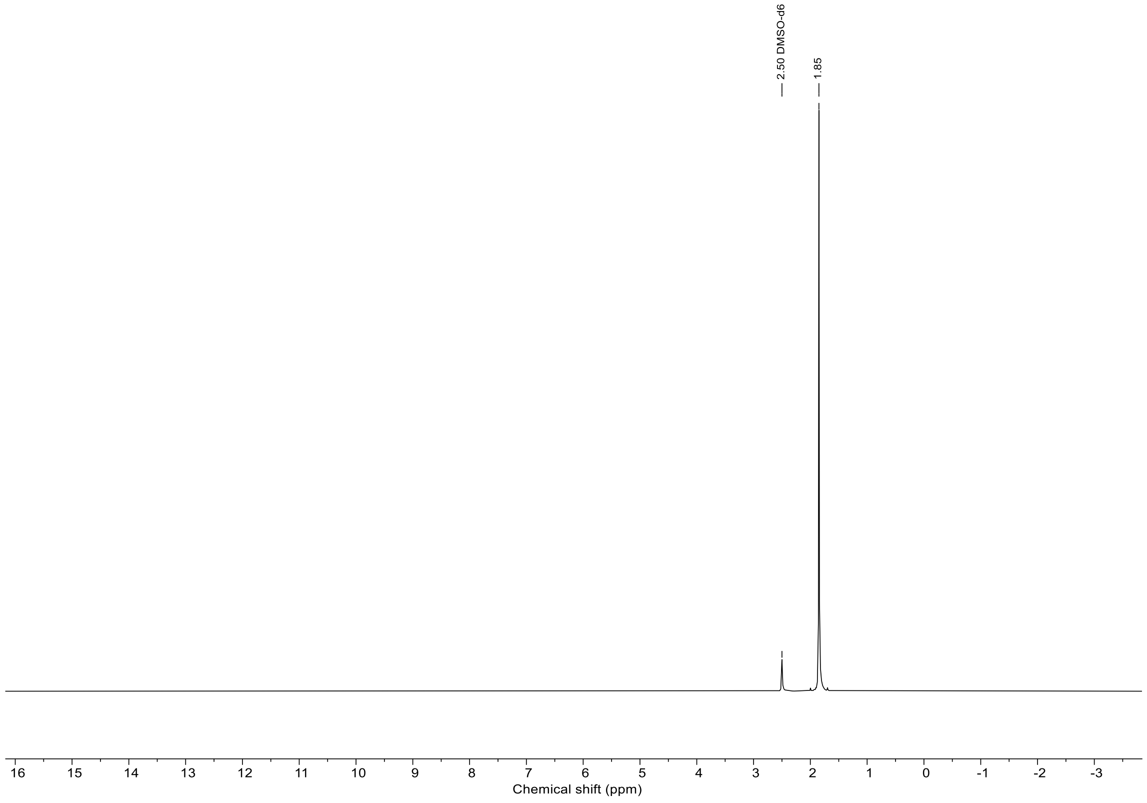

Figure S30: ^{1}H NMR spectrum of KCp* in DMSO-d_6, showing the product peak at 1.85 ppm.

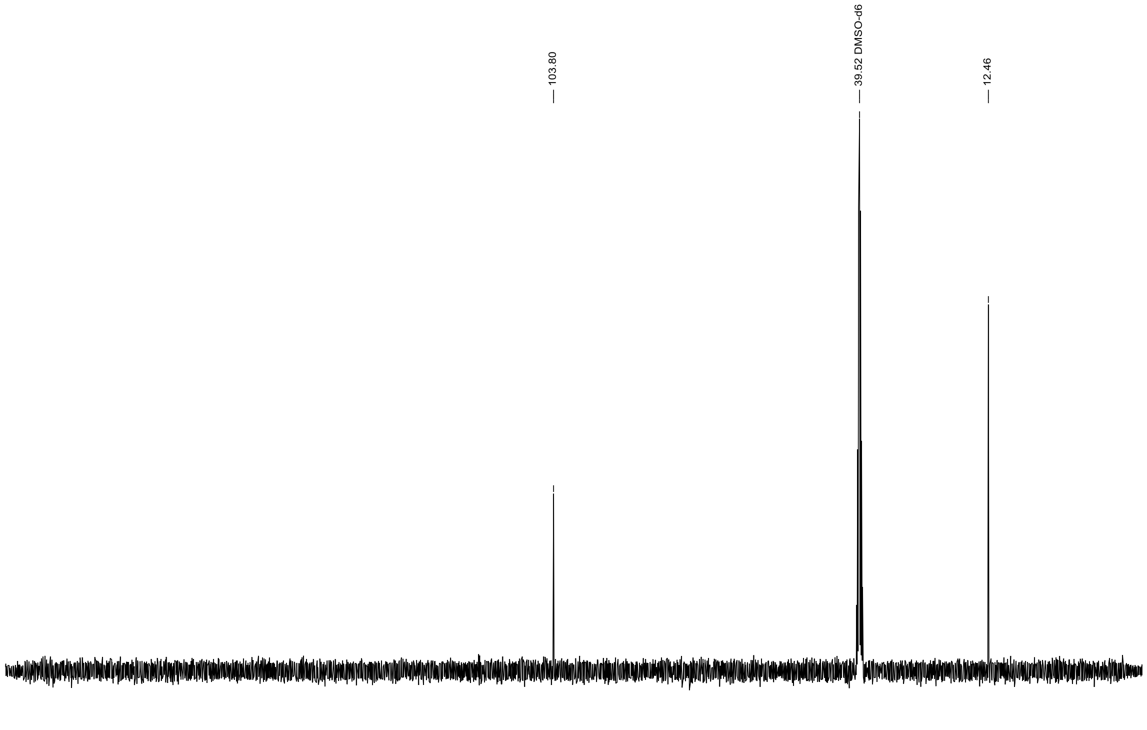

Figure S31: ^{13}C NMR spectrum of KCp* in DMSO-d_6, showing the peaks of the C_5 ring at 103.80 ppm as well as the methyl group carbon atoms at 12.46 ppm.

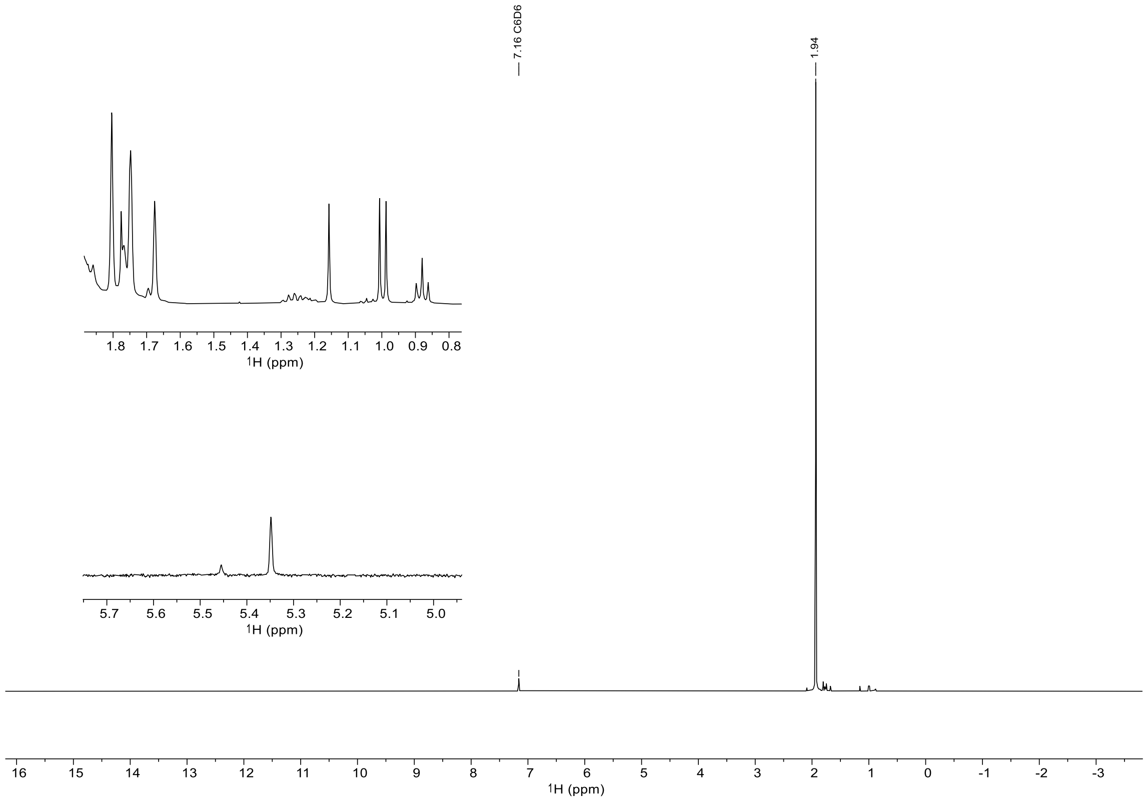

Figure S32: ¹H NMR spectrum of GaCp* in C_6D_6, showing the product peak at 1.94 ppm. Traces (<2%) of Cp*H and $(Cp^*)_2$ (1.9 – 0.9 ppm) as well as tetramethylfulvene (5.35 ppm) may be observed as characteristic side products.

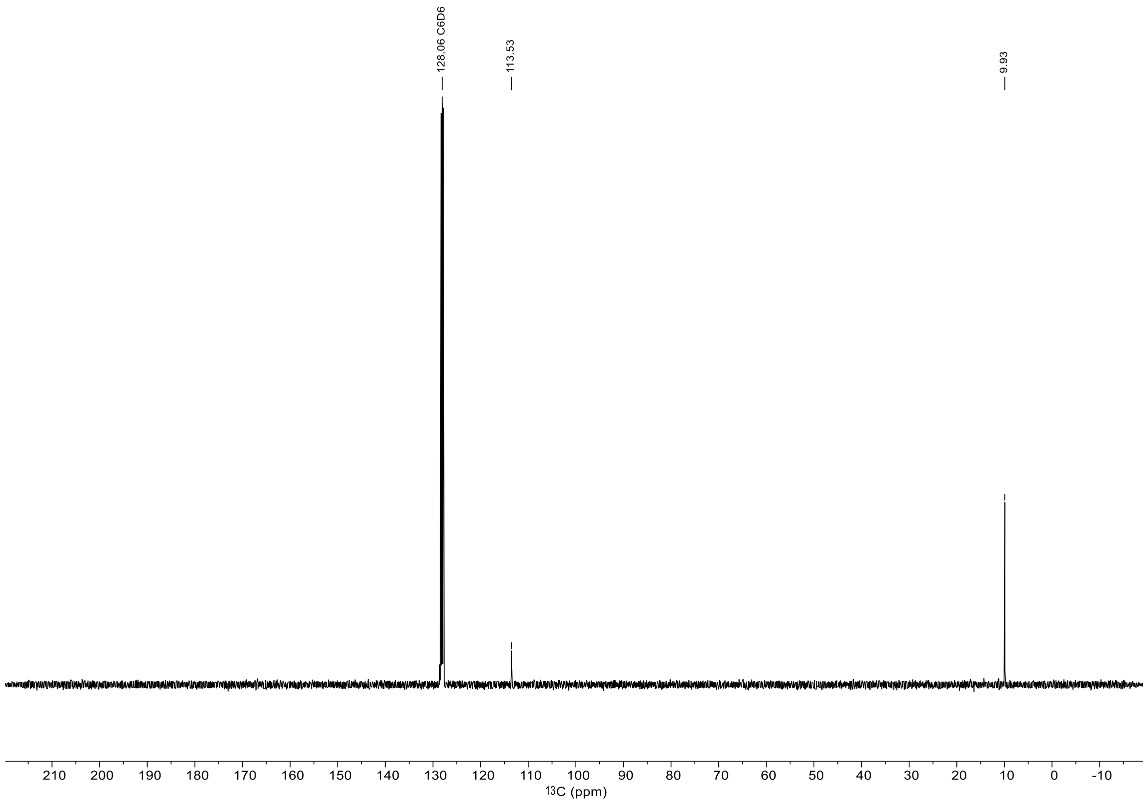

Figure S33: ¹³C NMR spectrum of GaCp* in C_6D_6, showing the peaks of the C_5 ring at 113.53 ppm as well as the methyl group carbon atoms at 9.93 ppm.

4. LIFDI MS & IR Spectroscopic Data

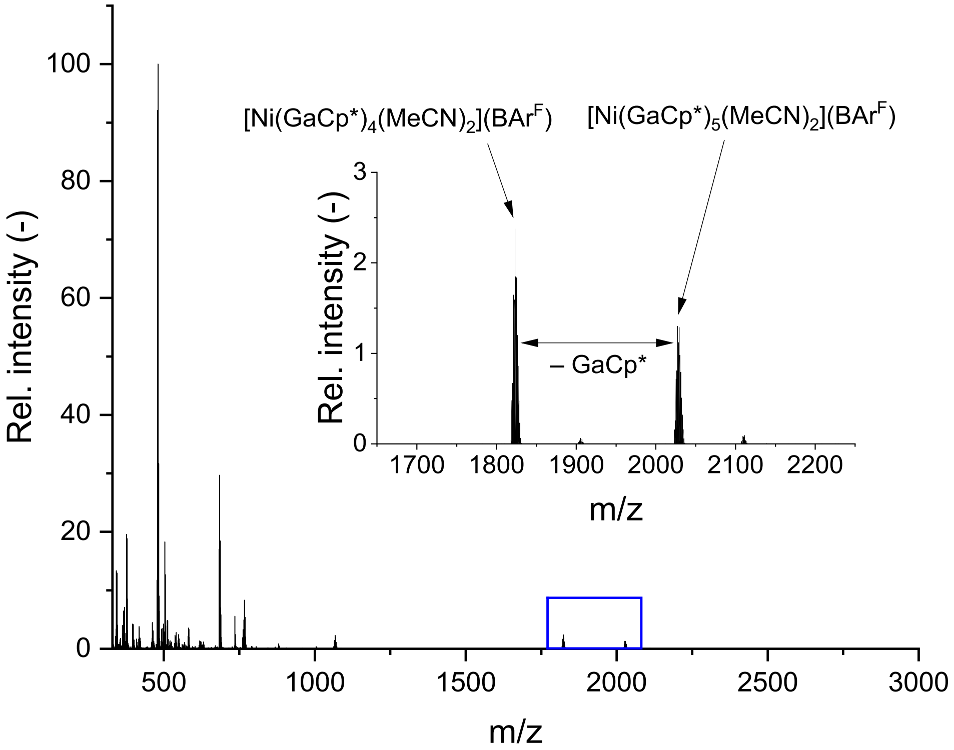

Figure S34: Cut out of the LIFDI mass spectrum of **1** in dichloromethane solution. Due to the high sensitivity of **1**, fragmentation is observed. Nevertheless, the spectrum clearly shows the molecular ion including one BAr^F anion at m/z = 2027 as well as one peak corresponding to the fragment ion [M] –GaCp* at m/z = 1822 (see zoom-in).

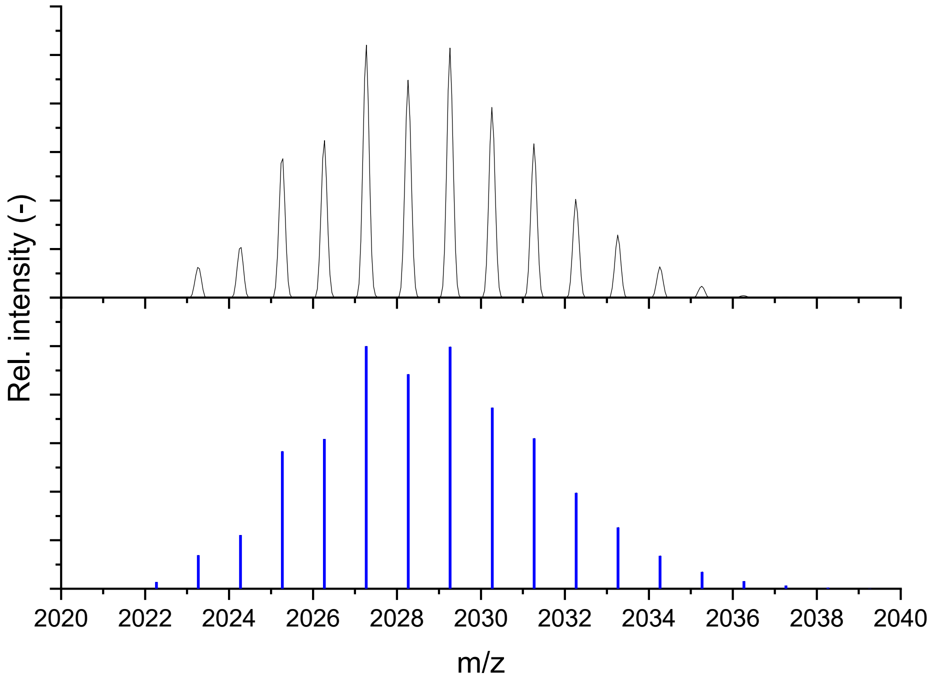

Figure S35: Comparison of the experimentally obtained pattern for $[Ni(GaCp^*)_5(MeCN)_2BAr^F]^+$ (top, black) and the calculated pattern for $NiGa_5C_{86}H_{93}N_2BF_{24}^+$ (bottom, blue).

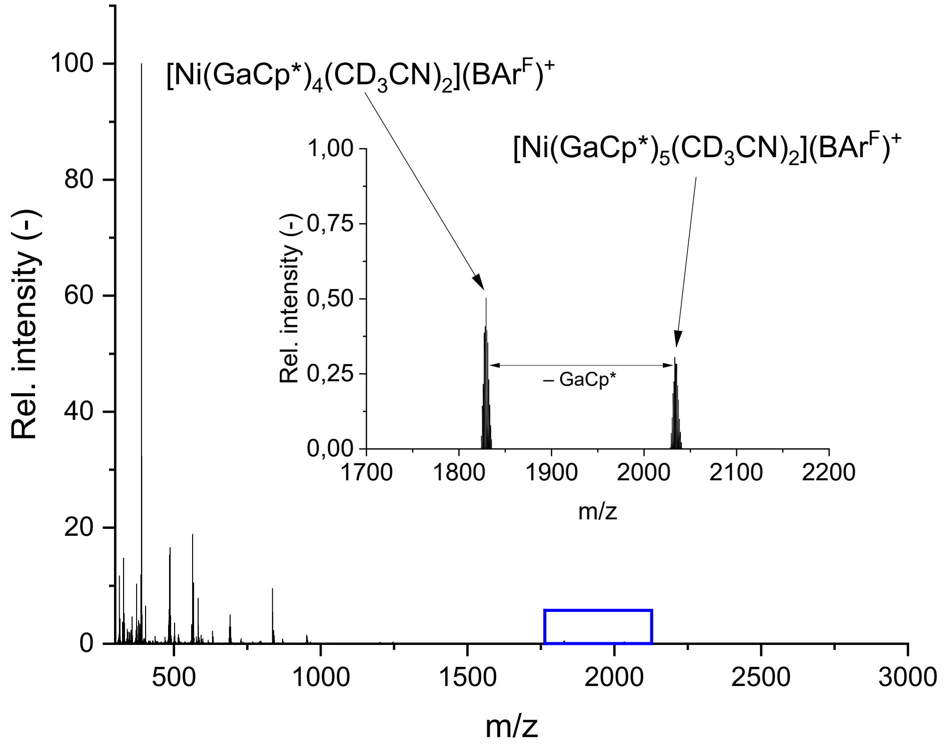

Figure S36: Cut out of the LIFDI mass spectrum of **1**[d] in dichloromethane solution. Analogously to **1**, fragmentation is observed. Nevertheless, the spectrum clearly shows the molecular ion including one BAr^F anion at m/z = 2033 as well as one peak corresponding to the fragment ion [M] –GaCp* at m/z = 1825 (see zoom-in).

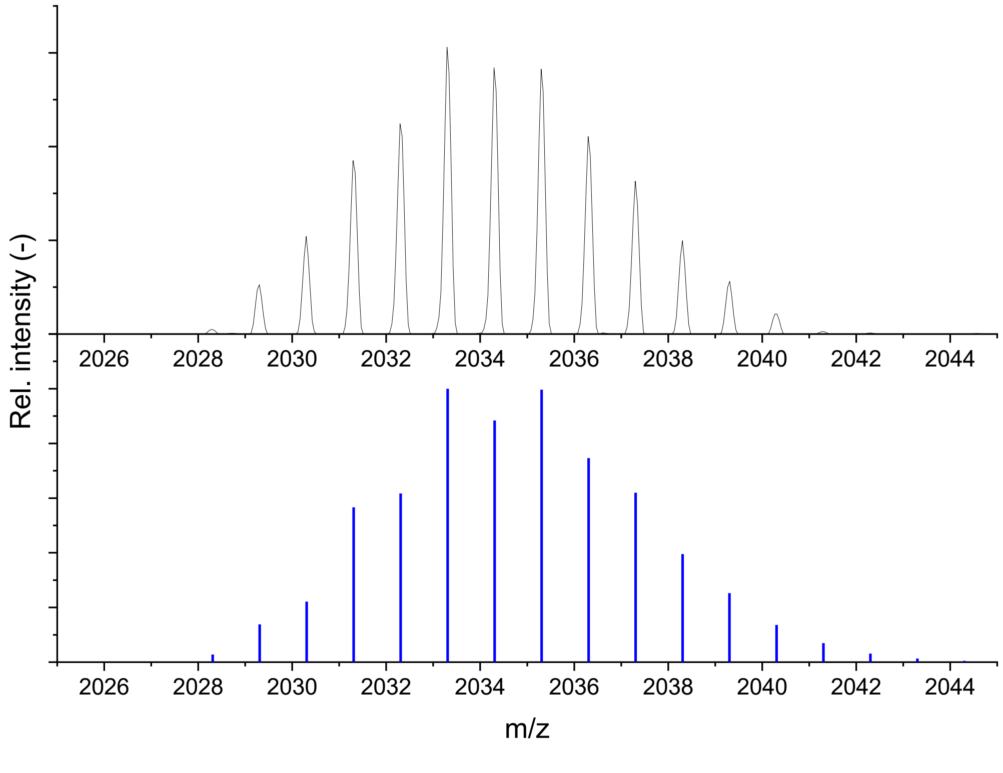

Figure S37: Comparison of the experimentally obtained pattern for $[Ni(GaCp^*)_5(CD_3CN)_2BAr^F]^+$ (top, black) and the calculated pattern for $NiGa_5C_{86}H_{87}D_6N_2BF_{24}^+$ (bottom, blue).

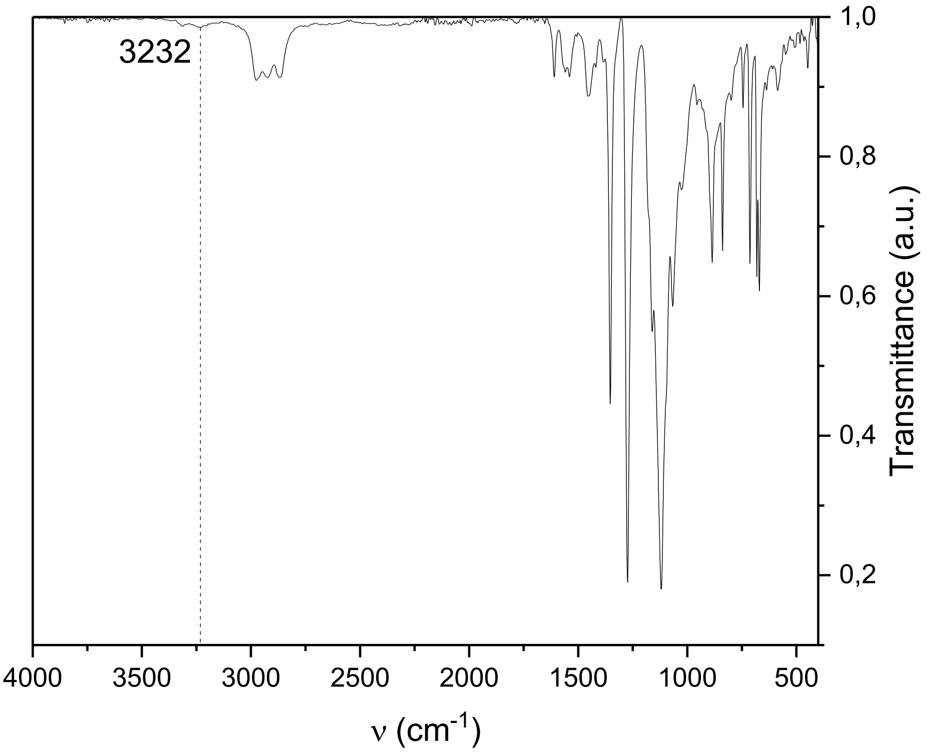

Figure S38: ATR-IR spectrum of $[Ni(GaCp^*)_5(MeCN)_2](BAr^F)_2$ (**1**) showing characteristic bands for a N-H stretching vibration of the Nacnac-type ligand at 3232 cm^{-1} as well as intensive bands attributable to C-F bonds of the BAr^F anions (1353, 1275 and 1110 cm^{-1}).

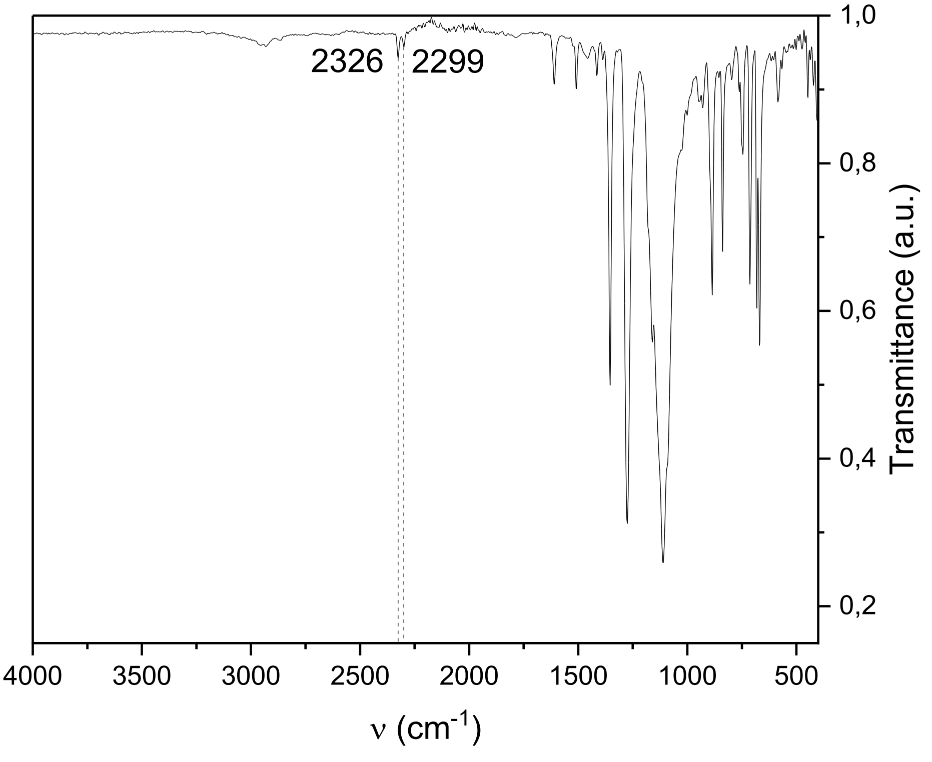

Figure S39: ATR-IR spectrum of $[(MeCN)Ni(GaCp^*)_4](BAr^F)_2$ (**2**) showing characteristic bands for coordinated acetonitrile at 2326 and 2299 cm^{-1} as well as intensive bands attributable to C-F bonds of the BAr^F anions (1353, 1275 and 1110 cm^{-1}).

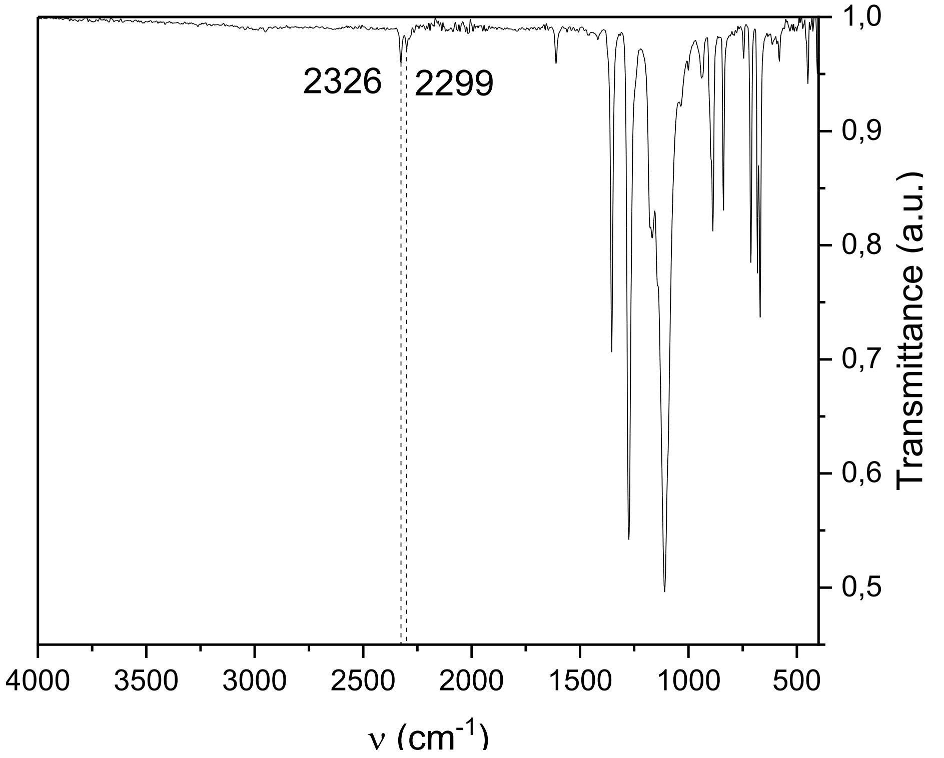

Figure S40: ATR-IR spectrum of $[Ni(MeCN)_6](BAr^F)_2$ showing characteristic bands for coordinated nitriles at 2326 cm^{-1} and 2299 cm^{-1} as well as intensive bands attributable to vibrations of C-F bonds (1353, 1275 and 1110 cm^{-1}). The C-N stretching bands at 2326 and 2299 cm^{-1} are well consistent with literature values for the $[Ni(MeCN)_6]^{2+}$ cation.[2, 14]

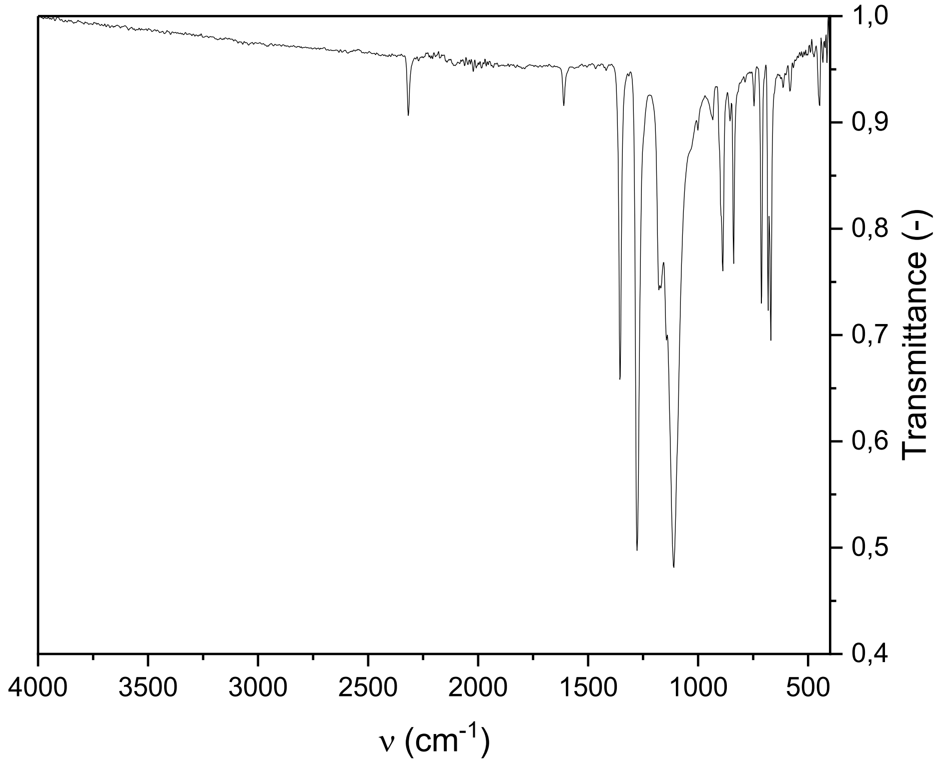

Figure S41: ATR-IR spectrum of $[Ni(MeCN\text{-}d_3)_6](BAr^F)_2$ showing characteristic bands for a C–D stretching vibration at 2326 cm^{-1} as well as intensive bands attributable to vibrations of C-F bonds (1353, 1275 and 1110 cm^{-1}). The data match literature values for the $[Ni(MeCN\text{-}d_3)_6]^{2+}$ cation.[15]

5. UV-Vis Spectra

In order to gain insights into the formation mechanism of **1**, we monitored the reaction using UV-Vis spectroscopy. Compound **2**, which forms at the first stage of the reaction, shows a characteristic broad absorption band in its absorption spectrum at around 531 nm (see Figure S42).

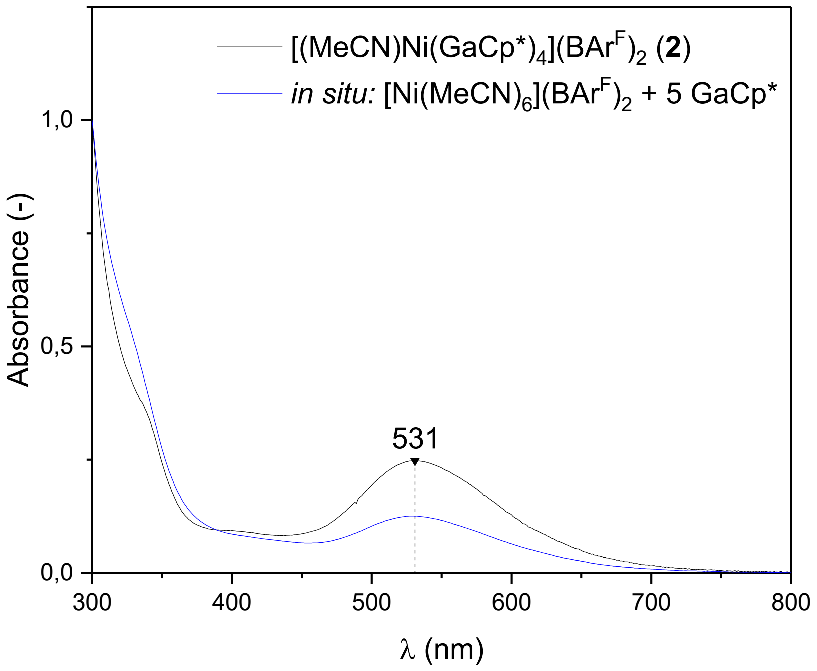

Figure S42: Comparison of the UV-Vis spectra of $[(MeCN)Ni(GaCp^*)_4](BAr^F)_2$ (**2**) and the reaction solution of $[Ni(MeCN)_6](BAr^F)_2$ and GaCp* as approx. 50 µM solutions in 1,2-difluorobenzene. Both spectra share the characteristic absorption band of **2** at around 531 nm, which indicates the presence of $[(MeCN)Ni(GaCp^*)_4](BAr^F)_2$ as the primary product of the reaction of $[Ni(MeCN)_6](BAr^F)_2$ and GaCp*. Both spectra have been normalised with reference to the peak with highest intensity to ensure comparability.

Ideally, the reaction should be a pseudo-first order decay with respect to the absorption band of $[(MeCN)Ni(GaCp^*)_4](BAr^F)_2$ (**2**), as **2** cleanly converts to **1** as the only detectable final product. However, a test reaction with the nickel acetonitrile complex using an excess of GaCp* (to ensure a pseudo-first order reaction with respect to **2**) shows an onset for the first few minutes (see Figure S43). This indicates that **2** is formed comparably fast within the first 20 minutes. The rate for the conversion to **1**, however, seems to be significantly slower.

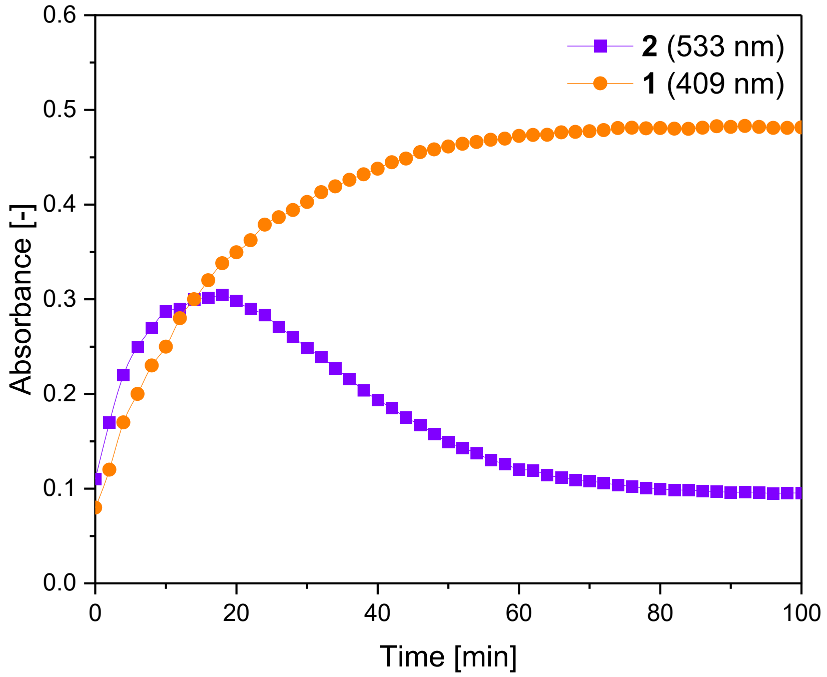

Figure S43: Reaction profile for the formation of **1** starting from ≈10 mg of $[Ni(MeCN)_6](BAr^F)_2$ and an excess (20 mg, ≈20 eq.) of GaCp* in 1,2-difluorobenzene solution at 20 °C. The absorbance was determined at the respective absorption maximum (**2**: 533 nm; **1**: 409 nm) and nicely shows a pseudo-first order reaction with respect to **1** after an induction period of approximately 20 minutes with **2** as the primary product.

As a consequence, the formation of **1** may be described as a two-step reaction with **2** as the primary product:

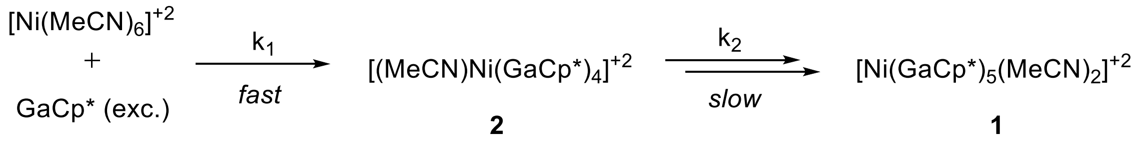

In order to be able to correlate the measured absorbance to a concentration and therefore determine k_{obs} *via* the initial rates method, we determined the extinction coefficient of **2** in 1,2-difluorobenzene solution. For that, the respective amount of **2** (M = 2642.97 g/mol) (0.0045 g, 0.0104 g and 0.0192 g, respectively) was weighed into a 5 mL or 10 mL volumetric flask. After addition of the respective volume of 1,2-difluorobenzene, the absorbance of the solution was determined using UV-Vis spectroscopy (see Table S1). Different batches of **2** were used to ensure comparability. The molar extinction coefficient ε was calculated to be 5669 (± 82) L mol^{-1} cm^{-1} based on these data after linear regression (see Figure S44).

Table S1: Variation of the concentration of **1** in 1,2-difluorobenzene and the corresponding determined absorbance.

**#**	**mg**	**mL**	**c [mg/mL or g/L]**	**c [mol/L]**[a]	**A (a.u.)** λ = 533 nm
1	-	-	-	-	-
2	4.5	10	0.45	1.70×10^{-4}	0.07644[b]
3	10.4	5	2.08	7.87×10^{-4}	0.45793[b]
4	19.2	5	3.84	1.45×10^{-3}	0.81967[b]

[a]M = 2642.97 g/mol; [b]spectra see Figure S45.

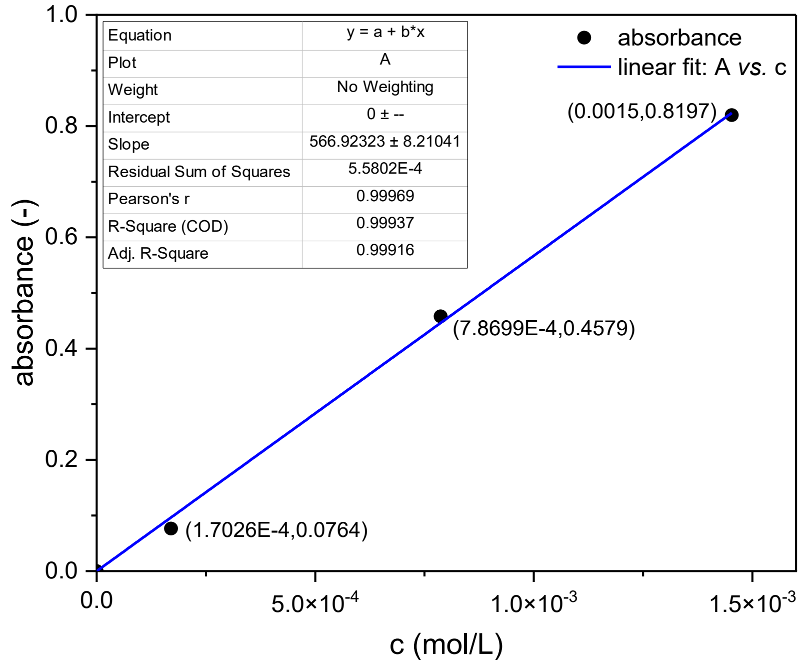

Figure S44: Linear regression for determination of the molar extinction coefficient ε for **2** using stock solutions with differing concentrations.

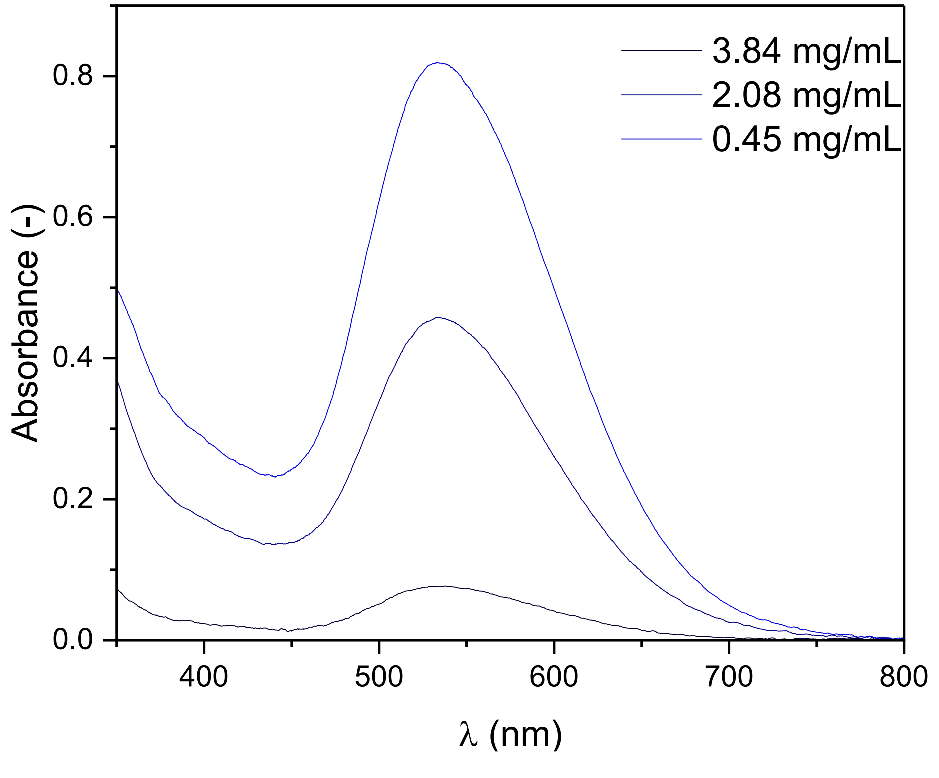

Figure S45: UV-Vis spectra of different stock solutions of compound **2** in 1,2-difluorobenzene solution for determining the molar extinction coefficient ε.

Analogously, we determined the extinction coefficient of **1** in 1,2-difluorobenzene solution by weighing the respective amount of **1** (M = 2889.99 g/mol) (0.0055 g, 0.0056 g and 0.0108 g, respectively) was weighed into a 5 mL or 10 mL volumetric flask. After addition of the respective volume of 1,2-difluorobenzene, the absorbance of the solution was determined using UV-Vis spectroscopy (see

Table S1). The molar extinction coefficient ε was calculated to be 5192 (± 88) L mol^{-1} cm^{-1} based on these data after linear regression (see Figure S46).

Table S2: Variation of the concentration of **1** in 1,2-difluorobenzene and the corresponding determined absorbance.

#	mg	mL	c [mg/mL or g/L]	c [mol/L][a]	A (a.u.) λ = 409 nm
1	-	-	-	-	-
2	5.6	10	0.56	1.94×10^{-4}	0.11091[b]
3	10.8	5	2.16	7.47×10^{-4}	0.20369[b]
4	5.5	5	3.88	1.34×10^{-3}	0.38231[b]

[a]M = 2889.99 g/mol; [b]spectra see Figure S47.

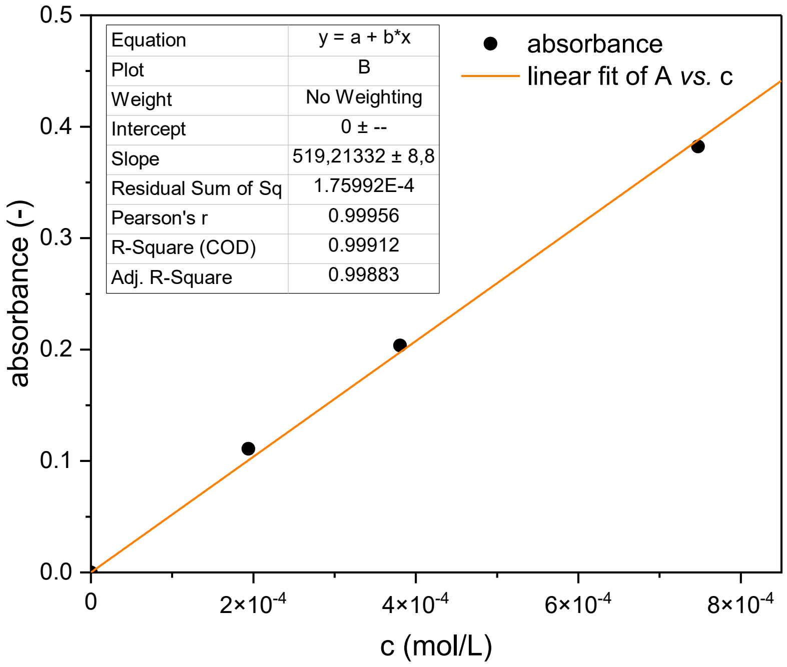

Figure S46: Linear regression for determination of the molar extinction coefficient ε for **1** using stock solutions with differing concentrations.

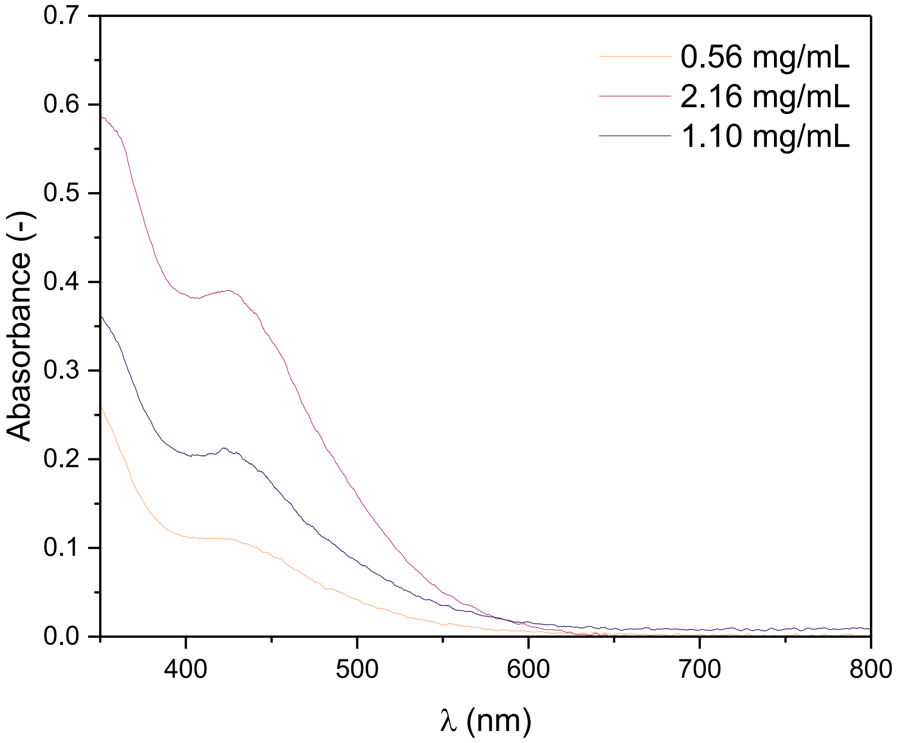

Figure S47: UV-Vis spectra of different stock solutions of compound **1** in 1,2-difluorobenzene solution for determining the molar extinction coefficient ε.

6. DFT Calculations

Details on the calculated mechanism & optimized geometries

We calculated the Gibbs free energy for potential key intermediates (without BArF anions) for the dimerization of acetonitrile, starting with $[(MeCN)Ni(GaCp^*)_4](BAr^F)_2$ (**2**) as the initial species. Therefore, all calculated Gibbs free energies refer to **2** (Figure S48), one free GaCp* and one acetonitrile moiety. Overall, the whole mechanism resembles a redox neutral process that occurs at gallium. The first step is predicted to be exergonic (–5.0 kcal mol-1) and resembles the coordination of acetonitrile to a GaCp* moiety of **2**, forming $[(MeCN)Ni(GaCp^*)_4MeCN]^{2+}$ (**I**). Noteworthy, a change from triplet to singlet state results in nitrile migration from the nickel atom in $[(MeCN)_2Ni(GaCp^*)_4]^{2+}$ (triplet, 20-electron complex) to a GaCp* moiety to form $[(MeCN)Ni(GaCp^*\text{-}NCMe)(GaCp^*)_3]^{2+}$ (**I**, singlet, 18-electron; Figure S 49). Migratory insertion of the acetonitrile moiety into a Ga–Cp* bond then produces an imido-like species **II** (ΔG_R = 7.2 kcal mol^{-1}), whose subsequent tautomerization to an enamide-like species **III** is exergonic (ΔG_R = –2.1 kcal mol^{-1}, see Figure S50). Transition state calculations, however, suggest an extraordinarily high barrier (TS2) of 34.6 kcal mol^{-1} compared to **III**. This points toward proton tunneling in the transition state, which is consistent with the determined abnormally large KIE of 28 for H *vs.* D. In this regard, the thermodynamics of this tunneling process were also calculated for the deuterated analogue, suggesting both the transition state geometry and energy are strikingly similar for both hydrogen and deuterium. Following the tautomerization, coordination of a second acetonitrile moiety to the reactive gallium center yields **IV** (ΔG_R = 15.3 kcal mol^{-1}), which undergoes a nucleophilic attack of the enamide-type ligand to the α-carbon atom of the nitrile. This leads to C–C bond formation to produce the β diketiminate species **V** (ΔG_R = 5.4 kcal mol^{-1}, see Figure S51) *via* TS3 (–7.6 kcal mol^{-1}). TS3 is remarkable in that the enamide moiety adopts a bridging mode between two gallium centers. In the course, the β-diketiminate species **V** may undergo another tautomerization step to form the nacnac-type ligand **VI** bearing two N–H moieties (ΔG_R = –23.4 kcal mol^{-1}, see Figure S52) as observed in the solid state structure of $[Ni(GaCp^*)_4(GaN_2C_{14}H_{21})](BAr^F)_2$ (**1**,). This transformation proceeds analogously to TS2 *via* a large activation barrier of 44.6 kcal mol^{-1}, indicating proton tunneling. After coordination of a fifth GaCp* moiety to the nickel center, **1** is formed as the final product (ΔG_R = –54.1 kcal mol^{-1}, see Figure S52). We also tested whether proton relay steps including one additional acetonitrile or 1,2-difluorobenzene molecule are viable pathways for the two tautomerization steps in TS2 as well as TS4 (see Figures S45 and 46). However, the activation energy barriers even increased acetonitrile (TS2: ΔG = 35.5 kcal mol^{-1}; TS4: ΔG = 48.0 kcal mol^{-1}) and significantly for 1,2-difluorobenzene (TS2: ΔG = 43.4 kcal mol^{-1}; TS4: ΔG = 82.2 kcal mol^{-1}). Therefore, solvent molecules acting as proton shuttles appear to be unfeasible in this regard and proton tunneling may play a crucial role for these steps. Furthermore, calculations on the formation of the deuterated product $\mathbf{1^d}$ reveal no significant differences in transition state energies for the two tautomerization steps (see Table S3).

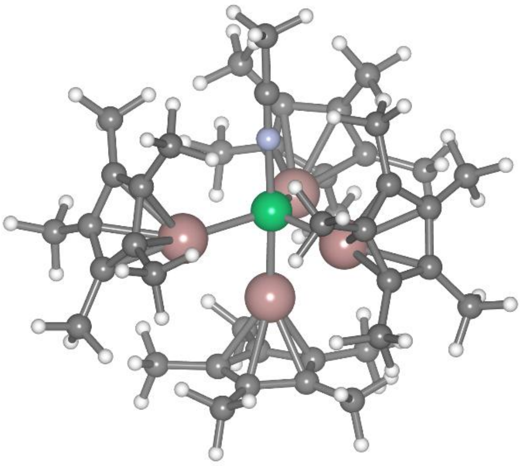

Figure S48: DFT-optimized geometry of **2**.

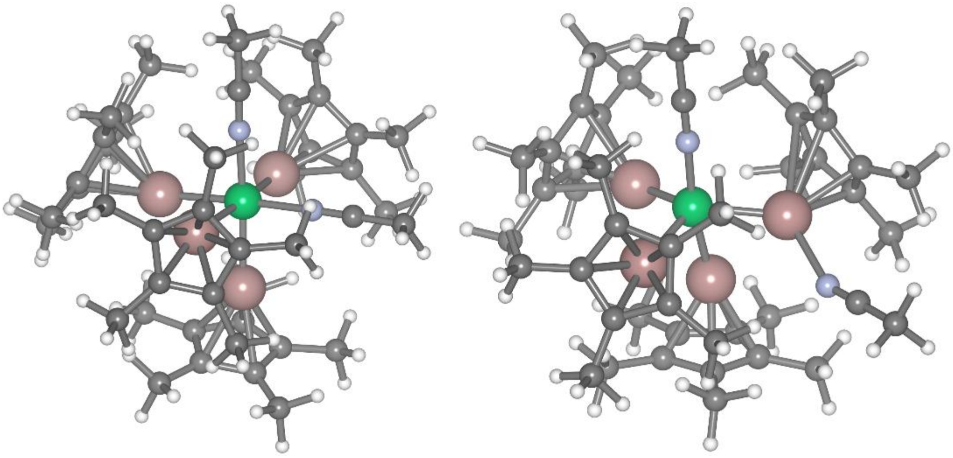

Figure S 49: DFT-optimized geometries of **I** in triplet (left) and singlet state (right).

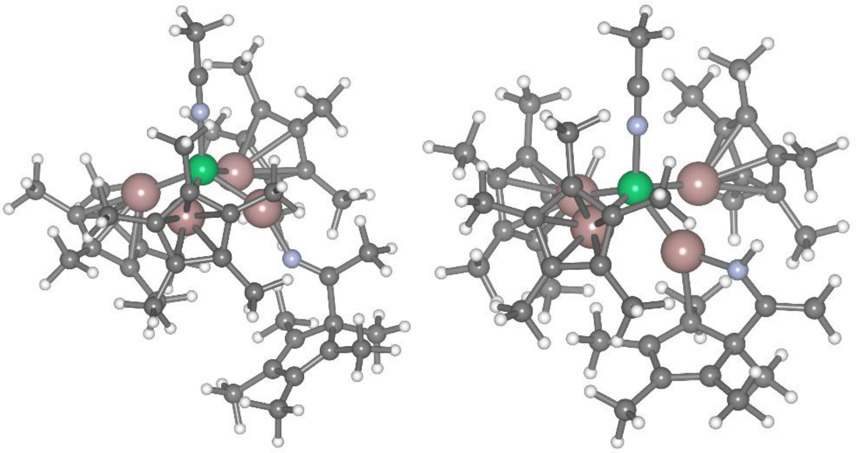

Figure S50: DFT-optimized geometries of **II** (left) and **III** (right).

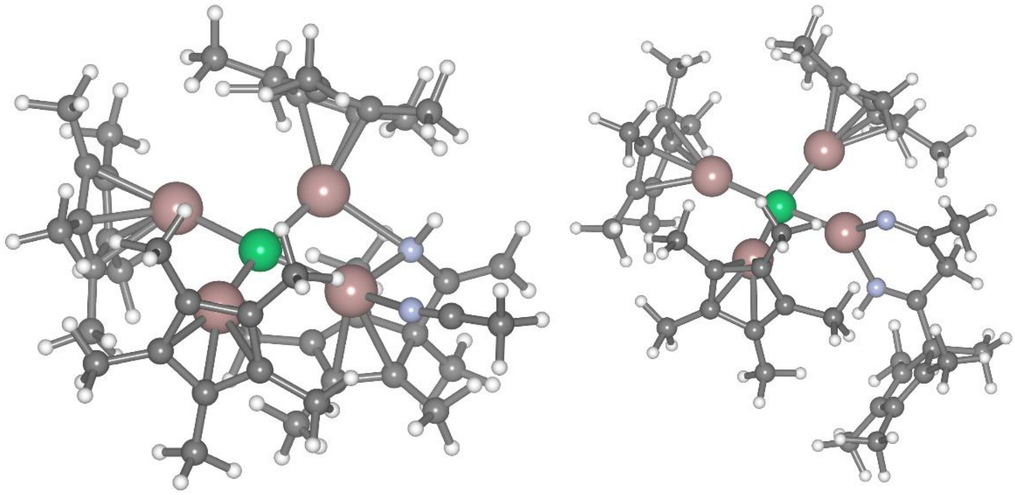

Figure S51: DFT-optimized geometries of **IV** (left) and **V** (right).

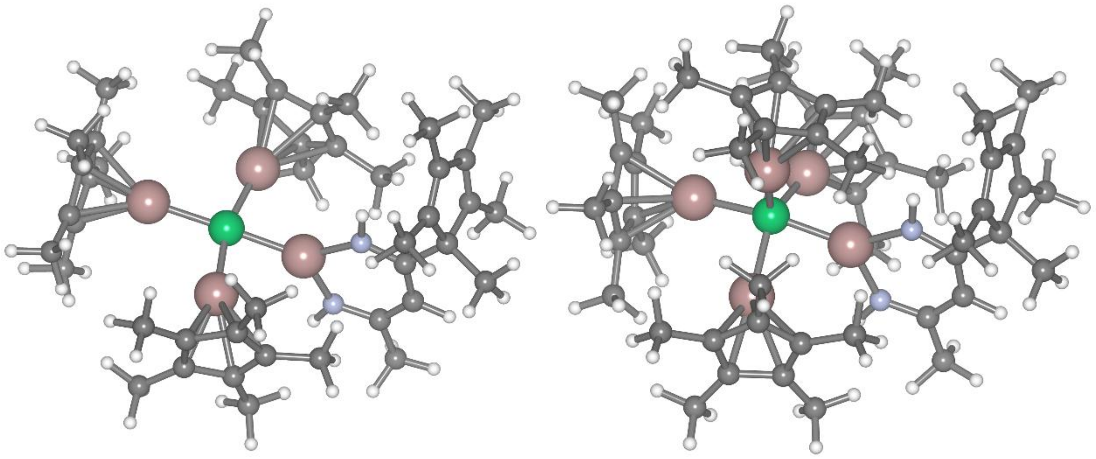

Figure S52: DFT-optimized geometries of **VI** (left) and **1** (right).

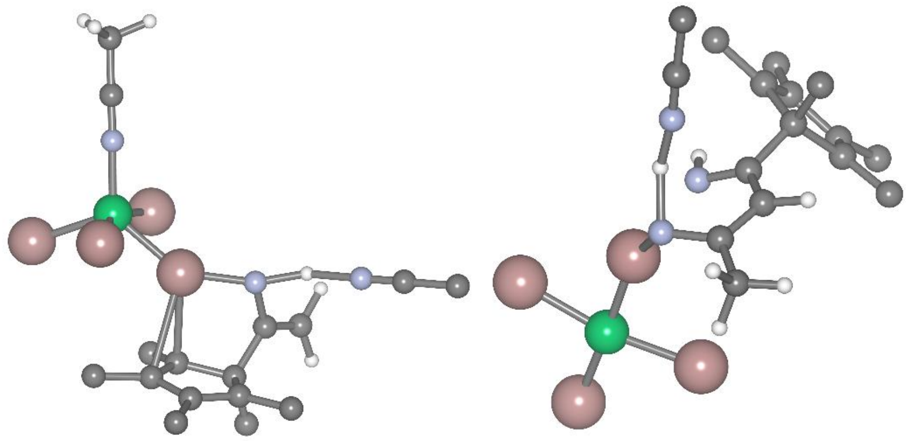

Figure S53: DFT-optimized geometry of TS2 (left) and TS4 (right) with one additional acetonitrile molecule acting as a proton shuttle during tautomerization. Cp* ligands have been omitted for clarity.

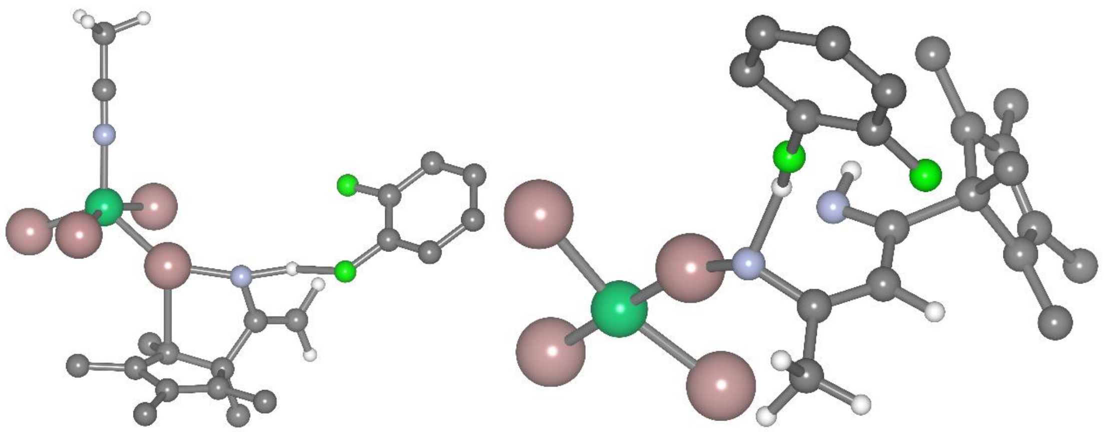

Figure S54: DFT-optimized geometry of TS2 (left) and TS4 (right) with one additional 1,2-difluorobenzene molecule acting as a proton shuttle during tautomerization. Cp* ligands have been omitted for clarity.

Table S3: Calculated energy differences for the two tautomerization steps **II** → **TS2** → **III** and **V** → **TS4** → **VI** for the non-deuterated product **1** as well as the deuterated congener **1**^{d}.

	**1**	**1**^{d}	**Δ(ΔG$_{R}$)**
**II**	0	0	-
**TS2**	56.17	57.20	1.03
**III**	–9.29	–9.33	–0.04
**V**	0	0	-
**TS4**	50.01	50.90	0.89
**VI**	–28.76	–28.88	–0.12

In silico **replacement of GaCp* for carbon monoxide and acetonitrile**

As indicated by the optimized geometries of **IV** and TS3, which feature the enamide moiety in a bridging mode between two Ga atoms, the whole "all-gallium" coordination sphere actively participates in the dimerization of acetonitrile. In order to showcase the importance of the gallium-rich environment for this reactivity, we focused on the first step of the mechanism – the migratory insertion of acetonitrile into the Ga–Cp* bond – and replaced *in silico* two of the four GaCp* moieties of **I** for spectator ligands. We chose carbon monoxide and acetonitrile for this purpose as they are isoelectronic to GaCp* and compared all three complexes – "all-gallium" **I** and replaced by either two carbon monoxide (**I$_{CO}$**) or two acetonitrile ligands (**I$_{MeCN}$**) – regarding the first migratory insertion step (**I** → **II**). While the geometry of the transition state and the HOMO and LUMO orbitals of all three complexes remain similar (see figures S38 to S40), the energy barriers for the transition state (TS1) change significantly for both the CO and MeCN congener (see Figure S58). The barrier increases to 24.7 kcal mol^{-1} for **I$_{CO}$** and for **I$_{MeCN}$** to 21.0 kcal mol^{-1}. For comparison, TS1 has been calculated to be 19.0 kcal mol^{-1} for the "all-gallium" congener **I**. In addition, the HOMO-LUMO gaps for the calculated intermediates containing CO and MeCN increase significantly from 1.760 eV (**I**) to 2.208 eV for **I$_{CO}$** and 2.114 eV for **I$_{MeCN}$**, respectively. Therefore, nitrile dimerization is exclusively promoted with an all-GaCp* coordination sphere around the nickel center.

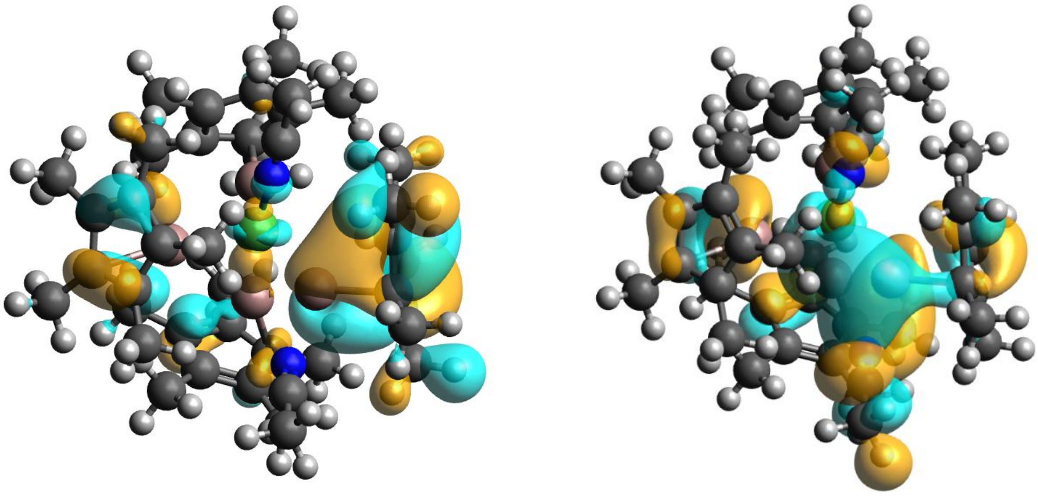

Figure S55: HOMO-2 (left) and LUMO+1 (right) orbitals of intermediate **I** during the formation of $[Ni(GaCp^*)_4(GaN_2C_{14}H_{21})]^{2+}$ showing electron density located at the Cp* ligand, which is necessary for insertion of acetonitrile during transition from **I** to **II**.

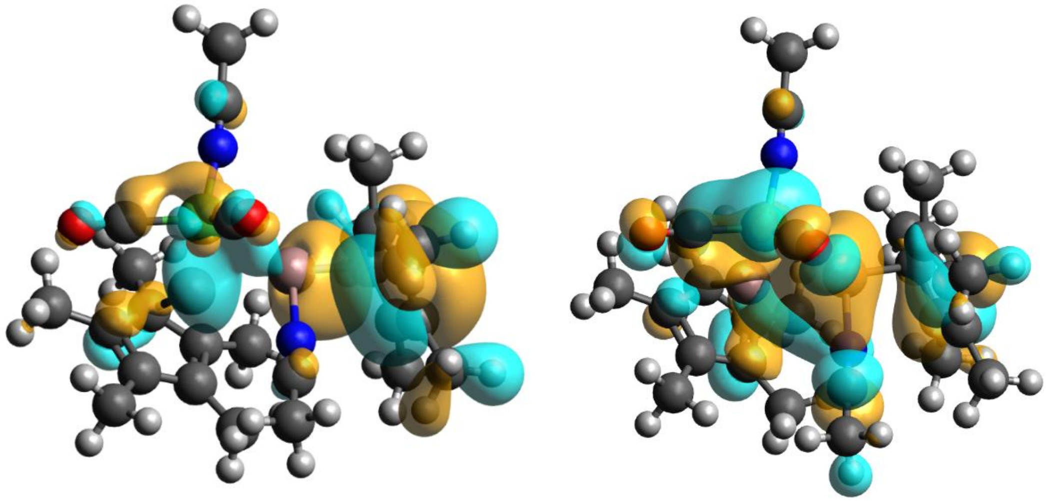

Figure S56: HOMO-1 (left) and LUMO (right) orbitals of intermediate $\mathbf{I_{CO}}$ during the formation of $[Ni(GaCp^*)_2(CO)_2(GaN_2C_{14}H_{21})]^{2+}$ showing electron density located at the Cp* ligand, which is necessary for insertion of acetonitrile during transition from $\mathbf{I_{CO}}$ to $\mathbf{II_{CO}}$.

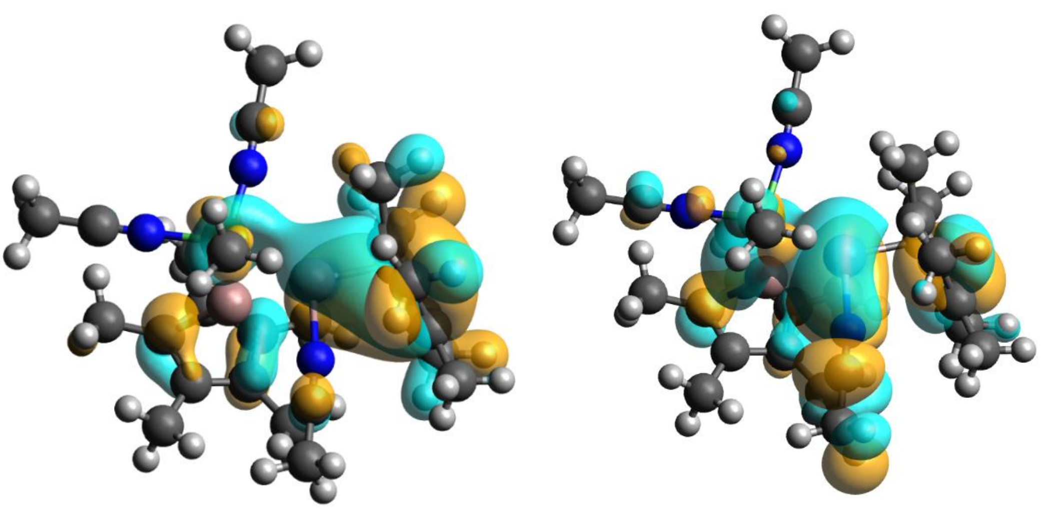

Figure S57: HOMO-3 (left) and LUMO+1 (right) orbitals of intermediate $\mathbf{I_{MeCN}}$ during formation of $[Ni(GaCp^*)_2(MeCN)_2(GaN_2C_{14}H_{21})]^{2+}$ showing electron density located at the Cp* ligand, which is necessary for insertion of acetonitrile during transition from $\mathbf{I_{MeCN}}$ to $\mathbf{II_{MeCN}}$.

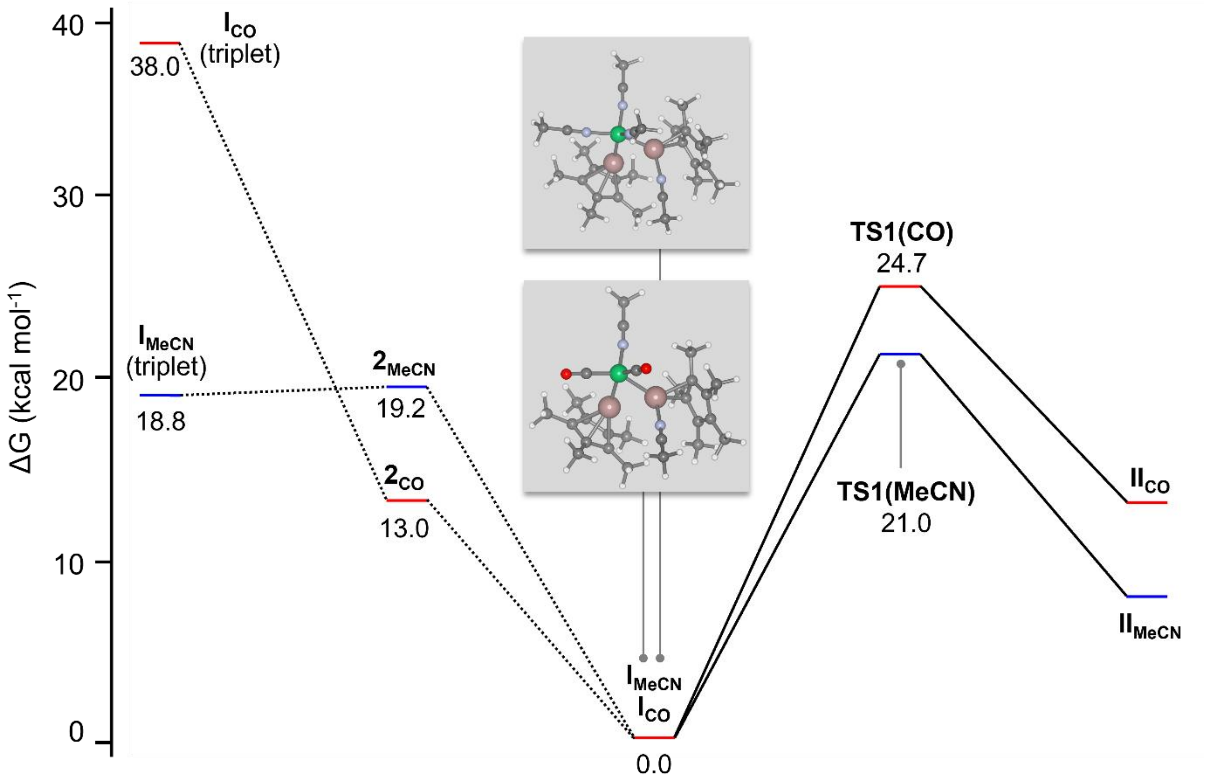

Figure S58: Energy profile for the CO and MeCN congeners $\mathbf{I_{CO}}$ and $\mathbf{I_{MeCN}}$ regarding the first migratory insertion step *via* TS1. Compared to the "all-gallium" **I**, the activation energy barriers increase significantly.

That nitrile dimerization is facilitated in the all-Ga case is also reflected by the Mulliken charges calculated for the species **I**, $\mathbf{I_{CO}}$ and $\mathbf{I_{MeCN}}$ and the corresponding first insertion step of acetonitrile into the Ga–Cp* bond (see Table S4). Overall, the nickel atom in "all-gallium" **I** is significantly more negatively charged than for $\mathbf{I_{CO}}$ and $\mathbf{I_{MeCN}}$. At the same time, Ga1 (which is the reactive center for nitrile dimerization) has a more positive partial charge, which is nicely in line with the high Lewis acidity of **I** and [(MeCN)Ni(GaCp*)$_4$](BAr^F)$_2$ (**2**) that promotes the insertion of acetonitrile for the "all-Ga" **I**.

Table S4: Calculated Mulliken charges for the "all-Ga", CO and MeCN substituted species regarding the intermediates **I**, **II** and the transition state TS1.

	**all-Ga**	**CO**	**MeCN**
**I**	Ni: –1.678<br>Ga1: 0.457<br>Ga2: 0.404	Ni: –0.241<br>Ga1: 0.009<br>Ga2: 0.546	Ni: –0.251<br>Ga1: 0.131<br>Ga2: 0.510
**TS1** (insertion step)	Ni: –0.664<br>Ga1: 0.190<br>Ga2: 0.355	Ni: –0.353<br>Ga1: 0.409<br>Ga2: 0.518	Ni: –0.233<br>Ga1: 0.339<br>Ga2: 0.432
**II**	Ni: –0.494<br>Ga1: –0.183<br>Ga2: 0.161	Ni: –0.233<br>Ga1: 0.339<br>Ga2: 0.432	Ni: –0.275<br>Ga1: 0.344<br>Ga2: 0.488

7. References

(1) Smith, C. R.; Aibin Zhang; Mans, D. J.; V., T.; RajanBabu. (R)-3-Methyl-3-phenyl-1-pentene via catalytic asymmetric hydrovinylation. *Org. Synth.* **2008**, *85*, 248-266. DOI: 10.15227/orgsyn.085.0248.

(2) Useful Reagents and Ligands. In *Inorganic Syntheses*, 2002; pp 75-121.

(3) Yakelis, N. A.; Bergman, R. G. Safe Preparation and Purification of Sodium Tetrakis[(3,5-trifluoromethyl)phenyl]borate ($NaBAr^{F24}$): Reliable and Sensitive Analysis of Water in Solutions of Fluorinated Tetraarylborates. *Organometallics* **2005**, *24* (14), 3579-3581. DOI: 10.1021/om0501428.

(4) Leazer, J. L.; Cvetovich, R.; Tsay, F.-R.; Dolling, U.; Vickery, T.; Bachert, D. An Improved Preparation of 3,5-Bis(trifluoromethyl)acetophenone and Safety Considerations in the Preparation of 3,5-Bis(trifluoromethyl)phenyl Grignard Reagent. *J. Org. Chem.* **2003**, *68* (9), 3695-3698. DOI: 10.1021/jo026903n.

(5) Brookhart, M.; Grant, B.; Volpe, A. F., Jr. $[(3,5\text{-}(CF_3)_2C_6H_3)_4B]^-[H(OEt_2)_2]^+$: a convenient reagent for generation and stabilization of cationic, highly electrophilic organometallic complexes. *Organometallics* **1992**, *11* (11), 3920-3922. DOI: 10.1021/om00059a071.

(6) Chávez, I.; Alvarez-Carena, A.; Molins*, E.; Roig, A.; Maniukiewicz, W.; Arancibia, A.; Arancibia, V.; Brand, H.; Manuel Manríquez*, J. Selective oxidants for organometallic compounds containing a stabilising anion of highly reactive cations: $(3,5(CF_3)_2C_6H_3)_4B^-)Cp_2Fe^+$ and $(3,5(CF_3)_2C_6H_3)_4B^-)Cp^*_2Fe^+$. *J. Organomet. Chem.* **2000**, *601* (1), 126-132. DOI: https://doi.org/10.1016/S0022-328X(00)00044-9.

(7) De Souza, R. F.; Monteriro, A. L.; Seferin, M.; De Souza, M. O.; Stedile, F. C.; Wyrvalski, C. N.; Baumvol, I. J. R. Synthesis and characterization of dicationic nickel complexes. *J. Coord. Chem.* **1996**, *40* (4), 311-318. DOI: 10.1080/00958979608024535.

(8) Alberti, D.; Pörschke, K.-R. Thallium Perfluorotetraphenylborate. *Organometallics* **2004**, *23* (6), 1459-1460. DOI: 10.1021/om0306729.

(9) *The National Institute for Occupational Safety and Health (NIOSH) - Thallium (soluble compounds, as Tl)*. https://www.cdc.gov/niosh/npg/npgd0608.html (accessed 2025 05/20/2025).

(10) Jutzi, P.; Schebaum, L. O. A novel synthetic route to pentaalkylcyclopentadienylgallium(I) compounds. *J. Organomet. Chem.* **2002**, *654* (1), 176-179. DOI: https://doi.org/10.1016/S0022-328X(02)01429-8.

(11) Green, M. L. H.; Mountford, P.; Smout, G. J.; Speel, S. R. New synthetic pathways into the organometallic chemistry of gallium. *Polyhedron* **1990**, *9* (22), 2763-2765. DOI: https://doi.org/10.1016/S0277-5387(00)86809-6.

(12) Gutmann, V. Solvent effects on the reactivities of organometallic compounds. *Coordination Chemistry Reviews* **1976**, *18* (2), 225-255. DOI: https://doi.org/10.1016/S0010-8545(00)82045-7.

(13) Beckett, M. A.; Strickland, G. C.; Holland, J. R.; Sukumar Varma, K. A convenient n.m.r. method for the measurement of Lewis acidity at boron centres: correlation of reaction rates of Lewis acid initiated epoxide polymerizations with Lewis acidity. *Polymer* **1996**, *37* (20), 4629-4631. DOI: https://doi.org/10.1016/0032-3861(96)00323-0.

(14) Zuur, A. P.; Reintjes, A. H. L.; Groeneveld, W. L. Complexes with ligands containing nitrile groups.: Part VIII. Organic nitriles as ligand. *Recl. Trav. Chim. Pays-Bas* **1970**, *89* (4), 385-391. DOI: https://doi.org/10.1002/recl.19700890407.

(15) Bougon, R.; Charpin, P.; Christe, K. O.; Isabey, J.; Lance, M.; Nierlich, M.; Vigner, J.; Wilson, W. W. Preparation and characterization of nickel(2+) hexafluorobismuthate(1-) and of the ternary adducts $[Ni(CH_3CN)_6](BiF_6)_2$ and $[Ni(CH_3CN)_6](SbF_6)_2$. Crystal structure of hexakis(acetonitrile-d_3)nickel(2+) hexafluoroantimonate. *Inorg. Chem.* **1988**, *27* (8), 1389-1393. DOI: 10.1021/ic00281a018.

Crystallography

C1. *APEX4*, Version 2021, Bruker AXS Inc., Madison, Wisconsin, USA, 2021.

C2. *SAINT*, Version 2021, Bruker AXS Inc., Madison, Wisconsin, USA, 2021.

C3. *SADABS*, Version 2021, Bruker AXS Inc., Madison, Wisconsin, USA, 2021.

C4. G. M. Sheldrick, *Acta Crystallogr. Sect. A*, 2015, **71**, 3–8.

C5. G. M. Sheldrick, *Acta Crystallogr. Sect. C*, 2015, **71**, 3–8.

C6. C. B. Hübschle, G. M. Sheldrick, B. Dittrich, *J. Appl. Cryst.*, 2011, **44**, 1281–1284.

C7. *International Tables for Crystallography*, Vol. C (Ed.: A. J. Wilson), Kluwer Academic Publishers, Dordrecht, The Netherlands, 1992, Tables 6.1.1.4 (pp. 500–502), 4.2.6.8 (pp. 219–222), and 4.2.4.2 (pp. 193–199).

C8. D. Kratzert, J. J. Holstein, I. Krossing, *J. Appl. Cryst.*, 2015, **48**, 933–938.

C9. A. L. Spek, *Acta Crystallogr. Sect. C*, 2015, **71**, 9–18.

C10. A. L. Spek, *Acta Crystallogr. Sect. D*, 2009, **65**, 148–155.

C11. C. F. Macrae, I. J. Bruno, J. A. Chisholm, P. R. Edgington, P. McCabe, E. Pidcock, L. Rodriguez-Monge, R. Taylor, J. van de Streek, P. A. Wood, *J. Appl. Cryst.*, 2008, **41**, 466–470.

C12. D. Kratzert, *FinalCif*, *V151*, https://dkratzert.de/finalcif.html.
